# Proposal of a familial hypercholesterolemia paediatric diagnostic score (FH-PeDS)

**DOI:** 10.1093/eurjpc/zwaf352

**Published:** 2025-06-20

**Authors:** Jan Kafol, Beatriz Miranda, Rok Sikonja, Jaka Sikonja, Albert Wiegman, Ana Margarida Medeiros, Ana Catarina Alves, Tomas Freiberger, Barbara A Hutten, Matej Mlinaric, Tadej Battelino, Steve E Humphries, Mafalda Bourbon, Urh Groselj

**Affiliations:** Faculty of Medicine, University of Ljubljana, Vrazov trg 2, Ljubljana 1000, Slovenia; Department of Vascular Diseases, Division of Internal Medicine, University Medical Centre Ljubljana, Ljubljana 1000, Slovenia; Grupo de Investigação Cardiovascular, Unidade I&D Departamento de Promoção da Saúde e Doenças Crónicas, Instituto Nacional de Saúde Doutor Ricardo Jorge, Avenida Padre Cruz, Lisboa 1649-016, Portugal; BioISI–Biosystems and Integrative Sciences Institute, Faculty of Sciences, University of Lisboa, Campo Grande, Lisboa 1749-016, Portugal; LatticeFlow AI, Zürich 8005, Switzerland; Faculty of Medicine, University of Ljubljana, Vrazov trg 2, Ljubljana 1000, Slovenia; Department of Endocrinology, Diabetes and Metabolic Diseases, Division of Internal Medicine, University Medical Centre Ljubljana, Ljubljana 1000, Slovenia; Department of Pediatrics, Amsterdam University Medical Centers, University of Amsterdam, Amsterdam 1105, The Netherlands; Grupo de Investigação Cardiovascular, Unidade I&D Departamento de Promoção da Saúde e Doenças Crónicas, Instituto Nacional de Saúde Doutor Ricardo Jorge, Avenida Padre Cruz, Lisboa 1649-016, Portugal; BioISI–Biosystems and Integrative Sciences Institute, Faculty of Sciences, University of Lisboa, Campo Grande, Lisboa 1749-016, Portugal; Grupo de Investigação Cardiovascular, Unidade I&D Departamento de Promoção da Saúde e Doenças Crónicas, Instituto Nacional de Saúde Doutor Ricardo Jorge, Avenida Padre Cruz, Lisboa 1649-016, Portugal; BioISI–Biosystems and Integrative Sciences Institute, Faculty of Sciences, University of Lisboa, Campo Grande, Lisboa 1749-016, Portugal; Centre for Cardiovascular Surgery and Transplantation, and Medical Faculty, Masaryk University, Brno 602 00, Czech Republic; Amsterdam Cardiovascular Sciences, Amsterdam University Medical Center, University of Amsterdam, Amsterdam 1105, The Netherlands; Department of Epidemiology and Data Science, Amsterdam University Medical Center, Location University of Amsterdam, Amsterdam 1105, The Netherlands; Faculty of Medicine, University of Ljubljana, Vrazov trg 2, Ljubljana 1000, Slovenia; Department of Endocrinology, Diabetes, and Metabolic Diseases, University Children’s Hospital, University Medical Centre Ljubljana, Bohoriceva 20, Ljubljana 1000, Slovenia; Faculty of Medicine, University of Ljubljana, Vrazov trg 2, Ljubljana 1000, Slovenia; Department of Endocrinology, Diabetes, and Metabolic Diseases, University Children’s Hospital, University Medical Centre Ljubljana, Bohoriceva 20, Ljubljana 1000, Slovenia; Institute of Cardiovascular Science, Faculty of Population Health, University College London, London WC1E 6BT, UK; Grupo de Investigação Cardiovascular, Unidade I&D Departamento de Promoção da Saúde e Doenças Crónicas, Instituto Nacional de Saúde Doutor Ricardo Jorge, Avenida Padre Cruz, Lisboa 1649-016, Portugal; BioISI–Biosystems and Integrative Sciences Institute, Faculty of Sciences, University of Lisboa, Campo Grande, Lisboa 1749-016, Portugal; Faculty of Medicine, University of Ljubljana, Vrazov trg 2, Ljubljana 1000, Slovenia; Department of Endocrinology, Diabetes, and Metabolic Diseases, University Children’s Hospital, University Medical Centre Ljubljana, Bohoriceva 20, Ljubljana 1000, Slovenia

**Keywords:** Familial hypercholesterolemia, Diagnostic criteria, Detection, Machine learning model, Cardiovascular disease, Children

## Abstract

**Aims:**

Familial hypercholesterolemia (FH) significantly increases cardiovascular risk from childhood yet remains widely underdiagnosed. This cross-sectional study aimed to evaluate existing paediatric FH diagnostic criteria in real-world cohorts and to develop two novel diagnostic tools: a semi-quantitative scoring system (FH-PeDS) and a machine learning model (ML-FH-PeDS) to enhance early FH detection.

**Methods and results:**

Five established FH diagnostic criteria were assessed (Dutch Lipid Clinics Network [DLCN], Simon Broome, EAS, Simplified Canadian, and Japanese Atherosclerosis Society) in Slovenian (*N* = 1360) and Portuguese (*N* = 340) paediatric hypercholesterolemia cohorts, using FH-causing variants as the reference standard. FH-PeDS was developed from the Slovenian cohort, and ML-FH-PeDS was trained and tested using a 60%/40% split before external validation in the Portuguese cohort. Only 47.4% of genetically confirmed FH cases were identified by all established criteria, while 10.9% were missed entirely. FH-PeDS outperformed DLCN in the combined cohort (AUC 0.897 vs. 0.857; *P* < 0.01). ML-FH-PeDS showed superior predictive power (AUC 0.932 in training, 0.904 in testing vs. 0.852 for DLCN; *P* < 0.01) and performed best as a confirmatory test in the testing subgroup (39.7% sensitivity, 87.7% PPV at 98% specificity). In the Portuguese cohort, ML-FH-PeDS maintained strong predictive performance (AUC 0.867 vs. 0.815 for DLCN; *P* < 0.01) despite population differences.

**Conclusion:**

Current FH diagnostic criteria perform sub-optimally in children. FH-PeDS and ML-FH-PeDS provide tools to improve FH detection, particularly where genetic testing is limited. They also help guide genetic testing decisions for hypercholesterolemic children. By enabling earlier diagnosis and intervention, these tools may reduce long-term cardiovascular risk and improve outcomes.

## Graphical Abstract

**Graphical Abstract:**
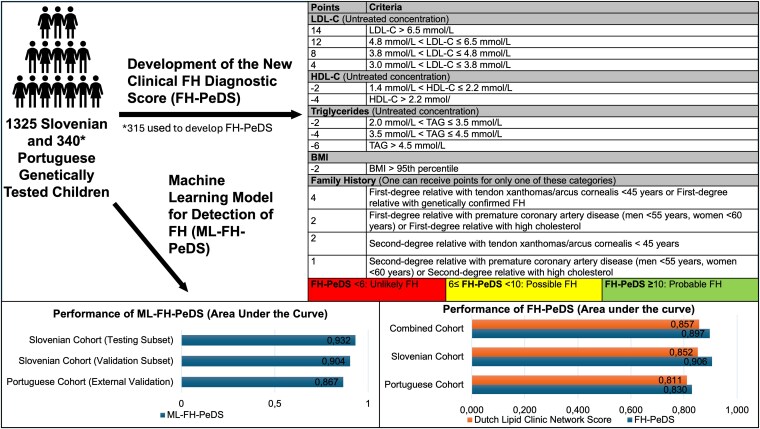

**See the editorial comment for this article ‘Screening for familial hypercholesterolaemia: children are not small adults’, by V. Bittner, https://doi.org/10.1093/eurjpc/zwaf565.**

## Introduction

Familial hypercholesterolemia (FH) is the most common inherited metabolic disorder, leading to life-threatening cardiovascular (CV) complications.^1,2^ Individuals with FH have elevated low-density lipoprotein cholesterol (LDL-C) concentrations from birth, which significantly raises the risk of pre-mature atherosclerotic CV disease (ASCVD).^3^ Numerous clinical and genetic studies underscore the central role of LDL-C in ASCVD, emphasizing early detection and management, particularly given that CV disease remains the leading cause of death worldwide.^4,5^ Because atherosclerosis begins early and progresses over decades, maintaining optimal lipid concentrations from childhood is crucial for effective primary prevention.^6,7^

Heterozygous FH affects about 1 in 300 individuals, amounting to 14–34 million cases worldwide, yet less than 1% are diagnosed.^3,8^ Alarmingly, only 2.1% of adults with FH were identified in childhood, despite the well-established benefits of early lipid-lowering interventions.^9–12^ This gap highlights an urgent need for childhood screening strategies that are practical within each nation’s healthcare framework.^13–15^ Although genetic testing is the gold standard, most countries rely primarily on clinical methods, largely due to cost considerations.^10,16,17^ Moreover, although serum cholesterol concentrations are routinely used in CV risk assessments, they alone cannot reliably distinguish patients most likely to have FH.^18^

To enhance diagnostic accuracy, tools such as the Dutch Lipid Clinics Network (DLCN), Simon Broome (SB), and make early diagnosis to prevent early deaths (MEDPED) criteria are recommended.^19–22^ This is particularly relevant in resource-limited settings, where financial constraints restrict access to genetic testing, necessitating targeted screening, primarily among children of parents already diagnosed with FH.^10,15^ However, most established FH diagnostic scores were developed and validated in adults, limiting their applicability in paediatrics and contributing to the low diagnosis rates among children.^1,23,24^

Currently, no FH diagnostic scoring system has been fully developed for and validated in paediatric populations using real-world clinical data, where limitations due to the unreliability or incompleteness of family history are common. The aims of this study were three-fold: (i) Evaluate existing scores: assess the performance of five established clinical diagnostic scores for FH in children; (ii) develop a new clinical score (FH-PeDS): create a novel paediatric diagnostic score for FH, based on real-world clinical data, to enhance the identification of children likely to have FH who may benefit from genetic testing, and to improve the accuracy of clinical diagnoses in settings where genetic testing is inaccessible or impractical; and (iii) develop a machine learning model (ML-FH-PeDS): develop a data pre-processing pipeline and an interpretable ML-FH-PeDS designed to achieve the highest possible diagnostic accuracy for FH in the children subject to small dataset size, the need for interpretability and low model inference requirements, with plans to make this tool freely and widely available online.

## Methods

### Study design

This cross-sectional study was approved by the Slovenian (SI) National Medical Ethics Committee (No. 22/01/2017; 0120-14/2017-2; 0120-14/2017-5; 0120-100/2019/5; 0120-295/2023/3) and by the Portuguese (PO) National Health Institute Dr Ricardo Jorge (INSA) and the National Data Protection Commission. It was conducted in accordance with the Declaration of Helsinki. Informed consent, including permission for genetic analysis and publication of anonymized data, was obtained from parents or legal guardians. Study reporting followed TRIPOD + AI guidelines.

### Biochemical analysis

Both cohorts underwent laboratory analyses at accredited centres using standardized protocols. In the SI cohort, venous blood samples were collected after overnight fasting at the University Medical Centre Ljubljana. LDL-C was calculated via the Friedewald formula when triglycerides (TAG) were below 4 mmol/L (154.7 mg/dL) or measured directly if above. Total cholesterol (TC), high-density lipoprotein cholesterol (HDL-C), directly measured LDL-C, and TAG were analysed on the Abbott Alinity C (Abbott Laboratories, USA), and Lp(a) was assessed by immunonephelometry using the Siemens Atellica Neph 630 (Siemens Healthineers, Ireland). In the PO cohort, fasting biochemical profiles (TC, LDL-C, HDL-C, TAG, and Lp(a)) were evaluated at INSA using enzymatic and colorimetric methods on the COBAS Integra 400 plus (Roche, Switzerland).

### Genetic analysis

In our study, patients were classified as FH-positive if a pathogenic or likely pathogenic variant was detected in one of the three main FH-associated genes (*LDLR, APOB*, or *PCSK9*). Identified genetic variants were interpreted according to the guidelines of American College of Medical Genetics and Genomics.^25–27^ Genetic analyses were performed at the University Medical Centre Ljubljana for SI cohort and at INSA for PO cohort. A comprehensive description of these methodologies is provided in the Supplementary Materials.

### The Slovenian national familial hypercholesterolemia registry

The registry is built on Slovenia’s universal FH screening programme, introduced in 1995 and previously described.^28,29^ At the primary care level, over 90% of 5-year-olds undergo cholesterol testing, which flags children with total cholesterol ≥6.0 mmol/L (≥232.0 mg/dL) or ≥5.0 mmol/L (≥193.4 mg/dL) plus a positive family history of early CV disease, who are then referred to the Paediatric Lipid Clinic for comprehensive evaluation, including genetic testing. Genetically confirmed FH cases are referred for cascade screening of siblings and parents.

Upon registry entry, family history (high cholesterol, pre-mature CV disease, and tendinous xanthoma or arcus cornealis) is obtained, and all children receive a thorough clinical exam and anthropometric measurements by a paediatric lipid specialist.

### Participants selection

**Figure 1:**
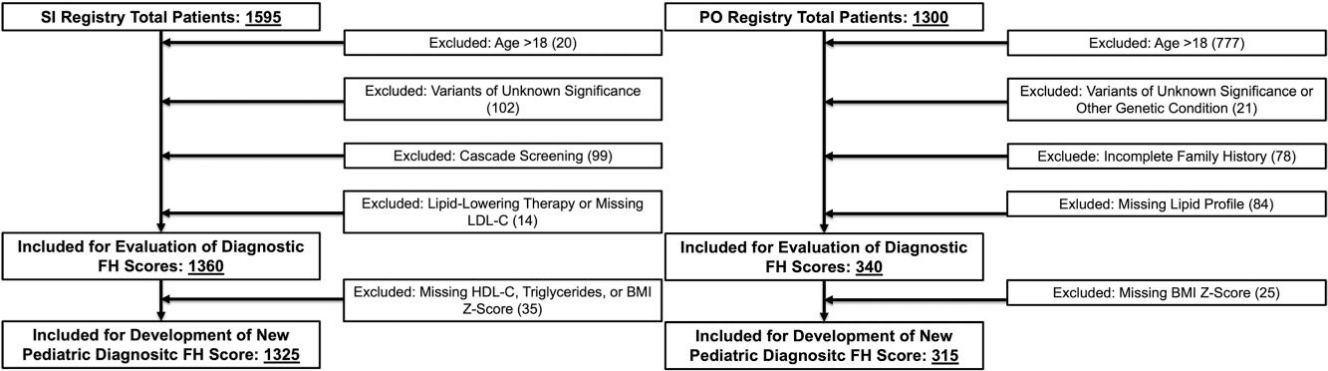
Study inclusion flowchart for Slovenian (SI) and Portuguese (PO) cohort.

We evaluated data from 1595 children in the SI National FH Registry up to July 2024. Of these, 1360 children with genetic analysis and age <18 years were included to assess established FH diagnostic scores; exclusion criteria were variant of uncertain significance (VUS), registry inclusion via cascade screening, missing LDL-C, or lipid-lowering therapy at first exam. For development of the **FH-PeDS** and **ML-FH-PeDS**, individuals missing HDL-C, TAG, or BMI Z-scores were excluded, leaving 1325 participants. *Figure 1* depicts the inclusion and exclusion process.

### Portuguese FH study (EPHF)

The external validation cohort was drawn from the Portuguese FH Study, a nationwide initiative coordinated by INSA since 1999.^30^ The study gathers clinical and molecular data on individuals with a clinical FH diagnosis, referred by clinicians from centres across Portugal. Participation is free for both patients and institutions, and genetic counselling is recommended for all. Individuals meeting the Simon Broome possible or definite FH criteria undergo molecular testing, with cascade screening offered to relatives when a pathogenic variant, likely pathogenic variant, or VUS is identified.^19^ Clinical data, including physical examination, pre-treatment lipid concentrations, therapy usage, cardiovascular history, and family history, are recorded in a confidential database in accordance with legal requirements.

### Validation cohort selection

From 1300 index cases registered between 1999 and March 2024, 523 were under 18. After exclusions for incomplete lipid profiles, missing family history, VUS/other genetic conditions, and missing TAG data, 340 remained for initial analysis and development of **ML-FH-PeDS**. LDL-C was adjusted to pre-treatment concentrations in two subjects on lipid-lowering therapy. For developing **FH-PeDS**, 25 additional subjects were excluded due to missing BMI Z-scores (final *N* = 315). *Figure 1* illustrates the selection process.

### Simplified diagnostic FH scores calculation

In both the SI and PO cohorts, we evaluated five existing clinical scoring systems for FH: SB,^19^ the European Atherosclerosis Society Consensus Panel Pediatric FH Criteria (EAS),^1^ DLCN,^20^ the Simplified Canadian Definition for FH (CAN),^31^ and the Japanese Atherosclerosis Society FH criteria (JAP).^32,33^ Because our focus was on index cases rather than cascade screening, the MEDPED criteria,^22^ were not included. Detailed calculations of each score appear in the Supplementary Materials.

Clinical scoring systems often rely on extensive family history, which may be incomplete in everyday practice. We therefore created simplified versions of each scoring system based on data collected at the first examination. A family history of pre-mature CV disease was defined as an event occurring before age 55 in men or 60 in women, and any family history reported without an exact age was counted as positive. Information on first- and second-degree relatives with heart or vascular disease (peripheral vascular disease and cerebrovascular insult) was recorded separately, and any mention of ‘high cholesterol’ was considered positive—regardless of exact lipid concentrations or potential lipid-lowering therapy use. For systems requiring specific cholesterol values, the reported history of high cholesterol was treated as positive in the absence of measured concentrations.

### Statistical methods

Data were collected in Excel 365 (Microsoft Corporation, USA) and analysed in R version 4.4.1 (R Foundation for Statistical Computing, Austria). Missing values were left blank, and outliers were retained. BMI Z-scores were calculated using the British 1990 reference data with the LMS Growth add-in for Excel (available at: http://www.healthforallchildren.com/shop-base/shop/software/lmsgrowth/).^34,35^ Normality was assessed using the Shapiro–Wilk test; because most variables were non-normally distributed, data are presented as medians with inter-quartile ranges (Q1–Q3). The Mann–Whitney *U* test was used for two-group comparisons. Categorical variables were analysed using the χ^2^ test. All tests were two-tailed, with *P* < 0.05 considered statistically significant, and the Benjamini–Hochberg correction was applied to control false discovery rates.

For analytical consistency, multi-category scores were collapsed into binary classifications by grouping diagnostically relevant categories. In the DLCN system, ‘Definite FH’, ‘Probable FH’, and ‘Possible FH’ were categorized as positive; for SB and CAN, ‘Definite FH’ and ‘Probable FH’ were considered positive; and for JAP and EAS, ‘Probable FH’ was categorized as positive. All other categories were treated as negative. Specificity, sensitivity, positive predictive value (PPV), and negative predictive value (NPV) were calculated using genetic analysis as the reference. Diagnostic accuracy and agreement were assessed using Youden’s Index and Cohen’s Kappa. Receiver operating characteristic (ROC) curves were constructed where appropriate, and comparisons between ROC curves were performed using DeLong’s test.

### Development of the new clinical score (FH-PeDS)

A full description of the methodology is provided in the Supplementary Materials. We developed the **FH-PeDS** in R using a multi-step approach to categorize continuous predictors (LDL-C, HDL-C, TAG, BMI Z-score, Lp(a), etc.) into clinically meaningful cut-offs. We used the SI cohort for model development and the PO cohort for external validation. LOESS plots guided the initial selection of cut-offs, followed by ROC analyses, k-means clustering, and quintile-based methods to confirm natural groupings.^36^ Each numeric predictor was then assigned into categories (e.g. low, moderate, and high), with logistic regression determining the most predictive combination of variables (including sex, age, family history, and physical exam findings). We derived point weights by rounding logistic regression coefficients to the nearest 0.5 and then multiplying by 2, with minimal further adjustments to facilitate practical usage. Negative values were possible for some categories, but the overall score was bounded at a minimum of 0. We assessed model performance by calculating and visualizing the area under the ROC curve (AUC) in both the development (SI) and validation (PO) cohorts.

### Development of machine learning model (ML-FH-PeDS)

The selection and development of the ML model were guided by three key requirements: a relatively small dataset size, the need for interpretability and portability, and the ability to manually evaluate or convert the model into a scoring system. To meet these requirements, a logistic regression model was chosen and developed through three main steps. First, data pre-processing involved imputing missing values using the SI cohort mean, applying feature scaling for continuous features and one-hot encoding for categorical features. Second, data splitting divided the SI cohort into a 60% training set and a 40% test set. Finally, the logistic regression model was trained using L2-regularization with hyperparameters selected via a three-fold cross-validation.

## Results

### Established FH scores validation

#### Overview of subjects’ characteristics

To validate established FH diagnostic scores, we combined data from 1360 subjects in the SI Registry and 340 subjects from the PO Registry as outlined in *Figure 1*. Demographic and clinical data from both registries are presented in *Table 1*. Among included subjects, 496 (29.2%) had a (likely) pathogenic variant in FH-associated genes (FH-positive), while 1204 (70.8%) did not have a (likely) pathogenic variant (FH-negative). The median age of subjects was 6.63 (5.79–9.30) years, with no significant difference between those with and without an FH-causing variant (*P* = 0.32). Females constituted a slightly greater proportion of subjects (57.1%) in both registries; however, among FH-positive individuals, the sex distribution was more balanced, with 50.8% females compared with 59.6% in the FH-negative group (*P* < 0.01).

**Table 1:** Overview of subject characteristics from the Slovenian (*N* = 1360) and Portuguese (*N* = 360) registries used for the validation of established familial hypercholesterolemia diagnostic scores

Parameter	Category	SI: Overall	SI: FH positive	SI: FH negative	*P*-value^a^	PO: Overall	PO: FH positive	PO: FH negative	*P*-value^b^	*P*-value^c^
Genetic analysis	FH negative	1032 (75.9%)	0 (0.0%)	1032 (100.0%)	<0.01	172 (50.6%)	0 (0.0%)	172 (100.0%)	<0.01	<0.01
	FH positive	328 (24.1%)	328 (100.0%)	0 (0.0%)		168 (49.4%)	168 (100.0%)	0 (0.0%)		
Sex	Female	780 (57.4%)	169 (51.5%)	611 (59.2%)	0.02	190 (55.9%)	83 (49.4%)	107 (62.2%)	0.02	0.67
	Male	580 (42.7%)	159 (48.5%)	421 (40.8%)		150 (44.1%)	85 (50.6%)	65 (37.8%)		
Age [years]		6.3 (5.7–7.7)	6.1 (5.6–7.3)	6.4 (5.8–7.8)	<0.01	10.5 (8.0–13.0)	10.5 (8.0–13.0)	10.5 (8.8–13.0)	0.64	<0.01
High cholesterol in family	No	240 (17.7%)	31 (9.5%)	209 (20.3%)	<0.01	25 (7.4%)	5 (3.0%)	20 (11.6%)	0.01	<0.01
	Only first degree relative	267 (19.6%)	67 (20.4%)	200 (19.4%)		236 (69.4%)	116 (69.0%)	120 (69.8%)		
	Only second-degree relative	307 (22.6%)	47 (14.3%)	260 (25.2%)		15 (4.4%)	5 (3.0%)	10 (5.8%)		
	First and second-degree relative	546 (40.2%)	183 (55.8%)	363 (35.2%)		64 (18.8%)	42 (25.0%)	22 (12.8%)		
Family history of pre-mature coronary artery disease	No	1132 (83.2%)	250 (76.2%)	882 (85.5%)	0.01	276 (81.2%)	132 (78.6%)	144 (83.7%)	0.73	<0.01
	Only first degree relative	35 (2.6%)	9 (2.7%)	26 (2.5%)		33 (9.7%)	18 (10.7%)	15 (8.7%)		
	Only second-degree relative	190 (14.0%)	68 (20.7%)	122 (11.8%)		26 (7.7%)	15 (8.9%)	11 (6.4%)		
	First and second-degree relative	3 (0.2%)	1 (0.3%)	2 (0.2%)		5 (1.5%)	3 (1.8%)	2 (1.2%)		
Family history of pre-mature vascular disease (peripheral vascular disease, cerebrovascular insult)	No	1272 (93.5%)	302 (92.1%)	970 (94.0%)	0.43	328 (96.5%)	162 (96.4%)	166 (96.5%)	NA	0.21
	Only first degree relative	7 (0.5%)	3 (0.9%)	4 (0.4%)		2 (0.6%)	1 (0.6%)	1 (0.6%)		
	Only second-degree relative	79 (5.8%)	22 (6.7%)	57 (5.5%)		10 (2.9%)	5 (3.0%)	5 (2.9%)		
	First and second-degree relative	2 (0.2%)	1 (0.3%)	1 (0.1%)		0 (0.0%)	0 (0.0%)	0 (0.0%)		
Family history of tendinous xanthoma	No	1352 (99.4%)	323 (98.5%)	1029 (99.7%)	0.04	335 (98.5%)	166 (98.8%)	169 (98.3%)	1.0	0.22
	Yes	8 (0.6%)	5 (1.5%)	3 (0.3%)		5 (1.5%)	2 (1.2%)	3 (1.7%)		
Family history of arcus cornealis	No	1353 (99.5%)	323 (98.5%)	1030 (99.8%)	0.02	340 (100.0%)	168 (100.0%)	172 (100.0%)	NA	0.42
	Yes	7 (0.5%)	5 (1.5%)	2 (0.2%)		0 (0.0%)	0 (0.0%)	0 (0.0%)		
TC [mmol/L]		5.6 (5.1–6.3)	6.6 (6.0–7.3)	5.4 (5.0–5.9)	<0.01	7.0 (6.2–7.8)	7.6 (6.8–8.7)	6.4 (6.0–7.1)	<0.01	<0.01
TC [mg/dL]		216.6 (197.2–243.6)	255.2 (232.0–283.1)	208.8 (193.4–228.2)	<0.01	270.7 (239.8–301.7)	293.9 (263.0–336.4)	247.5 (232.0–274.6)	<0.01	<0.01
HDL-C [mmol/L]		1.5 (1.3–1.8)^d^	1.4 (1.2–1.6)	1.6 (1.3–1.8)	<0.01	1.4 (1.2–1.7)	1.3 (1.2–1.5)	1.5 (1.3–1.7)	<0.01	<0.01
HDL-C [mg/dL]		58.0 (50.3–69.6)^d^	54.1 (46.4–61.9)	61.9 (50.3–69.6)	<0.01	54.0 (47.0–64.0)	52.0 (44.8–59.0)	57.0 (49.0–66.3)	<0.01	<0.01
LDL-C [mmol/L]		3.6 (3.1–4.3)	4.8 (4.2–5.5)	3.4 (2.9–3.8)	<0.01	5.0 (4.4–5.9)	5.8 (5.0–6.7)	4.5 (4.0–5.0)	<0.01	<0.01
LDL-C [mg/dL]		139.2 (119.9–166.3)	185.6 (162.4–213.7)	131.5 (112.1–147.0)	<0.01	194.0 (170.0–228.3)	224.0 (194.8–258.5)	174.0 (156.0–194.0)	<0.01	<0.01
TAG [mmol/L]		0.9 (0.6–1.3)^e^	0.8 (0.6–1.2)	0.9 (0.6–1.3)	0.09	0.9 (0.7–1.3)	0.9 (0.7–1.3)	0.9 (0.7–1.3)	0.24	0.37
TAG [mg/dL]		79.7 (53.1–115.1)^e^	70.9 (56.5–106.3)	79.7 (53.1–115.1)	0.09	78.0 (62.0–112.0)	76.0 (60.0–112.0)	81.5 (63.8–112.0)	0.24	0.37
Lp(a) [mg/L]		128.5 (<99.4–420.0)^f^	107.0 (<99.4–343.0)	144.0 (<99.4–445.0)	0.03	83.0 (25.0–168.0)^g^	81.0 (22.0–168.0)	83.0 (32.0–168.3)	0.49	<0.01
BMI Z-Score		0.13 (−0.57–1.09)^h^	0.07 (−0.55–0.99)	0.16 (−0.57–1.11)	0.24	0.77 (−0.24–1.71)^i^	0.63 (−0.25–1.67)	0.85 (−0.20–1.73)	0.53	0.04

Data are absolute frequencies (proportions in %) and median (first-quartile–third-quartile). The χ^2^ test was used for comparison of categorical variables; Mann–Whitney test was used for comparison of numerical variables due to non-normal distribution. To control the false discovery rate, *P*-values were adjusted using the Benjamini–Hochberg method, with significance set at an adjusted *P* < 0.05.

SI, Slovenian registry; PO, Portuguese registry; FH, familial hypercholesterolemia; NA, Not available; TC, Total cholesterol; HDL-C, High-density lipoprotein cholesterol; LDL-C, Low-density lipoprotein cholesterol; TAG, Triglycerides; Lp(a), Lipoprotein(a); BMI, Body mass index.

^a^ *P*-value for comparisons between FH positive vs. FH negative within the Slovenian cohort.

^b^ *P*-value for comparisons between FH positive vs. FH negative within the Portuguese cohort.

^c^ *P*-value for comparisons between Slovenian and Portuguese cohort.

^d^Missing two values.

^e^Missing four values.

^f^Missing 140 values.

^g^Missing 23 values.

^h^Missing 29 values.

^i^Missing 25 values.

Family history in characteristics combined cohort differed significantly between FH-positive and FH-negative individuals. High cholesterol and pre-mature CAD were more common in FH-positive individuals (92.7% vs. 81.0% and 23.0% vs. 14.8%, respectively; *P* < 0.01), whereas pre-mature vascular disease showed no significant difference (6.5% vs. 6.6%; *P* = 0.68). High cholesterol in first-degree relatives was reported by 65.5% of participants. Conversely, family history of pre-mature CAD was less frequent, with only 4.5% reporting it in first-degree relatives. Tendinous xanthoma and arcus cornealis in relatives were rare and showed no significant difference between FH-positive and FH-negative individuals (1.4% vs. 0.5%, *P* = 0.11; and 1.0% vs. 0.2%, *P* = 0.05, respectively).

The median (Q1–Q3) LDL-C was 3.8 (3.2–4.7) mmol/L (147.0 (123.7–183.0) mg/dL). FH-positive subjects had significantly higher LDL-C concentrations compared with FH-negative subjects: 5.1 (4.4–6.0) mmol/L (196.0 (170.8–230.3) mg/dL) vs. 3.5 (3.0–4.0) mmol/L (135.4 (116.0–156.0) mg/dL), respectively (*P* < 0.01). Clinical manifestations of FH were rare; no subjects had pre-mature CVD, and only two FH-positive individuals presented with tendinous xanthoma (in the PO cohort).

The characteristics for the SI and PO cohorts are presented in *Table 1* and demonstrate several relevant differences. The proportion of FH-positive cases is significantly higher in the PO cohort (+25.3%; *P* < 0.01). Children in the PO cohort are also older on average, with a mean age of 10.5 years compared with 6.3 years in the SI cohort (*P* < 0.01). Notably, family history of hypercholesterolemia differs significantly, with a higher prevalence of negative family history cases in the SI cohort (17.7% vs. 7.4%, *P* < 0.001). Moreover, total cholesterol concentrations are markedly higher in the PO cohort (7.0 vs. 5.6 mmol/L [270.3 vs. 216.4 mg/dL], *P* < 0.01), as are LDL-C concentrations (5.0 vs. 3.6 mmol/L [193.5 vs. 139.3 mg/dL], *P* < 0.01), whereas Lp(a) concentrations are lower (83.0 vs. 128.5 mg/L). Additionally, the BMI Z-score is slightly higher in the PO cohort (*P* = 0.04).

### Scores validation

The ROC analysis for the DLCN criteria demonstrated no statistically significant differences in AUCs between the two cohorts (see Supplementary material online, *Figure S4*). The overall AUC was 0.857, while the SI and PO had AUCs of 0.852 and 0.815, respectively. Pairwise comparisons showed no significant differences between the Combined and SI cohort AUCs (*P* = 0.73), the Combined and PO cohort AUCs (*P* = 0.08), or the SI and PO cohort AUCs (*P* = 0.14), indicating relatively consistent performance of the criteria across registries.

**Figure 2:**
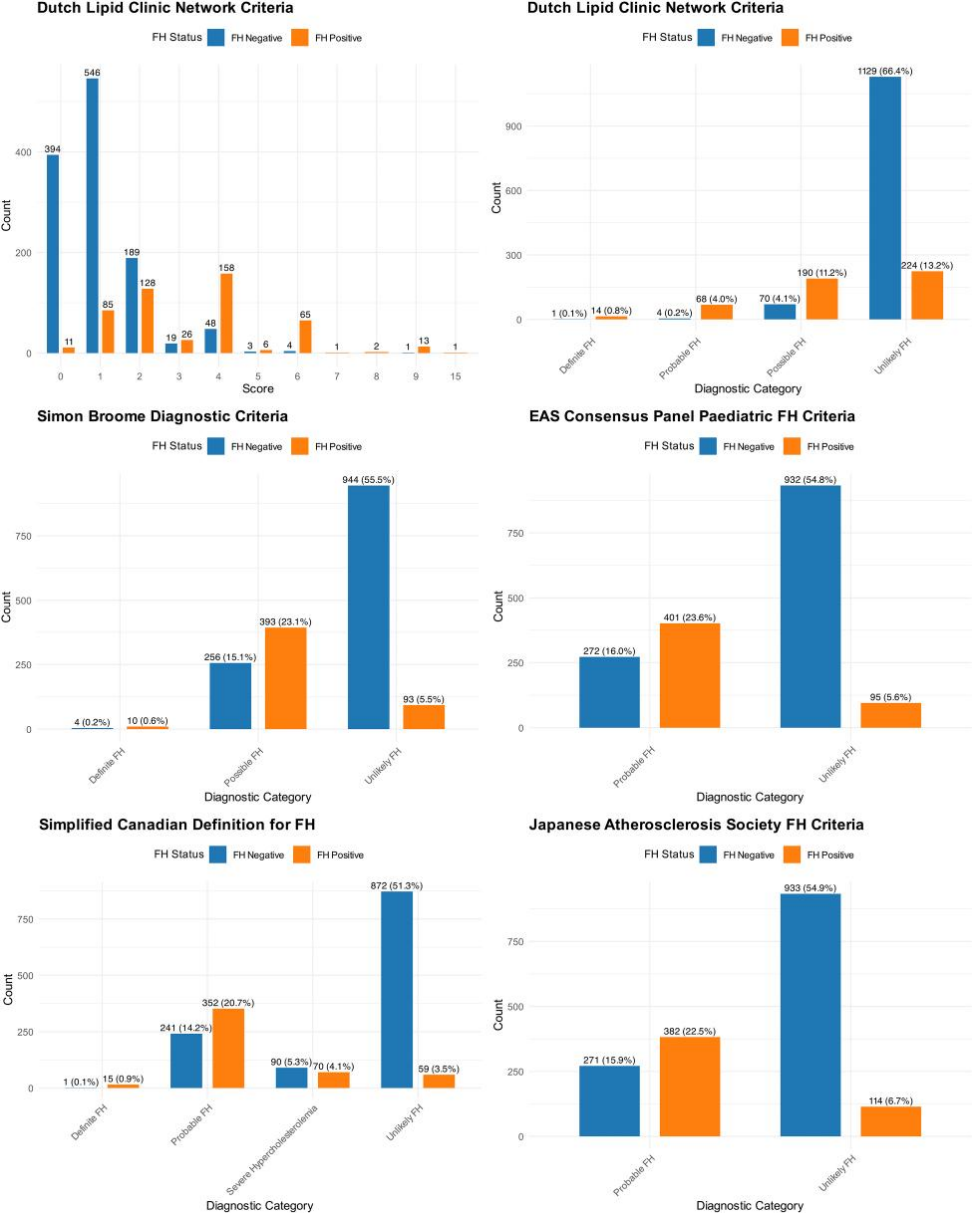
Bar plots of diagnostic criteria for familial hypercholesterolemia (FH) in combined cohort.

The DLCN exhibited excellent specificity already at relatively low scores (see *Figure 2*). Specificity exceeded 90% for scores ≥3 (93.8%) and reached 99.9% at scores ≥7. However, at a score ≥3, sensitivity was 54.8%, declining to 17.7% at ≥5 and 3.4% at ≥7. PPV was 78.4% at ≥3, 91.7% at ≥5, and 94.4% at ≥7. NPV at these thresholds ranged from 71.5% at ≥7 to 83.4% at ≥3 and reached 90.7% at the score ≥2. Lower thresholds showed improved sensitivity but reduced specificity.

Supplementary material online, *Figure S3* presents the performance metrics of various FH diagnostic criteria, converted into binary classifications (FH-positive vs. FH-negative) for comparison. EAS and SB had the highest sensitivity (80.8% and 81.2%, respectively), while DLCN showed the lowest sensitivity (54.8%). However, DLCN had the highest specificity (93.8%), outperforming other criteria, which ranged from 77.5% to 80.8%. The best balance between sensitivity and specificity, based on Youden’s index, was achieved by SB (59.7%), followed closely by EAS (58.5%). A similar trade-off was observed in PPV and NPV—DLCN had a significantly higher PPV (78.4%) compared with other criteria (56.6–61.7%) but a lower NPV (83.4%) relative to others (88.9–91.0%). Accuracy varied only slightly across criteria, ranging from 77.4% (JAP) to 84.1% (DLCN). Cohen’s kappa index showed no clear superiority, with values spanning from 48.1% (JAP) to 54.3% (SB).

When comparing metrics between the SI and PO cohorts, significant differences were observed across all criteria. Sensitivity was consistently higher in the PO cohort, with the largest differences for DLCN (+35.0%), CAN (+31.8%), SB (+24.7%), JAP (+27.5%), and EAS (+23.1%) (all *P* < 0.01). In contrast, specificity was significantly higher in the SI cohort, particularly for SB (+60.2%), JAP (+68.2%), CAN (+54.0%), and EAS (+57.8%) (all *P* < 0.01). PPV differences were smaller but favoured the SI cohort for DLCN (+8.9%) and JAP (+8.4%; *P* = 0.02), while NPV showed variability—JAP performed better in the SI cohort (+9.1%; *P* < 0.01), whereas other criteria showed minimal differences. Accuracy and kappa also reflected cohort differences: the SI cohort had higher accuracy for DLCN (84.1% vs. 75.6%) and SB (91.0% vs. 78.4%), while EAS slightly favoured the PO cohort (83.5% vs. 82.6%). Similarly, kappa was higher in the SI cohort for DLCN (53.3% vs. 48.1%) and SB (54.3% vs. 50.5%), but EAS showed marginally better agreement in the PO cohort (55.1% vs. 52.7%). Youden’s index favoured the SI cohort for DLCN (+24.3%) and SB (+35.0%), while EAS performed slightly better in the PO cohort (+2.4%).

All clinical scores collectively diagnosed 47.4% of genetically confirmed FH-positive cases, while 10.9% were missed by every clinical criterion. More on overlap analysis can be found in the Supplementary Materials.

### Development of the new clinical diagnostic score (FH-PeDS)

**Figure 3:**
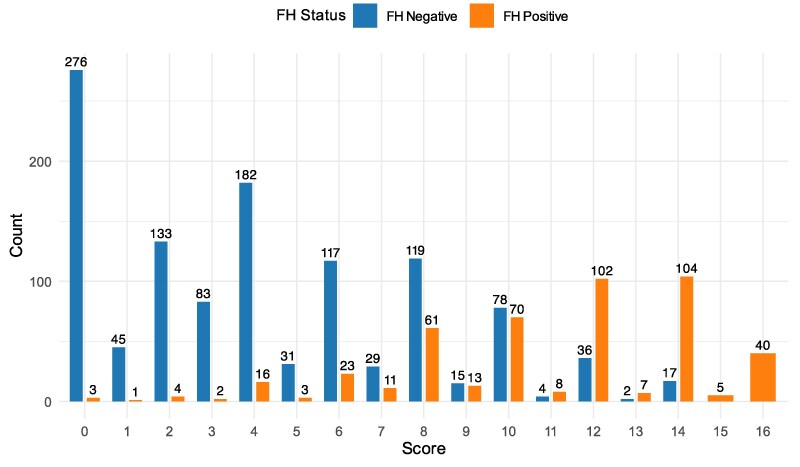
Distribution of FH paediatric score (FH-PeDS) in subjects with and without familial hypercholesterolemia in the combined cohort.

We developed a semi-quantitative FH Paediatric Score **(FH-PeDS)** using a multi-step approach to convert numerical variables into clinically relevant categories with distinct cut-offs. Variables used included age, sex, family history (high cholesterol, pre-mature CV disease, tendinous xanthoma, or arcus cornealis), HDL-C, LDL-C, TAG, and BMI Z-score. *Table 2* displays the score calculation, while *Figure 3* illustrates the distribution of scores in the combined cohort. In the SI cohort, the new score outperformed the DLCN score with an AUC of 0.906 vs. 0.852 (*P* < 0.01). In the combined cohort, **FH-PeDS** maintained its superior performance, yielding an AUC of 0.897 compared with 0.857 for DLCN (*P* < 0.01). Although overall performance in the PO cohort was slightly lower, **FH-PeDS** still demonstrated an advantage with an AUC of 0.830 vs. 0.811 for DLCN (*P* = 0.329).

**Table 2:** Familial hypercholesterolemia paediatric diagnostic score (FH-PeDS)

Points	Criteria	
LDL-C^a^		
14	LDL-C > 6.5 mmol/L (250 mg/dL)	
12	4.8 mmol/L (185 mg/dL) < LDL-C ≤ 6.5 mmol/L (250 mg/dL)	
8	3.8 mmol/L (145 mg/dL) < LDL-C ≤ 4.8 mmol/L (185 mg/dL)	
4	3.0 mmol/L (115.0 mg/dL) < LDL-C ≤ 3.8 mmol/L (145 mg/dL)	
HDL-C^a^		
−2	1.4 mmol/L (55 mg/dL) < HDL-C ≤ 2.2 mmol/L (85 mg/dL)	
−4	HDL-C > 2.2 mmol/L (85 mg/dL)	
TAG^a^		
−2	2.0 mmol/L (175 mg/dL) < TAG ≤ 3.5 mmol/L (310 mg/dL)	
−4	3.5 mmol/L (310 mg/dL) < TAG ≤ 4.5 mmol/L (400 mg/dL)	
−6	TAG > 4.5 mmol/L (400 mg/dL)	
BMI		
−2	BMI Z-score > 1.645 (BMI > 95th percentile^b^)	
Family History^c^		
4	First-degree relative with arcus cornealis <45 years/tendon xanthomas or first-degree relative with genetically confirmed FH^c^	
2	First-degree relative with pre-mature coronary artery disease^d^ or first-degree relative with high cholesterol^c^	
2	Second-degree relative with arcus cornealis <45 years/tendon xanthomas^c^	
1	Second-degree relative with pre-mature coronary artery disease^d^ or Second-degree relative with high cholesterol^c^	
**FH-PeDS** <6: Unlikely FH	6≤ **FH-PeDS** <9: Possible FH (follow-up required)	**FH-PeDS** ≥9: Probable FH

FH-PeDS version: 1.0.

LDL-C, low-density lipoprotein cholesterol; HDL-C, high-density lipoprotein cholesterol; TAG, triglycerides; BMI, body mass index; FH, familial hypercholesterolemia.

^a^Untreated concentration.

^b^The 95th percentile serves as the threshold for diagnosing obesity.

^c^One can receive points for only one of these categories, not all.

^d^Coronary artery disease is considered pre-mature if it occurs in men under 55 years or women under 60 years.

Using Youden’s index to determine optimal cut-offs in the combined cohort, DLCN performed best at a threshold of 2 (Youden 0.588, sensitivity 80.3%, specificity 78.5%), while **FH-PeDS** peaked at 8 (Youden 0.635, sensitivity 86.7%, specificity 76.8%). In the SI cohort, DLCN’s optimal threshold was 2 (Youden 0.584, sensitivity 72.2%, specificity 86.2%), whereas **FH-PeDS** performed best at 7 (Youden 0.665, sensitivity 84.8%, specificity 81.7%). In the PO cohort, DLCN performed best at threshold 4 (Youden 0.543, sensitivity 78.3%, specificity 76.0%), while **FH-PeDS** peaked at 12 (Youden 0.549, sensitivity 78.3%, specificity 76.6%).

For the SI cohort, a threshold of 4 (**FH-PeDS** ≥4) yielded 96.8% sensitivity, 52.7% specificity, and a PPV of 39.1%, while the threshold of 6 yielded 91.1% sensitivity, 72.0% specificity, and a PPV of 50.4%. A threshold of 9 resulted in 92.9% specificity, 64.6% sensitivity, and a PPV of 73.9%. At threshold 13, specificity reached 99.5%, with 26.3% sensitivity and a PPV of 94.3%. More details on sensitivity, specificity, and PPV for different cut-offs across cohorts can be found in Supplementary material online, *Table S7*.

### Development of the new diagnostic score using a machine learning model (ML-FH-PeDS)

#### Overview of subjects’ characteristics

Individuals with hypercholesterolemia from the SI Registry were randomly assigned in a 60%/40% ratio to a training cohort and a testing cohort. When allocating participants, we ensured that the proportion of genetically confirmed FH cases was equal in both cohorts. External validation was performed on the dataset from the PO Registry. The training and testing cohorts had a similar proportion of FH-positive individuals (190, 23.9% vs. 126, 23.8%) and were also comparable in other characteristics, such as sex distribution, median age, family history, and lipid profile parameters (descriptive statistics for both cohorts are presented in Supplementary material online, *Table S8*).

#### Performance of the machine learning model (ML-FH-PeDS)

**Figure 4:**
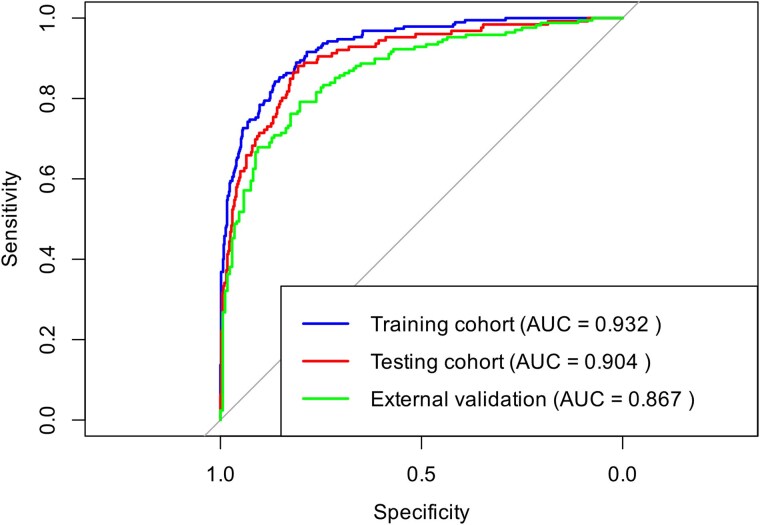
Receiver operating characteristic (ROC) curve of the machine learning model for familial hypercholesterolemia (FH) diagnosis. The training and testing data are derived from the Slovenian registry, while the external validation corresponds to the Portuguese cohort.

The **ML-FH-PeDS** demonstrated high predictive power for detecting individuals with genetically confirmed FH, with similar performance in both the training and testing cohort (AUC = 0.932 and AUC = 0.904, respectively). The slight drop in performance in the external validation cohort suggests that population-specific differences may affect the model's predictive accuracy (*Figure 4*). Despite this, the AUC of 0.867 indicates that the model remains robust and reliable for detecting cases with genetically confirmed FH across different populations.

At the 0.5 threshold, the model achieved high specificity (94.8%), sensitivity (61.1%), and a PPV of 78.6% in the testing cohort. However, these metrics differed significantly in the PO cohort, where sensitivity was substantially higher (92.3%), while specificity dropped to 55.2%, with a comparable PPV. Since diagnostic scores are primarily used as confirmatory tests, we further assessed the model’s diagnostic performance at various specificity concentrations. As shown in Supplementary material online, *Table S9*, increasing specificity results in a corresponding reduction in sensitivity in the testing cohort. Notably, the model appears to perform best as a confirmatory test at a specificity of 98%, achieving a sensitivity of 39.7% and a PPV of 87.7%.

The strongest positive parameter weights (see Supplementary material online, *Table S10*) in our model for predicting FH were associated with both LDL-C and TC, indicating that higher concentrations of LDL-C and TC increase the likelihood of a positive FH diagnosis. On the other hand, the highest negative weights were associated with HDL-C, TAG, and Lp(a), indicating that higher values of these parameters correspond to a lower probability of an FH diagnosis. Age and BMI Z-score had an insignificant contribution to the model, suggesting that these variables do not strongly influence the prediction of FH in our dataset. Family history had a mixed contribution to the model. While the highest positive parameter weight was attributed to a history of high cholesterol in both first- and second-degree relatives, negative family history of high cholesterol was associated with the most negative weight, indicating a reduced likelihood of FH diagnosis in such cases.

## Discussion

In this study, we aimed to evaluate the clinical utility of five established FH diagnostic criteria, originally developed for adults, as tools for identifying children with some or all clinical features of FH. Additionally, we developed two new FH diagnostic scores for children—**FH-PeDS** and **ML-FH-PeDS**.

Existing FH diagnostic scores incorporate cholesterol concentrations, clinical signs of cholesterol deposits, and personal and family history of pre-mature CV disease and hypercholesterolemia.^19,20,31,33^ However, their applicability in paediatrics is limited. A negative family history does not exclude FH in children, as their parents may be too young for ASCVD to manifest despite carrying the condition.^37^ Moreover, most diagnostic systems were developed and validated in adults and rely on clinical signs, such as tendon xanthoma, and pre-mature CV events that are typically absent in paediatric heterozygous FH patients.^24,38,39^ This was evident in our study, where only two children had tendinous xanthoma, and none in the SI cohort had a personal history of CVD. Additionally, family history is often incomplete or unreliable in real-world practice.^37^ Many of these scores were established decades ago, before modern CV prevention strategies, and fail to account for the early, subclinical presentation of FH in children.^19,20^ These limitations underscore the urgent need for paediatric-specific diagnostic tools.

The key findings of this study indicate that among the tested diagnostic algorithms, the DLCN criteria performed best, though with a clear trade-off between sensitivity and specificity depending on the chosen threshold. Higher DLCN scores (≥7) provided excellent specificity (up to 99.9%) and PPV (94.4%), effectively confirming FH when positive, but with very low sensitivity (as low as 3.4%), meaning most true cases were missed. Lower thresholds (≥2) improved sensitivity (up to 80.6%) but reduced specificity (78.1%) and PPV (60.2%). Significant differences were observed between the SI and PO cohorts, with the PO cohort showing higher sensitivity, while the SI cohort, derived from a universal screening approach, exhibited greater specificity and overall accuracy. These differences likely stem from Portugal's lack of a universal FH screening programme for children, meaning the Registry primarily includes individuals with markedly elevated cholesterol concentrations, leading to a higher proportion of FH cases.^30^ Additionally, the PO cohort is older, has a stronger family history of FH, and presents with higher TC and LDL-C concentrations, further influencing the observed diagnostic performance.

Variability in concordance among different diagnostic criteria was also observed. While criteria with similar scoring systems showed high agreement, 10.9% of genetically confirmed FH-positive individuals were missed by all clinical criteria, highlighting substantial limitations. These findings underscore the inherent constraints of current FH diagnostic scores and the need for improved, population-specific, or alternative approaches, such as machine learning–based strategies, to enhance early and accurate detection.

The second key finding is that the newly developed **FH-PeDS** diagnostic algorithm consistently outperformed the DLCN score across both cohorts. Identified thresholds in the SI cohort—6, 9, and 13—offer practical guidance: individuals scoring below 6 are unlikely to have FH and may not require genetic testing, while those scoring above 9 have a high likelihood of FH. At a threshold of 13, FH is nearly certain, with a specificity of 99.5%, indicating definite FH. However, genetic testing remains recommended when available to confirm the diagnosis and facilitate cascade screening of at-risk relatives.^9^ Importantly, threshold selection for genetic testing or clinical diagnosis should account for population-specific factors, including recruitment methods and diagnostic criteria.

**ML-FH-PeDS** offers a flexible diagnostic approach, allowing threshold adjustments to balance sensitivity and specificity based on clinical needs. At very low thresholds, nearly all individuals are flagged as positive, maximizing sensitivity but lowering specificity and precision. As thresholds increase, specificity improves while sensitivity declines. In the SI cohort, sensitivity approached 100% at the lowest thresholds, while at a threshold of 0.5, specificity reached 94.8% with a PPV of 78.6%. In the PO cohort, at the same threshold, sensitivity was 92.3% and specificity 55.2%, highlighting how population-specific factors influence performance. Unlike fixed-threshold scoring systems such as DLCN, **ML-FH-PeDS** allows threshold customization, making it adaptable for both broad screening and confirmatory settings. With high AUC values in both internal and external validation, the model improves early detection while offering tailored diagnostic performance across diverse populations, addressing key limitations of traditional criteria. While **FH-PeDS** and **ML-FH-PeDS** were developed similarly, converting **FH-PeDS** into a semi-quantitative grading system slightly reduces predictive power but enhances interpretability and usability, particularly in resource-limited settings. Both models leverage real-world clinical data, overcoming limitations seen in many existing diagnostic scoring systems.

Logistic regression analysis, controlling for all other factors revealed that boys face a higher risk of FH, a finding corroborated by feature weights from the **ML-FH-PeDS** model. Although the underlying mechanisms remain unclear, one possible explanation is that boys typically have lower LDL-C concentrations than girls, despite a similar FH prevalence between the sexes.

Several studies have successfully applied machine learning to FH diagnosis in adults, often surpassing traditional LDL-C cut-offs and matching established clinical criteria. However, these models have been primarily adult-focused.^40–42^ In contrast, our study is the first to develop and validate **ML-FH-PeDS** specifically for children, identified through a universal screening programme, offering a promising tool for early detection and intervention in the general paediatric population.

The **FH-PeDS** and **ML-FH-PeDS** tools are designed primarily for use in universal or opportunistic paediatric screening settings, particularly when family history data are limited or genetic testing capacity is constrained. In such contexts, these tools can help clinicians identify children with a high likelihood of FH who should be referred for genetic testing and further evaluation. Once confirmed, these children can serve as pro-bands for initiating family based cascade screening, including reverse cascade approaches. However, these models are not intended for phenotypic cascade testing of children who already have a known first-degree relative with FH; in such cases, age- and sex-specific LDL-C thresholds (e.g. >3.5 mmol/L) remain the most appropriate and efficient diagnostic strategy.^12^ Our approach thus complements existing cascade testing protocols by focusing on pro-bands identified through population-based or routine clinical encounters, enabling earlier detection and broader family outreach.

A key advantage of an efficient diagnostic algorithm is its ability to better identify individuals most likely to have true monogenic FH, guiding confirmatory genetic testing. In low- and middle-income countries and constrained healthcare systems, genetic testing is often impractical due to high costs, limited access, and significant delays.^43–45^ Thus, a cost-effective clinical scoring tool is essential for stratifying patients and prioritizing genetic testing.^46^ This is particularly important as individuals with FH face a substantially higher CV risk at the same cholesterol concentrations compared with non-FH individuals.^47,48^ By efficiently identifying high-risk cases using an inexpensive, easy-to-use tool that requires no specialized genetic expertise, healthcare systems can increase FH diagnoses while optimizing resource allocation for confirmatory testing.^49,50^

Given these findings, further validation in globally diverse, non-European populations will be important to confirm generalizability across healthcare settings. It will also be important to assess performance in other structured, population-based screening programmes, such as the VRONI study in Germany, which represents a well-organized cohort for early FH detection in a general paediatric population and could serve as a valuable setting for future external validation of the proposed models.^51^

To facilitate real-world use, the next step in developing the proposed diagnostic tools involves creating user-friendly digital formats—such as online calculators or integration into electronic health records—that allow easy application by clinicians in both primary and specialist care. Prospective studies will be necessary to assess their diagnostic performance, usability, and clinical impact in real-time settings. Additionally, implementation research should explore optimal pathways for incorporating these tools into existing paediatric lipid screening strategies, especially in regions where genetic testing access is limited. These efforts will support broader adoption and help translate our findings into tangible improvements in early FH detection and care.

Our study has several limitations. First, both the development and validation cohorts were derived from European populations, which may limit generalizability to other ancestry groups and healthcare settings. While the Slovenian and Portuguese cohorts differ in ancestry, age distribution, lipid profiles, and referral context, this does not substitute for validation in non-European, population-based cohorts. Broader validation in ancestrally diverse populations—particularly in Asia, South Asia, Latin America, and Africa—is needed to assess the portability of both FH-PeDS and ML-FH-PeDS. These efforts are currently underway through newly established international collaborations, and we view this study as a foundational step in that process. Second, the external validation cohort from Portugal consisted of older children, and its referral-based nature resulted in a higher pre-test probability of FH, which may limit generalizability to screening-based settings. Third, our study population did not include children younger than 5 years, as the Slovenian universal screening programme targets children aged 5 and above. The applicability of both diagnostic tools in younger age groups, where lipid variability and incomplete family history may present additional challenges, remains to be explored. Fourth, selection bias may have arisen from excluding cases with missing data or VUS. Fifth, the retrospective design and evolving genetic testing methods could affect diagnostic consistency. Sixth, relying on real-world clinical data limits the application of existing diagnostic scoring systems, particularly regarding family history. Seventh, **FH-PeDS** was designed for universal or opportunistic paediatric FH screening, rather than for cascade testing in at-risk families, where several dedicated scores already exist.^19,20,22^ Based on preliminary validation (see Supplementary Materials) and clinical experience, we allocated points for a first-degree relative with genetically confirmed FH, though the limited cascade cases in the SI registry prevented full validation, necessitating further assessment in larger cascade cohorts. However, as suggested by Nordestgaard *et al.*, in children with an affected parent, an LDL-C concentration >3.5 mmol/L (>135 mg/dL) strongly indicates FH and may serve as a simpler alternative to complex scoring strategies for cascade testing.^12^ Finally, larger and more diverse cohorts are needed for further validation and refinement of the models.

## Conclusion

This study demonstrates that the currently available clinical diagnostic scores for FH have limited applicability in children. To address this gap, we developed the FH Diagnostic Score (**FH-PeDS**), which offers a more accurate and practical approach to clinical diagnosis, particularly in settings where genetic testing is either inaccessible or cost-prohibitive. Additionally, we developed the **ML-FH-PeDS** that achieves high predictive power and provides flexibility to tailor performance to diverse clinical needs; this model is planned to be freely available as an online tool.

**FH-PeDS Collaborators:** Barbara Cugalj Kern (Faculty of Medicine, University of Ljubljana, Slovenia; Clinical Institute of Special Laboratory Diagnostics, University Children's Hospital, University Medical Centre Ljubljana, Slovenia), Jernej Kovač (Faculty of Medicine, University of Ljubljana, Slovenia; Clinical Institute of Special Laboratory Diagnostics, University Children's Hospital, University Medical Centre Ljubljana, Slovenia), Ana Drole Torkar (Faculty of Medicine, University of Ljubljana, Slovenia; Department of Endocrinology, Diabetes, and Metabolic Diseases, University Children's Hospital, University Medical Centre Ljubljana, Slovenia), Maja Filipič (Faculty of Medicine, University of Ljubljana, Slovenia; Department of Endocrinology, Diabetes, and Metabolic Diseases, University Children's Hospital, University Medical Centre Ljubljana, Slovenia), Mia Becker (Community Healthcare Center Dr Adolf Drolc Maribor, Slovenia; Faculty of Medicine, University of Maribor, Slovenia), Žiga Iztok Remec (Clinical Institute of Special Laboratory Diagnostics, University Children's Hospital, University Medical Centre Ljubljana, Slovenia), Barbka Repič Lampret (Clinical Institute of Special Laboratory Diagnostics, University Children's Hospital, University Medical Centre Ljubljana, Slovenia), Maruša Debeljak (Faculty of Medicine, University of Ljubljana, Slovenia; Clinical Institute of Special Laboratory Diagnostics, University Children's Hospital, University Medical Centre Ljubljana, Slovenia), Katarina Trebušak Podkrajšek (Clinical Institute of Special Laboratory Diagnostics, University Children's Hospital, University Medical Centre Ljubljana, Slovenia; Institute of Biochemistry and Molecular Genetics, Faculty of Medicine, University of Ljubljana, Slovenia), Zlatko Fras (Faculty of Medicine, University of Ljubljana, Slovenia; Centre for Preventive Cardiology, Division of Medicine, University Medical Centre Ljubljana, Slovenia), Borut Jug (Faculty of Medicine, University of Ljubljana, Slovenia; Department of Vascular Diseases, Division of Internal Medicine, University Medical Centre Ljubljana, Slovenia), Fouzia Sadiq (Directorate of Research, Shifa Tameer-e-Millat University, Islamabad, Pakistan), António Guerra (Department of Pediatrics, Hospital São João, Centro Hospitalar Universitário São João, Portugal), Ana Gaspar (Department of Pediatrics, Unidade de Doenças Metabólicas, Hospital de Santa Maria, Centro Hospitalar de Lisboa Norte, Portugal), Henedina Antunes (Department of Pediatrics, Hospital de Braga, Portugal), Sílvia Sequeira (Centro de Referência de Doenças Hereditárias do Metabolismo, Hospital de Dona Estefânia, Centro Hospitalar Universitário de Lisboa Central, Portugal), Susana Correia (Hospital de Nossa Senhora do Rosário, Centro Hospitalar Barreiro Montijo, Portugal), Paula Garcia (Centro de Referência de Doenças Hereditárias do Metabolismo, Hospital Pediátrico, Centro Hospitalar Universitário de Coimbra, Portugal), Luísa Diogo Matos (Department of Pediatrics, Hospital Sta Maria Maior, Portugal), Goreti Lobarinhas (Department of Pediatrics, Hospital Sta Maria Maior, Portugal), Paula Martins (Department of Pediatric Cardiology, Hospital Pediátrico, Centro Hospitalar Universitário de Coimbra, Portugal), Guida Gama (Department of Pediatrics, Centro Hospitalar do Algarve, Portugal), Mónica Tavares (Department of Pediatrics, Centro Materno Infantil do Norte, Centro Hospitalar e Universitário de Sto António, Portugal), Eduard Ostarijas (Doctoral School of Clinical Medical Sciences, Medical School, University of Pécs, Hungary), Zala Jelinčič (Faculty of Medicine, University of Ljubljana, Slovenia), Dimitar Trifunoski (Faculty of Medicine, University of Ljubljana, Slovenia), Luka Pesjak (Faculty of Medicine, University of Ljubljana, Slovenia).

## Supplementary Material

zwaf352_Supplementary_Data

## Data Availability

Data is available from the corresponding author upon reasonable request.

## Code availability

The analytical code used in this study can be shared upon reasonable request from the corresponding author.

## Study protocol and registration

No formal study protocol was prepared for this research. Additionally, the study was not registered in a public trial registry, as it did not meet the criteria for mandatory registration of observational studies.

## Patient and public involvement

Patients and the public were not involved in the design, conduct, reporting, interpretation, or dissemination of this study.

## Originality of content

We confirm all information and materials in the manuscript are original.
