# Supplementary material for: Proposal of a familial hypercholesterolemia paediatric diagnostic score (FH-PeDS)
Source: Eur J Prev Cardiol. 2025 Jun 20;33(9):1572–84. doi: 10.1093/eurjpc/zwaf352 (PMC13364047; doi:10.1093/eurjpc/zwaf352)
Supplement: zwaf352_Supplementary_Data [file zwaf352_supplementary_data.docx]

**Proposal of a Familial Hypercholesterolemia Pediatric Diagnostic Score (FH-PeDS): Supplementary materials**

Table of Contents

[Genetic analysis 3](#_Toc191387090)

[Calculation of Established FH Scores 4](#_Toc191387091)

[Table 1: Simon Broome Diagnostic criteria. 4](#_Toc191387092)

[Table 2: EAS Consensus Panel Pediatric FH Criteria. 5](#_Toc191387093)

[Table 3: Dutch Lipid Clinic Network criteria. 6](#_Toc191387094)

[Table 4: Simplified Canadian Definition for FH. 7](#_Toc191387095)

[Table 5: Japanese Atherosclerosis Society FH criteria (under the age of 15). 8](#_Toc191387096)

[Table 6: Japanese Atherosclerosis Society FH criteria (15 years of age or older). 9](#_Toc191387097)

[Established FH Scores Overlap Analysis 11](#_Toc191387098)

[Preliminary Validation of Additional Points in FH-PeDS for a Genetically Positive First-Degree Relative 12](#_Toc191387099)

[Table 7: Sensitivity, Specificity, Positive Predictive Value (PPV) and Negative Predictive Value (NPV) for different cut-offs of the New Clinical FH Score (FH-PeDS). 13](#_Toc191387100)

[Table 8: Training cohort and testing cohort overview of characteristics. 14](#_Toc191387101)

[Table 9: Performance metrics of the machine learning model at different threshold levels. 17](#_Toc191387102)

[Table 10: Parameter weights in machine learning model (ML-FH-PeDS) for predicting familial hypercholesterolemia (FH). 20](#_Toc191387103)

[Figure 1: Bar Plots of Diagnostic Criteria for Familial Hypercholesterolemia (FH) in Slovenian Registry. 21](#_Toc191387104)

[Figure 2: Bar Plots of Diagnostic Criteria for Familial Hypercholesterolemia (FH) in Portuguese Registry. 22](#_Toc191387105)

[Figure 3: Performance Metrics of Diagnostic Criteria for Familial Hypercholesterolemia (FH). Note 23](#_Toc191387106)

[Figure 4: Receiver Operating Characteristic Curves of the Dutch Lipid Clinic Network Criteria for Familial Hypercholesterolemia Across Combined (Overall), Slovenian, and Portuguese Registries. 24](#_Toc191387107)

[Figure 5: Overlap between Diagnostic Criteria for Familial Hypercholesterolemia and Genetically Confirmed Familial Hypercholesterolemia (Combined Registry) 25](#_Toc191387108)

[Figure 6: Overlap between Diagnostic Criteria for Familial Hypercholesterolemia and Genetically Confirmed Familial Hypercholesterolemia (Slovenian Registry). 26](#_Toc191387109)

[Figure 7: Overlap between Diagnostic Criteria for Familial Hypercholesterolemia and Genetically Confirmed Familial Hypercholesterolemia (Portuguese Registry). 27](#_Toc191387110)

[Figure 8: Receiver Operating Characteristic (ROC) Curves Comparing the Dutch Lipid Clinic Network Score (DLCN) and the New FH Score for Familial Hypercholesterolemia Diagnosis (FH-PeDS) in the Combined Cohort. 28](#_Toc191387111)

[Figure 9: Receiver Operating Characteristic (ROC) Curves Comparing the Dutch Lipid Clinic Network Score (DLCN) and the New FH Score for Familial Hypercholesterolemia Diagnosis (FH-PeDS) in the Slovenian Cohort. 29](#_Toc191387112)

[Figure 10: Receiver Operating Characteristic (ROC) Curves Comparing the Dutch Lipid Clinic Network Score (DLCNS) and the New FH Score for Familial Hypercholesterolemia Diagnosis (FH-PeDS) in the Portuguese Cohort. 30](#_Toc191387113)

[Figure 11: Distribution of FH Pediatric Score (FH-PeDS) in Subjects with and Without Familial Hypercholesterolemia in the Slovenian Cohort. 31](#_Toc191387114)

[Figure 12: Distribution of FH Pediatric Score (FH-PeDS) in Subjects with and Without Familial Hypercholesterolemia in the Portuguese Cohort. 32](#_Toc191387115)

[References 33](#_Toc191387116)

# Genetic analysis

**Slovenian Cohort**

Genetic analyses were conducted at the Clinical Institute of Special Laboratory Diagnostics, University Children's Hospital, University Medical Centre Ljubljana. Over the years, advancements in DNA sequencing methodologies led to the use of various approaches to detect FH-associated genes.

Initially, Sanger sequencing was employed to analyze all exons of the *LDLR* gene and part of exon 26 of the *APOB* gene. This was later replaced by next-generation sequencing (NGS) using the ADH MASTR 2 commercial kit (Multiplicom, Belgium), which targeted coding and promoter regions of the *APOE*, *LDLR*, and *PCSK9* genes, as well as part of exon 26 of the *APOB* gene.

Subsequently, NGS technology evolved to the use of xGen Lockdown probes (IDT, USA), enabling the detection of coding and promoter regions of 18 genes associated with dyslipidemias (*ABCA1, ABCG5, ABCG8, ALMS1, APOA1, APOA5, APOB, APOC2, APOC3, APOE, CREB3L3, GPIHBP1, LDLR, LDLRAP1, LIPA, LMF1, LPL, PCSK9*). This gene panel was designed based on a commercially established hyperlipidemia panel. More recently, routine diagnostics have transitioned to whole exome sequencing, with patients analyzed using the Illumina DNA Prep with enrichment (Illumina, USA) and xGen Exome Research Panel v2 probes (IDT, USA).

NGS libraries were sequenced on MiSeq or NovaSeq 6000 platforms using MiSeq or NovaSeq Reagent Kits (Illumina, USA), following the manufacturer’s protocols. Variants in FH-associated genes were filtered using the VarAFT tool (1). Novel variants not previously reported in the literature were assessed using in silico prediction algorithms such as MutationTaster, PolyPhen2, SIFT, CADD, REVEL, and Human Splicing Finder (2-7). All identified variants were reclassified in accordance with the latest American College of Medical Genetics and Genomics (ACMG) guidelines and Clinical Genome Resource (ClinGen) expert panel recommendations as (likely) benign, variant of uncertain significance (VUS), or (likely) pathogenic (8, 9). Clinically relevant variants were confirmed by targeted Sanger sequencing and validated using Variant Validator (10). Variants not previously described in the literature or included in the ClinVar database were identified and annotated (11).

**Portuguese cohort**

Between 1999 and 2017, the molecular study of EPHF participants was conducted using Sanger sequencing to analyze all exons of the *LDLR* and *PCSK9* genes, and two fragments of *APOB* gene (complete exons 26 and 29). Since 2017, the molecular study is performed using an extended targeted NGS panel including eight genes: FH primary genes (*LDLR, APOB, and PCSK9*) and five FH-phenocopy genes (*LDLRAP1, APOE, LIPA, ABCG5, and ABCG8*). Samples are prepared according to SureSelect QXT Target Enrichment System protocol (Agilent Technologies) and run in a NextSeq platform (Illumina) using a NextSeq 550 System. Resulting files are analyzed using the SureCall software (Agilent Technologies), wANNOVAR software and DECoN-v1.0.2 software (for copy-number variant identification). Rare variants in FH genes are confirmed by Sanger sequencing and multiplex ligation-dependent probe amplification (MLPA) for copy-number variants in *LDLR* gene. Genetic variants are classified as (likely) benign, VUS or (likely) pathogenic according to ACMG recommendations (8). *APOB* and PCSK9 variants are classified according to ACMG guidelines using specific published adaptations (12), and LDLR variants are classified according to ClinGen expert panel recommendations (9).

Calculation of Established FH Scores

Table 1: Simon Broome Diagnostic criteria (13). Note: In our study, a family history of myocardial infarction was considered positive if a first- or second-degree relative had coronary artery disease (myocardial infarction, coronary artery bypass graft surgery, percutaneous coronary intervention) before age 55 in men and 60 in women. A family history of high cholesterol was considered positive if high cholesterol was reported, even without precise values.

| **Category** | **Criteria** |
| --- | --- |
| **Definite FH** | |
| **Laboratory Findings** | - **Adults:** Total cholesterol >290 mg/dL (7.5 mmol/L) or LDL-C >190 mg/dL (4.9 mmol/L) |
|  | - **Children (<16 years):** Total cholesterol >260 mg/dL (6.7 mmol/L) or LDL-C >155 mg/dL (4.0 mmol/L) |
| **Plus at least one of the following:** |  |
| 1. Physical Findings | Tendon xanthomas in the patient or in a first- or second-degree relative |
| 2. Genetic Evidence | DNA-based confirmation of an LDL-receptor mutation, familial defective apo B-100, or a PCSK9 mutation |
| **Possible FH** | |
| **Laboratory Findings** | - **Adults:** Total cholesterol >290 mg/dL (7.5 mmol/L) or LDL-C >190 mg/dL (4.9 mmol/L) |
|  | - **Children (<16 years):** Total cholesterol >260 mg/dL (6.7 mmol/L) or LDL-C >155 mg/dL (4.0 mmol/L) |
| **Plus at least one of the following:** |  |
| 1. Family History of CHD | - Myocardial infarction in a first-degree relative at age ≤60 years |
|  | - Myocardial infarction in a second-degree relative at age ≤50 years |
| 2. Family History of Elevated Cholesterol | - Total cholesterol >290 mg/dL (7.5 mmol/L) in an adult first- or second-degree relative |
|  | - Total cholesterol >260 mg/dL (6.7 mmol/L) in a sibling or child aged <16 years |

Table 2: EAS Consensus Panel Pediatric FH Criteria (14). Note: In our study, positive criteria indicated high probability of FH (Probable FH).

| **LDL-C Threshold** | **Additional Factors** |
| --- | --- |
| ≥5 mmol/L (190 mg/dL) | On two successive occasions after 3 months of diet |
| ≥4 mmol/L (160 mg/dL) | Family history of premature CHD and/or high cholesterol in one parent |
| ≥3.5 mmol/L (130 mg/dL) | Parent with a confirmed genetic diagnosis of FH |

Table 3: Dutch Lipid Clinic Network criteria (15). Note: In our study, a family history of LDL-C ≥95th percentile for age and gender was considered positive if high cholesterol was reported.

| **Criteria** | **Points** |
| --- | --- |
| **Family History** |  |
| - First-degree relative with premature ASCVD (men <55 years, women <60 years)  - First-degree relative with LDL-C ≥95th percentile for age and gender | 1 |
| - Personal or family history of tendon xanthomas and/or arcus cornealis  - Children aged less than 18 years with LDL-C level above the 95th percentile | 2 |
| **Clinical History** |  |
| - Personal history of premature ASCVD (men <55 years, women <60 years) | 2 |
| - Personal history of premature ASCVD in the absence of other risk factors | 1 |
| **Physical Examination** |  |
| - Tendon xanthomas | 6 |
| - Arcus cornealis (age <45 years) | 4 |
| **LDL-Cholesterol Levels** (mmol/L) |  |
| - ≥8.5 | 8 |
| - 6.5–8.4 | 5 |
| - 5.0–6.4 | 3 |
| - 4.0–4.9 | 1 |
| **Molecular Diagnosis** |  |
| - Pathogenic or likely pathogenic variant in *LDLR, APOB*, or *PCSK9* | 8 |
| **Interpretation:** Definite FH >8; Probable FH 6–8; Possible FH 3–5; Unlikely FH <3. | |

Table 4: Simplified Canadian Definition for FH (16). Note: In our study, a family history of atherosclerotic cardiovascular disease (ASCVD) was considered positive if a first-degree relative had ASCVD before age 55 in men and 60 in women. A family history high LDL-C (>95th percentile) was considered positive if high cholesterol was reported.

| **Category** | **Criteria** |
| --- | --- |
| **LDL-C Threshold** | - ≥5.0 mmol/L (≥40 years) |
|  | - ≥4.5 mmol/L (18–39 years) |
|  | - ≥4.0 mmol/L (<18 years) |
| **Major Criteria** | - Tendon xanthomas |
|  | - Pathogenic variant in *LDLR, APOB,* or *PCSK9* |
|  | -LDL-C ≥8.5 mmol/L |
| **Minor Criteria** | - Family history of high LDL-C (>95th percentile) in a first-degree relative |
|  | - History of atherosclerotic cardiovascular disease in the proband or a first-degree relative <55 years (men) or <65 years (women) |
| **FH Classification: Definite FH**: LDL-C threshold + 1 major criterion; **Probable FH**: LDL-C threshold + 1 minor criterion; **Severe Hypercholesterolemia:** LDL-C threshold | |

Table 5: Japanese Atherosclerosis Society FH criteria (under the age of 15) (17). Note: In our study, parental LDL-C ≥ 180 mg/dL was considered positive if high cholesterol was reported. A family history of premature coronary artery disease (CAD) was considered positive if a first- or second-degree relative had CAD before age 55 in men and 60 in women. We differentiated between two groups: probable FH and unlikely FH.

| **Criterion** | **Details** |
| --- | --- |
| 1. Hyper-LDL Cholesterolemia | Untreated LDL-C ≥ 140 mg/dL (confirmed multiple times). |
| 2. Family History of FH | Parent or sibling diagnosed with FH. |
| 3. Parental LDL-C or Family History | Parental LDL-C ≥ 180 mg/dL. |
|  | Family history of premature (men < 55 years of age or women < 65 years of age) coronary artery disease (grandparent or parent). |
| **Diagnosis** |  |
| **FH** | Item 1 + Item 2. |
|  | Item 1 + Item 3, if LDL-C ≥ 180 mg/dL |
|  | Item 1 alone if LDL-C ≥ 250 mg/dL. |
|  | Pathogenic gene mutation for FH. |
| **Probable FH** | Item 1 + Item 3. |
|  | Item 1 alone if LDL-C ≥ 180 mg/dL |
| Diagnosis requires ruling out secondary dyslipidemia. LDL-C ≥ 250 mg/dL or presence of tendon xanthomas suggests Homozygous FH. | |

Table 6: Japanese Atherosclerosis Society FH criteria (15 years of age or older) (18). Note: In our study, a family history of premature coronary artery disease (CAD) was considered positive if a first- or second-degree relative had CAD before age 55 in men and 60 in women.

| **Criterion** | **Details** |
| --- | --- |
| 1. Hyper-LDL Cholesterolemia | Untreated LDL-C ≥ 180 mg/dL (confirmed multiple times). |
| 2. Tendon Xanthomas or Xanthoma Tuberosum | Tendon thickening (hands, elbows, knees) or Achilles tendon hypertrophy (≥9 mm on X-ray). |
|  | Presence of subcutaneous xanthomas (excluding xanthelasma). |
| 3. Family History of FH or CAD | Family history of FH or premature (men < 55 years of age or women < 65 years of age) coronary artery disease (within the patient's second-degree relatives). |
| **Diagnosis** |  |
| **FH** | Two or more criteria from items 1, 2, 3. |
|  | LDL-C ≥ 250 mg/dL alone strongly suggests FH. |
|  | Pathogenic mutation for confirms diagnosis. |
| Diagnosis requires ruling out secondary dyslipidemia. | |

**Development of the New Clinical Score (FH-PeDS)**

We developed the FH Pediatric Score **(FH-PeDS)** in R using a multi-step approach to convert numerical variables (LDL-C, HDL-C, TAG, BMI Z-score, Lp(a), etc.) into clinically relevant categories with distinct cutoffs. The SI cohort was used for the development of the score, while the PO cohort served for additional external validation. Initially, LOESS plots generated with ggplot2 visualized the relationship between each continuous predictor and the probability of FH, guiding the selection of clinically meaningful cutoffs (19). ROC analyses iteratively explored thresholds (0–100% in 5% increments) to determine sensitivity and specificity, alongside k-means clustering and quintile-based methods to identify natural groupings. Each numerical parameter was categorized into clinically pertinent groups (Low, Moderate, High, Very High, Severe), with category counts adjusted based on clinical relevance.

Family history categories were constructed considering the structure and criteria used in the Dutch Lipid Clinic Network (DLCN), given that family history data in clinical practice is frequently collected according to DLCN requirements (15). We aligned these categories closely with DLCN principles to ensure practicality and ease of clinical adoption.

Logistic regression models evaluated the predictive power of these categories, alongside other categorical data, selecting significant predictors from a broader set, including age, sex, family history of high cholesterol, premature cardiovascular disease, tendinous xanthoma or arcus cornealis, HDL-C, LDL-C, TAG, and BMI Z-score. Stratified variables underwent logistic regression to extract coefficients, rounded to the nearest 0.5 and multiplied by 2 to yield whole-number weights. Some weights were minimally adjusted to simplify calculations while maintaining predictive accuracy and clinical relevance. Although certain categories could yield negative points, the total score was bounded at a minimum of 0, ensuring no negative final scores. Model effectiveness was assessed through calculation and visualization of the area under the ROC curve (AUC). The score was developed using the SI cohort and externally validated in the PO cohort, demonstrating robustness and generalizability as a diagnostic tool for FH.

For cascade screening, we preliminary explored incorporating additional points for genetically confirmed first-degree relatives into the family history component to enhance the **FH-PeDS**' performance, as detailed in subsequent sections.

# Established FH Scores Overlap Analysis

**Figures 5-7** illustrates the variability in overlap among individuals diagnosed with FH based on different diagnostic criteria and genetic confirmation. The largest overlap includes 235 individuals identified as FH-positive across all criteria (47.4% of all genetically confirmed cases). However, 54 genetically confirmed FH-positive individuals (10.9% of all such cases) were missed by all clinical diagnostic criteria, underscoring potential gaps in sensitivity. While the overall overlap among criteria is substantial, it varies notably. Concordance is influenced by the number of individuals diagnosed by each criterion (set size), with EAS, which has the largest set size, showing the highest overlap with other criteria (100% with CAN and 98% with DLCN). In contrast, DLCN, having the smallest set size, demonstrates smaller overlaps with other criteria (48.0% to 51.6%) but the best alignment with genetically confirmed FH cases (54.8%). Similarly, genetically confirmed FH-positive cases exhibit the highest overlap with DLCN (78.4%), while overlap with other criteria is more limited, ranging from 59.6% to 60.8%.

# Preliminary Validation of Additional Points in FH-PeDS for a Genetically Positive First-Degree Relative

As part of the Slovenian universal FH screening program, we perform genetic testing on children identified through initial screening and subsequently offer cascade testing to their first-degree relatives. In this third step of the program, 104 children (<18 years) from the Slovenian FH registry were referred for genetic analysis. For validation of additional points allocated for a genetically positive first-degree relative in **FH-PeDS**, we excluded children who were on lipid-lowering therapy at their first examination or who lacked any required measurements for calculating the **FH-PeDS** score, leaving 67 children (41 genetically positive for FH, 26 genetically negative) for this analysis.

We applied the proposed FH-PeDS scoring system to 67 cascade-screened children, systematically varying the “family history” component from 0 to +7 points. These cases were then merged with our original universal screening cohort to assess the impact of additional family history points on score performance. In the cascade-only cohort (n=67), the FH-PeDS score without additional points for genetically confirmed first-degree relatives achieved an AUC of 0.897. The original universal screening cohort (excluding cascade cases) yielded an AUC of 0.906. Upon merging these cohorts, the baseline score (0 additional points) produced an AUC of 0.905. Incremental assignment of +1 to +7 points resulted in the following AUC values:

- +1 point: 0.905
- +2 points: 0.906
- +3 points: 0.909
- +4 points: 0.910
- +5 points: 0.910
- +6 points: 0.910
- +7 points: 0.909

Analysis of AUC trends indicates peak performance within the range of +4 to +6 additional points. Therefore, we selected the minimal effective increment (+4 points) to optimize FH-PeDS performance for cascade screening. We also evaluated sensitivity and specificity at different score thresholds. At our proposed threshold of 6 (possible FH) with the addition of +4 points, sensitivity was 91.9%, and specificity was 71.3%. At a higher threshold of 10, sensitivity decreased to 62.5% while specificity increased to 93.8%.

These preliminary findings suggest awarding +4 points for a genetically confirmed first-degree relative may optimize the FH-PeDS performance. However, given our relatively small cascade cohort and primary focus on universal or opportunistic screening, validation in larger cascade cohorts is essential. Nevertheless, this analysis provides an important foundation for refining the FH-PeDS scoring system.

# Table 7: Sensitivity, Specificity, Positive Predictive Value (PPV) and Negative Predictive Value (NPV) for different cut-offs of the New Clinical FH Score (FH-PeDS).

| **Threshold** | **Combined Cohort** | | | | **Slovenian Cohort** | | | | **Portuguese Cohort** | | | |
| --- | --- | --- | --- | --- | --- | --- | --- | --- | --- | --- | --- | --- |
|  | **Sensitivity** | **Specificity** | **PPV** | **NPV** | **Sensitivity** | **Specificity** | **PPV** | **NPV** | **Sensitivity** | **Specificity** | **PPV** | **NPV** |
| 0 | 1.0000 | 0.0000 | 0.2884 | NA | 1.0000 | 0.0000 | 0.2385 | NA | 1.0000 | 0.0000 | 0.4984 | NA |
| 1 | 0.9937 | 0.2365 | 0.3453 | 0.9892 | 0.9905 | 0.2725 | 0.2989 | 0.9892 | 1.0000 | 0.0000 | 0.4984 | NA |
| 2 | 0.9915 | 0.2751 | 0.3567 | 0.9877 | 0.9873 | 0.3171 | 0.3117 | 0.9877 | 1.0000 | 0.0063 | 0.5000 | 1.0000 |
| 3 | 0.9831 | 0.3890 | 0.3947 | 0.9827 | 0.9747 | 0.4450 | 0.3548 | 0.9825 | 1.0000 | 0.0063 | 0.5000 | 1.0000 |
| 4 | 0.9789 | 0.4602 | 0.4236 | 0.9817 | 0.9684 | 0.5273 | 0.3908 | 0.9815 | 1.0000 | 0.0316 | 0.5065 | 1.0000 |
| 5 | 0.9450 | 0.6161 | 0.4994 | 0.9651 | 0.9209 | 0.6908 | 0.4826 | 0.9654 | 0.9936 | 0.1392 | 0.5342 | 0.9565 |
| 6 | 0.9387 | 0.6427 | 0.5157 | 0.9628 | 0.9114 | 0.7195 | 0.5044 | 0.9629 | 0.9936 | 0.1519 | 0.5379 | 0.9600 |
| 7 | 0.8901 | 0.7429 | 0.5839 | 0.9434 | 0.8481 | 0.8167 | 0.5916 | 0.9450 | 0.9745 | 0.2722 | 0.5709 | 0.9149 |
| 8 | 0.8668 | 0.7678 | 0.6021 | 0.9343 | 0.8165 | 0.8414 | 0.6172 | 0.9361 | 0.9682 | 0.2975 | 0.5779 | 0.9038 |
| 9 | 0.7378 | 0.8698 | 0.6966 | 0.8911 | 0.6456 | 0.9286 | 0.7391 | 0.8932 | 0.9236 | 0.4937 | 0.6444 | 0.8667 |
| 10 | 0.7104 | 0.8826 | 0.7104 | 0.8826 | 0.6076 | 0.9415 | 0.7649 | 0.8845 | 0.9172 | 0.5063 | 0.6486 | 0.8602 |
| 11 | 0.5624 | 0.9494 | 0.8185 | 0.8426 | 0.4525 | 0.9812 | 0.8827 | 0.8512 | 0.7834 | 0.7468 | 0.7546 | 0.7763 |
| 12 | 0.5455 | 0.9529 | 0.8243 | 0.8380 | 0.4272 | 0.9822 | 0.8824 | 0.8456 | 0.7834 | 0.7658 | 0.7688 | 0.7806 |
| 13 | 0.3298 | 0.9837 | 0.8914 | 0.7836 | 0.2627 | 0.9950 | 0.9432 | 0.8116 | 0.4650 | 0.9114 | 0.8391 | 0.6316 |
| 14 | 0.3150 | 0.9854 | 0.8976 | 0.7802 | 0.2437 | 0.9960 | 0.9506 | 0.8079 | 0.4586 | 0.9177 | 0.8471 | 0.6304 |
| 15 | 0.0951 | 1.0000 | 1.0000 | 0.7317 | 0.0696 | 1.0000 | 1.0000 | 0.7744 | 0.1465 | 1.0000 | 1.0000 | 0.5411 |
| 16 | 0.0846 | 1.0000 | 1.0000 | 0.7294 | 0.0601 | 1.0000 | 1.0000 | 0.7726 | 0.1338 | 1.0000 | 1.0000 | 0.5374 |

# Table 8: Training cohort and testing cohort (ML-FH-PeDS) overview of characteristics.

| **Parameter** | **Category** | **Training cohort** | | | | **Testing cohort** | | | | **p-value§** |
| --- | --- | --- | --- | --- | --- | --- | --- | --- | --- | --- |
|  |  | **Overall, N = 795 (60%)** | **FH positive, N = 190 (23.9%)** | **FH negative, N = 605 (76.1%)** | **p-value†** | **Overall, N = 530 (40%)** | **FH positive, N = 126 (23.8%)** | **FH negative, N = 404 (76.2%)** | **p-value‡** |  |
| Sex | Female | 449 (56.5%) | 90 (47.4%) | 359 (59.3%) | 0.01 | 318 (60%) | 75 (59.5%) | 243 (60.1%) | 0.98 | 0.60 |
|  | Male | 346 (43.5%) | 100 (52.6%) | 246 (40.7%) |  | 212 (40%) | 51 (40.5%) | 161 (39.9%) |  |  |
| Age | | 6.3 (5.7–7.5) | 6.1 (5.6–7.2) | 6.4 (5.8–7.7) | <0.01 | 6.4 (5.8–7.8) | 6.2 (5.6–7.4) | 6.5 (5.8–7.9) | 0.14 | 0.68 |
| High cholesterol in family | No | 139 (17.5%) | 17 (8.9%) | 122 (20.2%) | <0.01 | 89 (16.8%) | 11 (8.7%) | 78 (19.3%) | <0.01 | 1.00 |
|  | Only first degree relative | 156 (19.6%) | 43 (22.6%) | 113 (18.7%) |  | 106 (20.0%) | 22 (17.5%) | 84 (20.8%) |  |  |
|  | Only second degree relative | 178 (22.4%) | 22 (11.6%) | 156 (25.8%) |  | 124 (23.4%) | 25 (19.8%) | 99 (24.5%) |  |  |
|  | First and second degree relative | 322 (40.5%) | 108 (56.8%) | 214 (35.4%) |  | 211 (39.8%) | 68 (54.0%) | 143 (35.4%) |  |  |
| Family history of premature coronary artery disease | No | 653 (82.1%) | 141 (74.2%) | 512 (84.6%) | 0.02 | 448 (84.5%) | 98 (77.8%) | 350 (86.6%) | 0.07 | 0.94 |
|  | Only first degree relative | 21 (2.6%) | 6 (3.2%) | 15 (2.5%) |  | 14 (2.6%) | 3 (2.4%) | 11 (2.7%) |  |  |
|  | Only second degree relative | 119 (15.0%) | 42 (22.1%) | 77 (12.7%) |  | 67 (12.6%) | 25 (19.8%) | 42 (10.4%) |  |  |
|  | First and second degree relative | 2 (0.3%) | 1 (0.5%) | 1 (0.2%) |  | 1 (0.2 %) | 0 (0%) | 1 (0.2%) |  |  |
| Family history of premature peripheral artery disease | No | 742 (93.3%) | 177 (93.2%) | 565 (93.4%) | 1.00 | 497 (93.8%) | 114 (90.5%) | 383 (94.8%) | NA | 0.94 |
|  | Only first degree relative | 4 (0.5%) | 1 (0.5%) | 3 (0.5%) |  | 3 (0.6%) | 2 (1.6%) | 1 (0.2%) |  |  |
|  | Only second degree relative | 47 (5.9%) | 11 (5.8%) | 36 (6.0%) |  | 30 (5.7%) | 10 (7.9%) | 20 (5.0%) |  |  |
|  | First and second degree relative | 2 (0.3%) | 1 (0.5%) | 1 (0.2%) |  | 0 (0%) | 0 (0%) | 0 (0%) |  |  |
| Family history of tendinous xanthoma or xanthelasma | No | 793 (99.7%) | 190 (100%) | 603 (99.7%) | 1.00 | 525 (99.1%) | 122 (96.8%) | 403 (99.8%) | 0.029 | 0.60 |
|  | Yes | 2 (0.3%) | 0 (0%) | 2 (0.3%) |  | 5 (0.9%) | 4 (3.2%) | 1 (0.2%) |  |  |
| Family history of arcus cornealis | No | 792 (99.6%) | 187 (98.4%) | 605 (100%) | 0.02 | 526 (99.2%) | 124 (98.4%) | 402 (99.5%) | 0.621 | 0.94 |
|  | Yes | 3 (0.4%) | 3 (1.6%) | 0 (0%) |  | 4 (0.8%) | 2 (1.6%) | 2 (0.5%) |  |  |
| TC (mmol/L) | | 5.6 (5.1–6.3) | 6.6 (6.1–7.3) | 5.4 (5.0–5.9) | <0.01 | 5.6 (5.1–6.3) | 6.6 (6.0–7.4) | 5.4 (4.9–5.9) | <0.01 | 0.88 |
| TC (mg/dL) | | 217 (197–244) | 255 (236–282) | 209 (193–228) | <0.01 | 217 (197–244) | 255 (232–286) | 209 (190–228) | <0.01 | 0.88 |
| HDL-C (mmol/L) | | 1.5 (1.3–1.8) | 1.4 (1.2–1.6) | 1.6 (1.3–1.8) | <0.01 | 1.5 (1.3–1.7) | 1.4 (1.2–1.5) | 1.5 (1.3–1.8) | <0.01 | 0.88 |
| HDL-C (mg/dL) | | 58 (50–70) | 54 (46–62) | 62 (50–70) | <0.01 | 58 (50–66) | 54 (46–58) | 58 (50–70) | <0.01 | 0.88 |
| LDL-C (mmol/L) | | 3.6 (3.1–4.3) | 4.9 (4.3–5.5) | 3.4 (2.9–3.8) | <0.01 | 3.6 (3.1–4.3) | 4.8 (4.1–5.6) | 3.4 (2.9–3.8) | <0.01 | 0.88 |
| LDL-C (mg/dL) | | 139 (120–166) | 190 (166–213) | 131 (112–147) | <0.01 | 139 (120–166) | 186 (159–217) | 131 (112–147) | <0.01 | 0.88 |
| TAG (mmol/L) | | 0.9 (0.7–1.3) | 0.9 (0.7–1.3) | 0.9 (0.7–1.3) | 0.33 | 0.8 (0.6–1.3) | 0.8 (0.6–1.1) | 0.9 (0.6–1.3) | <0.01 | 0.68 |
| TAG (mg/dL) | | 80 (62–115) | 80 (62–115) | 80 (62–115) | 0.33 | 71 (53–115) | 71 (53–97) | 80 (53–115) | <0.01 | 0.68 |
| Lp(a) (mg/L) | | 124 (<99.4–402.5)* | 109 (<99.4–309) | 136 (<99.4–438) | 0.04 | 135.5 (<99.4–441.5) | 104 (<99.4–359.5)** | 143 (<99.4–452.5) | 0.43 | 0.88 |
| BMI Z-Score | | 0.15 (-0.57–1.05) | 0.09 (-0.48–1.03) | 0.16 (-0.65–1.08) | 0.86 | 0.13 (-0.56–1.17) | 0.04 (-0.71–0.98) | 0.16 (-0.50–1.20) | 0.10 | 0.88 |
| Data are absolute frequencies (proportions in %) and median (first quartile–third quartile). Chi-Squared Test was used for comparison of categorical variables; Mann-Whitney Test was used for comparison of numerical variables due to non-normal distribution. To control the false discovery rate, p-values were adjusted using the Benjamini-Hochberg method, with significance set at an adjusted p < 0.05.  **Legend:** FH = familial hypercholesterolaemia; TC = Total cholesterol; HDL-C = High-density lipoprotein cholesterol; LDL-C = Low-density lipoprotein cholesterol; TAG = Triglycerides; Lp(a) = Lipoprotein(a); BMI = Body mass index.  **Footnotes:** † p-value for comparisons between FH positive vs FH negative within the training cohort; ‡ p-value for comparisons between FH positive vs FH negative within the testing cohort; § p-value for comparisons between training and testing cohort; * Missing 69 values; ** Missing 60 values. | | | | | | | | | | |

# Table 9: Performance metrics of the machine learning model (ML-FH-PeDS) at different threshold levels.

| **Model threshold** | **Testing cohort** | | | **External validation cohort** | | |
| --- | --- | --- | --- | --- | --- | --- |
|  | **Specificity** | **Sensitivity/Recall** | **Precision** | **Specificity** | **Sensitivity/Recall** | **Precision** |
| 0.01 | 0.0965 | 0.9921 | 0.2551 | 0.0058 | 1.0000 | 0.4956 |
| 0.02 | 0.1931 | 0.9921 | 0.2772 | 0.0058 | 1.0000 | 0.4956 |
| 0.03 | 0.3020 | 0.9841 | 0.3054 | 0.0174 | 1.0000 | 0.4985 |
| 0.04 | 0.3837 | 0.9683 | 0.3288 | 0.0174 | 1.0000 | 0.4985 |
| 0.05 | 0.4381 | 0.9603 | 0.3477 | 0.0291 | 1.0000 | 0.5015 |
| 0.06 | 0.5025 | 0.9603 | 0.3758 | 0.0523 | 1.0000 | 0.5076 |
| 0.07 | 0.5569 | 0.9444 | 0.3993 | 0.0640 | 1.0000 | 0.5106 |
| 0.08 | 0.5965 | 0.9365 | 0.4199 | 0.0756 | 1.0000 | 0.5138 |
| 0.09 | 0.6337 | 0.9286 | 0.4415 | 0.0872 | 0.9940 | 0.5154 |
| 0.1 | 0.6683 | 0.9286 | 0.4661 | 0.0930 | 0.9940 | 0.5170 |
| 0.11 | 0.6881 | 0.9286 | 0.4815 | 0.1105 | 0.9940 | 0.5219 |
| 0.12 | 0.7030 | 0.9206 | 0.4915 | 0.1163 | 0.9881 | 0.5220 |
| 0.13 | 0.7228 | 0.9127 | 0.5066 | 0.1221 | 0.9881 | 0.5237 |
| 0.14 | 0.7426 | 0.8968 | 0.5207 | 0.1395 | 0.9881 | 0.5287 |
| 0.15 | 0.7500 | 0.8968 | 0.5280 | 0.1512 | 0.9881 | 0.5321 |
| 0.16 | 0.7649 | 0.8889 | 0.5411 | 0.1628 | 0.9881 | 0.5355 |
| 0.17 | 0.7673 | 0.8889 | 0.5437 | 0.1919 | 0.9881 | 0.5443 |
| 0.18 | 0.7772 | 0.8889 | 0.5545 | 0.2093 | 0.9881 | 0.5497 |
| 0.19 | 0.7921 | 0.8889 | 0.5714 | 0.2151 | 0.9821 | 0.5500 |
| 0.2 | 0.8020 | 0.8730 | 0.5789 | 0.2209 | 0.9762 | 0.5503 |
| 0.21 | 0.8144 | 0.8651 | 0.5924 | 0.2326 | 0.9762 | 0.5541 |
| 0.22 | 0.8193 | 0.8571 | 0.5967 | 0.2384 | 0.9762 | 0.5559 |
| 0.23 | 0.8267 | 0.8413 | 0.6023 | 0.2500 | 0.9762 | 0.5597 |
| 0.24 | 0.8292 | 0.8413 | 0.6057 | 0.2558 | 0.9702 | 0.5601 |
| 0.25 | 0.8342 | 0.8254 | 0.6082 | 0.2616 | 0.9702 | 0.5621 |
| 0.26 | 0.8416 | 0.8016 | 0.6121 | 0.2674 | 0.9643 | 0.5625 |
| 0.27 | 0.8490 | 0.7937 | 0.6211 | 0.2674 | 0.9643 | 0.5625 |
| 0.28 | 0.8564 | 0.7857 | 0.6306 | 0.2791 | 0.9643 | 0.5664 |
| 0.29 | 0.8589 | 0.7619 | 0.6275 | 0.2965 | 0.9643 | 0.5724 |
| 0.3 | 0.8688 | 0.7460 | 0.6395 | 0.3023 | 0.9583 | 0.5730 |
| 0.31 | 0.8738 | 0.7381 | 0.6458 | 0.3198 | 0.9583 | 0.5791 |
| 0.32 | 0.8837 | 0.7302 | 0.6619 | 0.3314 | 0.9583 | 0.5833 |
| 0.33 | 0.8911 | 0.7222 | 0.6741 | 0.3547 | 0.9583 | 0.5919 |
| 0.34 | 0.9010 | 0.7143 | 0.6923 | 0.3779 | 0.9583 | 0.6007 |
| 0.35 | 0.9059 | 0.7063 | 0.7008 | 0.3837 | 0.9583 | 0.6030 |
| 0.36 | 0.9109 | 0.7063 | 0.7120 | 0.4012 | 0.9524 | 0.6084 |
| 0.37 | 0.9134 | 0.6905 | 0.7131 | 0.4070 | 0.9524 | 0.6107 |
| 0.38 | 0.9134 | 0.6746 | 0.7083 | 0.4070 | 0.9524 | 0.6107 |
| 0.39 | 0.9208 | 0.6667 | 0.7241 | 0.4186 | 0.9524 | 0.6154 |
| 0.4 | 0.9208 | 0.6667 | 0.7241 | 0.4360 | 0.9464 | 0.6211 |
| 0.41 | 0.9282 | 0.6667 | 0.7434 | 0.4477 | 0.9405 | 0.6245 |
| 0.42 | 0.9332 | 0.6429 | 0.7500 | 0.4535 | 0.9345 | 0.6255 |
| 0.43 | 0.9332 | 0.6429 | 0.7500 | 0.4826 | 0.9286 | 0.6367 |
| 0.44 | 0.9356 | 0.6349 | 0.7547 | 0.4942 | 0.9286 | 0.6420 |
| 0.45 | 0.9356 | 0.6270 | 0.7524 | 0.5058 | 0.9286 | 0.6473 |
| 0.46 | 0.9381 | 0.6190 | 0.7573 | 0.5116 | 0.9286 | 0.6500 |
| 0.47 | 0.9381 | 0.6190 | 0.7573 | 0.5174 | 0.9286 | 0.6527 |
| 0.48 | 0.9406 | 0.6190 | 0.7647 | 0.5233 | 0.9226 | 0.6540 |
| 0.49 | 0.9455 | 0.6111 | 0.7778 | 0.5349 | 0.9226 | 0.6596 |
| 0.5 | 0.9480 | 0.6111 | 0.7857 | 0.5523 | 0.9226 | 0.6681 |
| 0.51 | 0.9530 | 0.5952 | 0.7979 | 0.5640 | 0.9226 | 0.6739 |
| 0.52 | 0.9554 | 0.5873 | 0.8043 | 0.5756 | 0.9226 | 0.6798 |
| 0.53 | 0.9579 | 0.5794 | 0.8111 | 0.5814 | 0.9107 | 0.6800 |
| 0.54 | 0.9604 | 0.5714 | 0.8182 | 0.5872 | 0.9048 | 0.6816 |
| 0.55 | 0.9604 | 0.5714 | 0.8182 | 0.6105 | 0.8988 | 0.6927 |
| 0.56 | 0.9604 | 0.5556 | 0.8140 | 0.6105 | 0.8869 | 0.6898 |
| 0.57 | 0.9653 | 0.5476 | 0.8313 | 0.6221 | 0.8869 | 0.6963 |
| 0.58 | 0.9653 | 0.5397 | 0.8293 | 0.6512 | 0.8810 | 0.7115 |
| 0.59 | 0.9653 | 0.5317 | 0.8272 | 0.6628 | 0.8750 | 0.7171 |
| 0.6 | 0.9653 | 0.5317 | 0.8272 | 0.6744 | 0.8690 | 0.7228 |
| 0.61 | 0.9678 | 0.5159 | 0.8333 | 0.6802 | 0.8690 | 0.7264 |
| 0.62 | 0.9703 | 0.5159 | 0.8442 | 0.6860 | 0.8690 | 0.7300 |
| 0.63 | 0.9703 | 0.5159 | 0.8442 | 0.6860 | 0.8690 | 0.7300 |
| 0.64 | 0.9703 | 0.5079 | 0.8421 | 0.7035 | 0.8631 | 0.7398 |
| 0.65 | 0.9703 | 0.4841 | 0.8356 | 0.7093 | 0.8571 | 0.7423 |
| 0.66 | 0.9703 | 0.4683 | 0.8310 | 0.7209 | 0.8512 | 0.7487 |
| 0.67 | 0.9703 | 0.4603 | 0.8406 | 0.7326 | 0.8512 | 0.7566 |
| 0.68 | 0.9752 | 0.4444 | 0.8485 | 0.7384 | 0.8393 | 0.7581 |
| 0.69 | 0.9752 | 0.4444 | 0.8485 | 0.7558 | 0.8214 | 0.7667 |
| 0.7 | 0.9777 | 0.4365 | 0.8594 | 0.7616 | 0.8036 | 0.7670 |
| 0.71 | 0.9777 | 0.4286 | 0.8571 | 0.7616 | 0.7917 | 0.7644 |
| 0.72 | 0.9777 | 0.4127 | 0.8525 | 0.7849 | 0.7917 | 0.7824 |
| 0.73 | 0.9777 | 0.4127 | 0.8525 | 0.8023 | 0.7917 | 0.7964 |
| 0.74 | 0.9827 | 0.3968 | 0.8772 | 0.8081 | 0.7619 | 0.7950 |
| 0.75 | 0.9827 | 0.3889 | 0.8750 | 0.8140 | 0.7500 | 0.7975 |
| 0.76 | 0.9827 | 0.3889 | 0.8750 | 0.8198 | 0.7321 | 0.7987 |
| 0.77 | 0.9827 | 0.3889 | 0.8750 | 0.8314 | 0.7143 | 0.8054 |
| 0.78 | 0.9827 | 0.3889 | 0.8750 | 0.8547 | 0.7143 | 0.8276 |
| 0.79 | 0.9827 | 0.3651 | 0.8679 | 0.8663 | 0.7083 | 0.8380 |
| 0.8 | 0.9851 | 0.3571 | 0.8824 | 0.8663 | 0.7024 | 0.8369 |
| 0.81 | 0.9851 | 0.3571 | 0.8824 | 0.8837 | 0.6905 | 0.8529 |
| 0.82 | 0.9851 | 0.3492 | 0.8800 | 0.8895 | 0.6845 | 0.8582 |
| 0.83 | 0.9901 | 0.3413 | 0.9149 | 0.8895 | 0.6845 | 0.8582 |
| 0.84 | 0.9950 | 0.3333 | 0.9545 | 0.9012 | 0.6786 | 0.8702 |
| 0.85 | 0.9950 | 0.3254 | 0.9535 | 0.9012 | 0.6786 | 0.8702 |
| 0.86 | 0.9950 | 0.3095 | 0.9512 | 0.9128 | 0.6667 | 0.8819 |
| 0.87 | 0.9950 | 0.3016 | 0.9500 | 0.9128 | 0.6250 | 0.8750 |
| 0.88 | 0.9950 | 0.2778 | 0.9459 | 0.9186 | 0.6071 | 0.8793 |
| 0.89 | 0.9950 | 0.2540 | 0.9412 | 0.9244 | 0.5833 | 0.8829 |
| 0.9 | 0.9950 | 0.2222 | 0.9333 | 0.9302 | 0.5714 | 0.8889 |
| 0.91 | 0.9975 | 0.2143 | 0.9643 | 0.9419 | 0.5417 | 0.9010 |
| 0.92 | 0.9975 | 0.2063 | 0.9630 | 0.9419 | 0.5417 | 0.9010 |
| 0.93 | 0.9975 | 0.1905 | 0.9600 | 0.9477 | 0.5119 | 0.9053 |
| 0.94 | 0.9975 | 0.1825 | 0.9583 | 0.9651 | 0.4821 | 0.9310 |
| 0.95 | 0.9975 | 0.1825 | 0.9583 | 0.9651 | 0.4583 | 0.9277 |
| 0.96 | 0.9975 | 0.1508 | 0.9500 | 0.9709 | 0.4226 | 0.9342 |
| 0.97 | 0.9975 | 0.1349 | 0.9444 | 0.9709 | 0.3690 | 0.9254 |
| 0.98 | 0.9975 | 0.1270 | 0.9412 | 0.9884 | 0.3095 | 0.9630 |
| 0.99 | 0.9975 | 0.0794 | 0.9091 | 0.9942 | 0.2500 | 0.9767 |
| 1 | 1.0000 | 0.0000 | – | 1.0000 | 0.0000 | – |

# Table 10: Parameter weights in machine learning model (ML-FH-PeDS) for predicting familial hypercholesterolemia (FH).

| **Parameter** | **Weight** | **OR** | **Parameter** | **Weight** | **OR** |
| --- | --- | --- | --- | --- | --- |
| LDL cholesterol [mmol/L] | 1.280 | 3.60 | Premature vascular disease in first and second degree relative | 0.008 | 1.01 |
| Total cholesterol [mmol/L] | 1.030 | 2.80 | Age [years] | -0.003 | 1.00 |
| High cholesterol in first and second degree relative | 0.372 | 1.45 | BMI Z-score | -0.047 | 0.95 |
| Sex* | 0.277 | 1.32 | Negative family history of premature CAD | -0.090 | 0.91 |
| Negative family history of premature PAD | 0.150 | 1.16 | Positive family history of tendinous xanthoma/ xanthelasma | -0.120 | 0.89 |
| Positive family history of arcus cornealis | 0.137 | 1.15 | High cholesterol in second degree relative | -0.153 | 0.86 |
| High cholesterol in first degree relative | 0.136 | 1.15 | Premature vascular disease in second degree relative | -0.185 | 0.83 |
| Premature CAD in second degree relative | 0.053 | 1.05 | Negative family history for high cholesterol | -0.356 | 0.70 |
| Premature vascular disease in first degree relative | 0.026 | 1.03 | Lipoprotein(a) [mg/L] | -0.361 | 0.70 |
| Premature CAD in first and second degree relative | 0.019 | 1.02 | Triglycerides [mmol/L] | -0.466 | 0.63 |
| Premature CAD in first degree relative | 0.016 | 1.02 | HDL cholesterol [mmol/L] | -0.782 | 0.46 |
| **A change in a feature by 1 unit (1 standard deviation for a continuous or from 0 to 1 in a one-hot encoded binary feature) changes the odds ratio by a factor of exp(weight). Note that logistic regression weights are given with respect to the normalized values for continuous features and one-hot encodings for categorical features.**  **Legend:** OR = Odds ratio; LDL-C = Low-density lipoprotein cholesterol; CAD = coronary artery disease; BMI = Body mass index; HDL-C = High-density lipoprotein cholesterol. **Footnotes:** *Higher likelihood in males compared to females. | | | | | |

# Figure 1: Bar Plots of Diagnostic Criteria for Familial Hypercholesterolemia (FH) in Slovenian Registry.


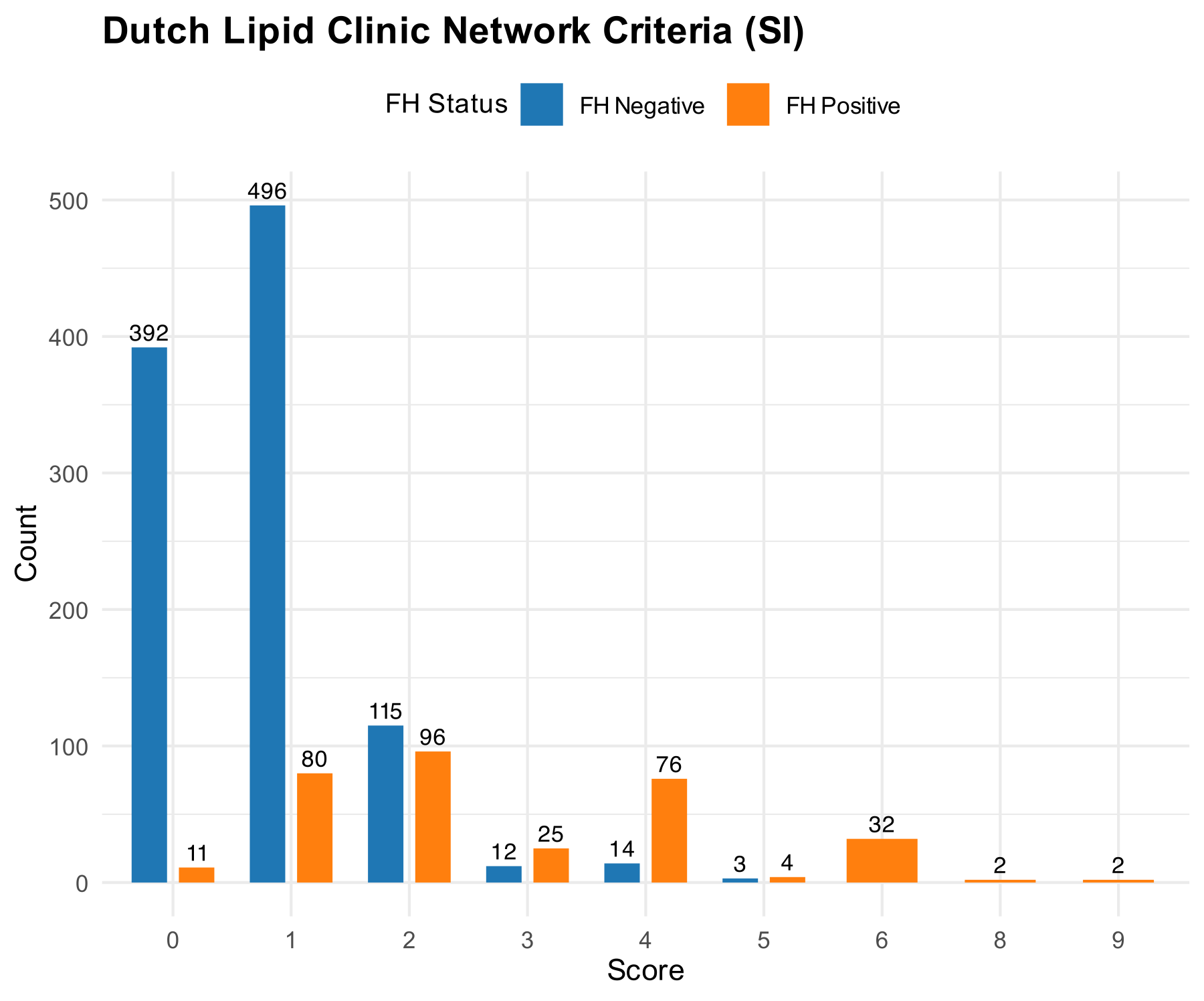

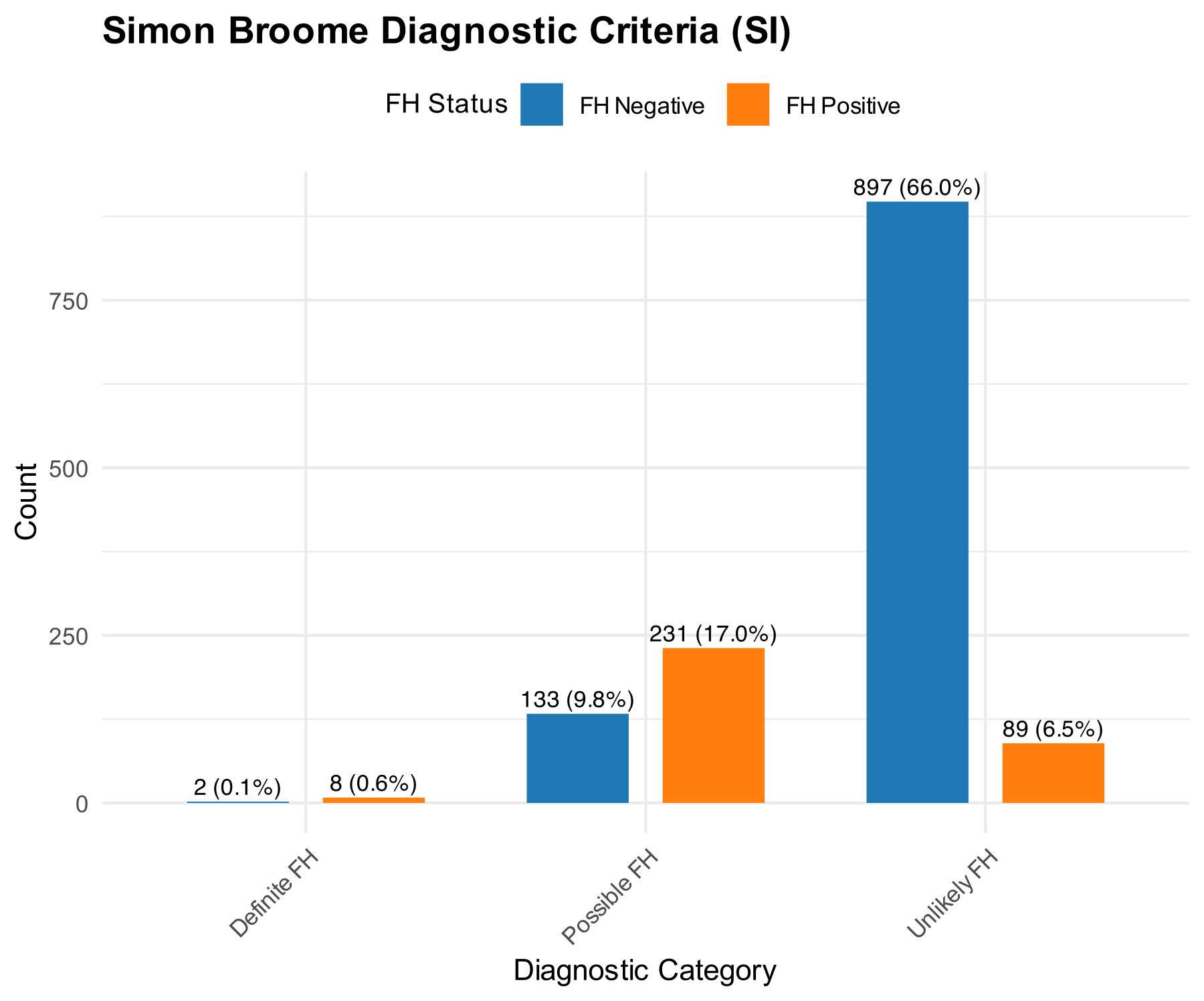

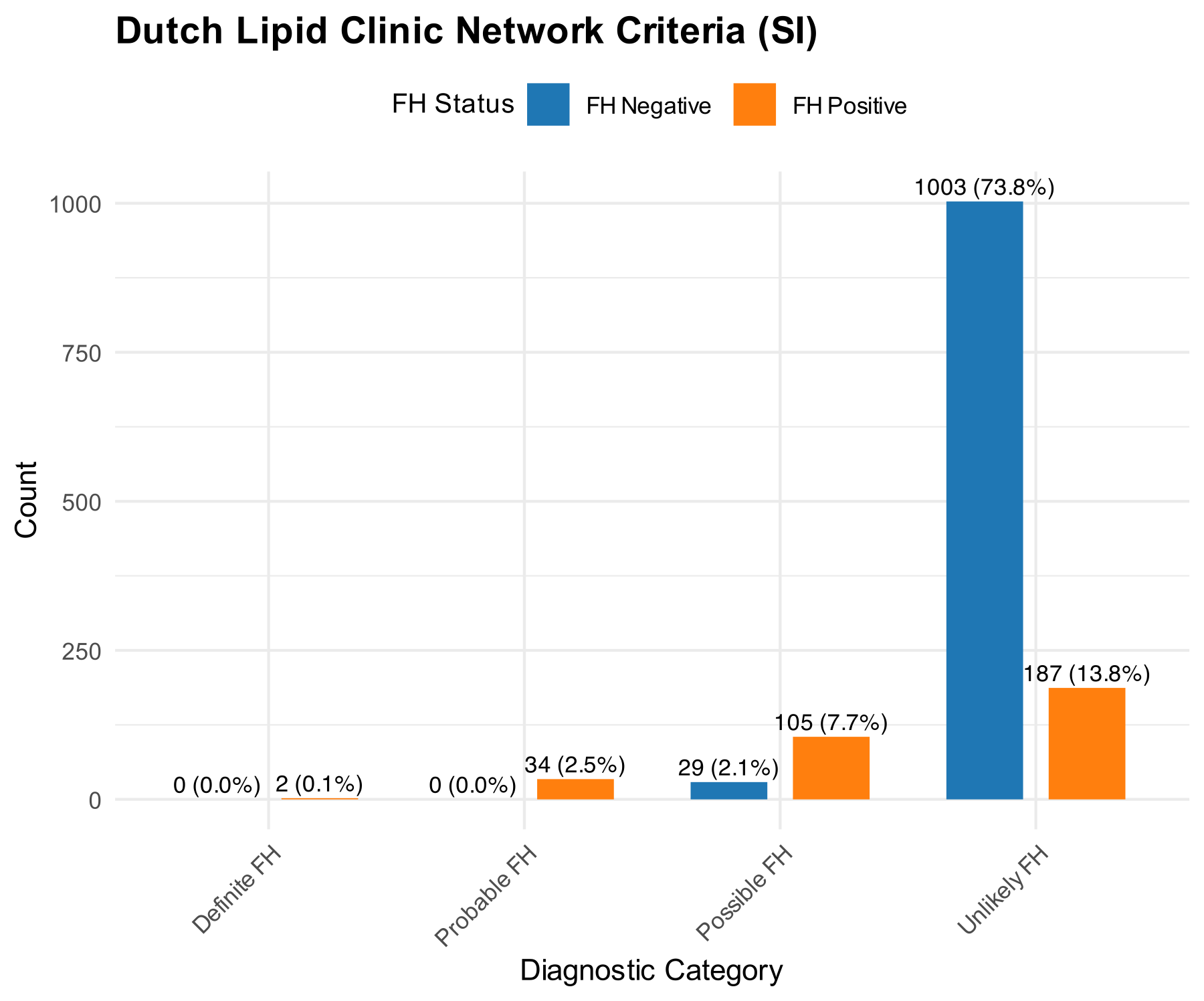

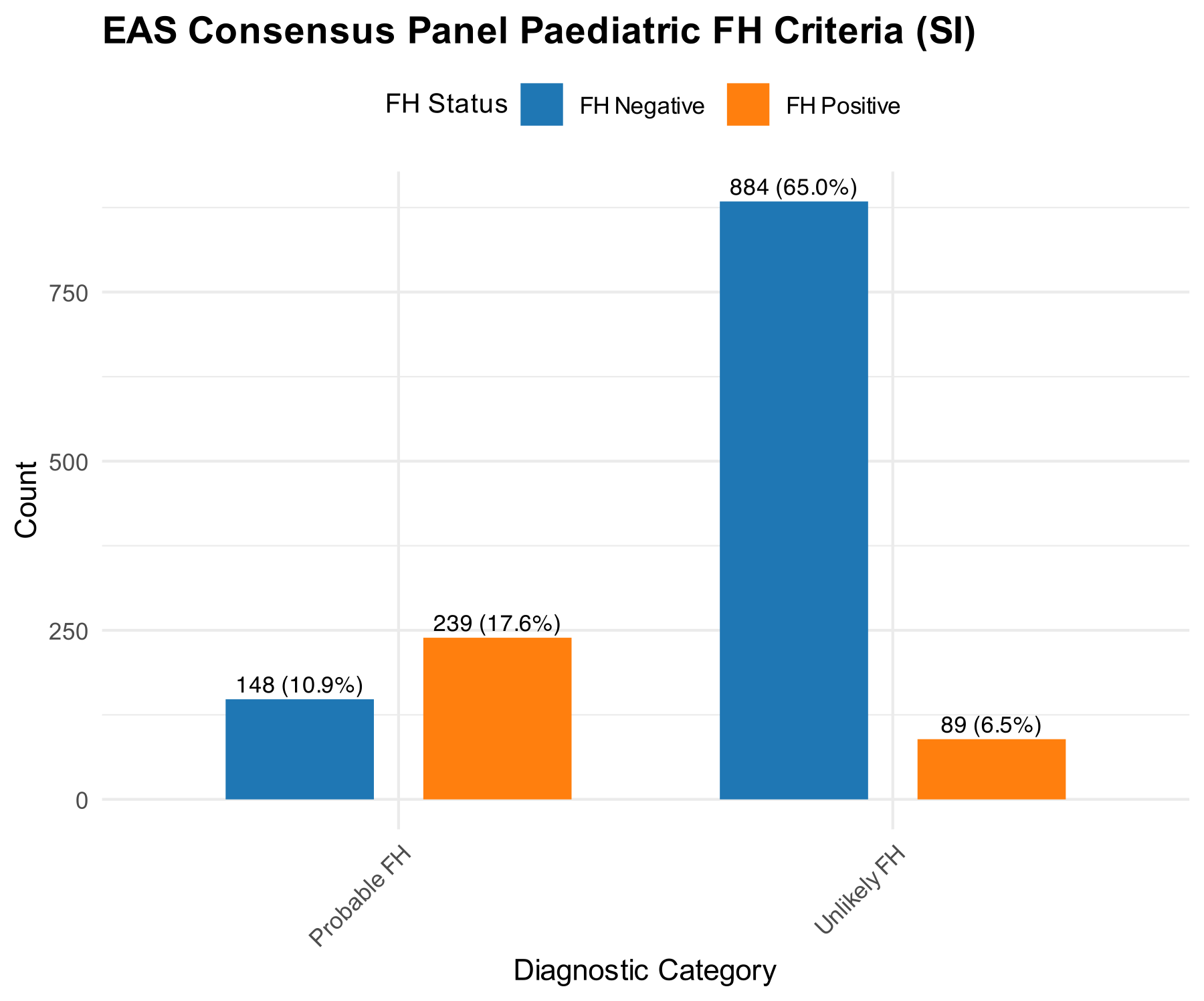

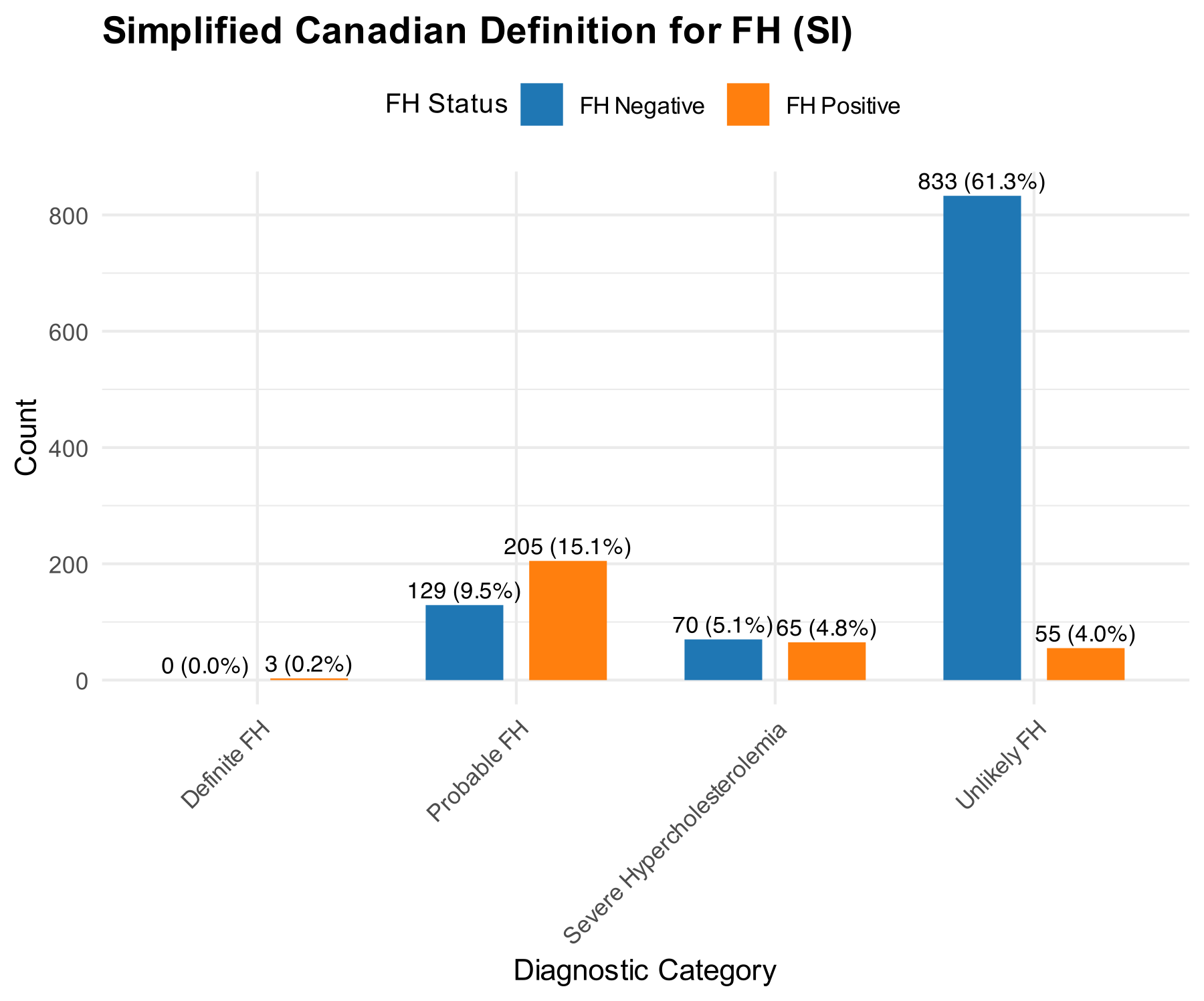

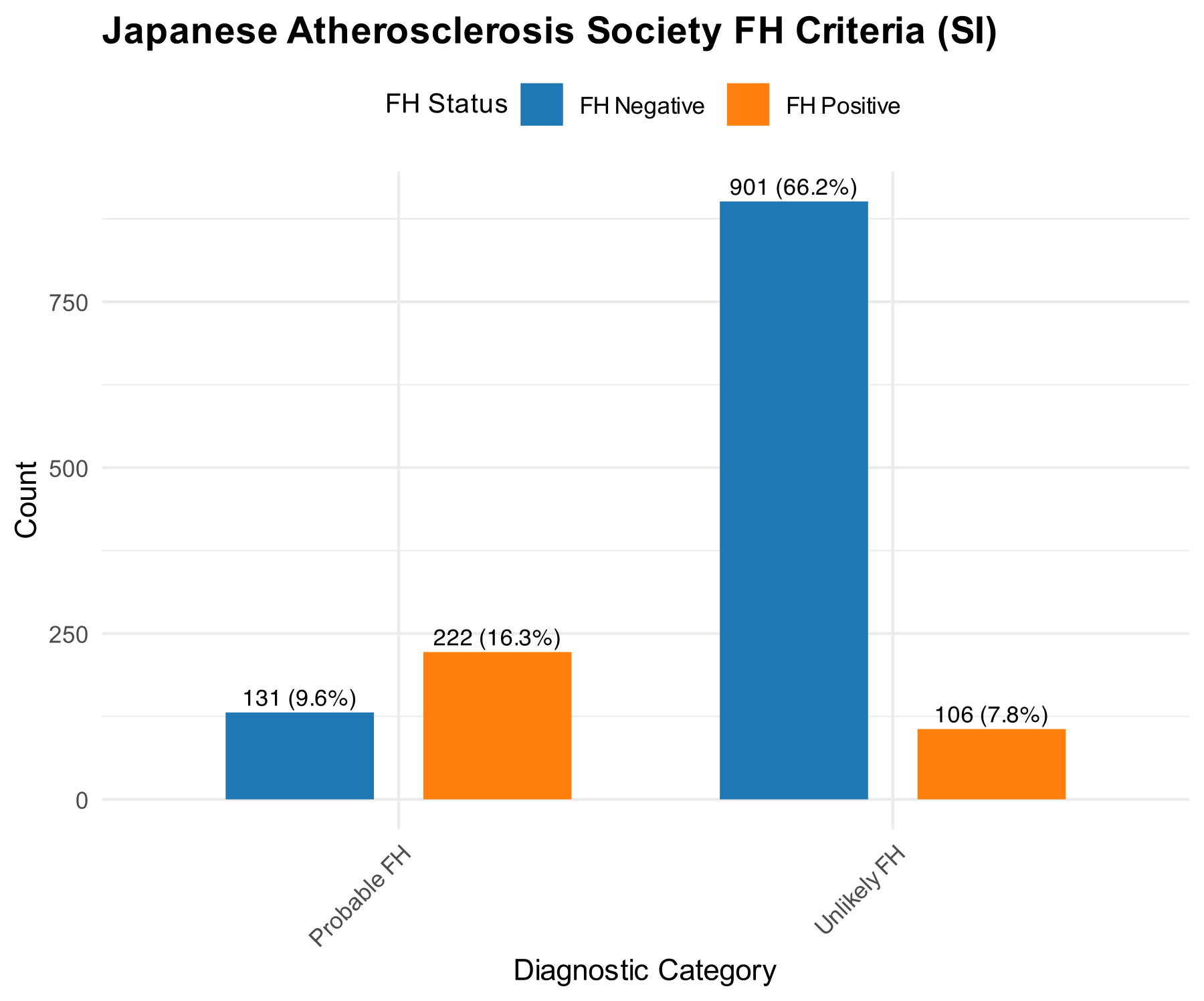


# Figure 2: Bar Plots of Diagnostic Criteria for Familial Hypercholesterolemia (FH) in Portuguese Registry.


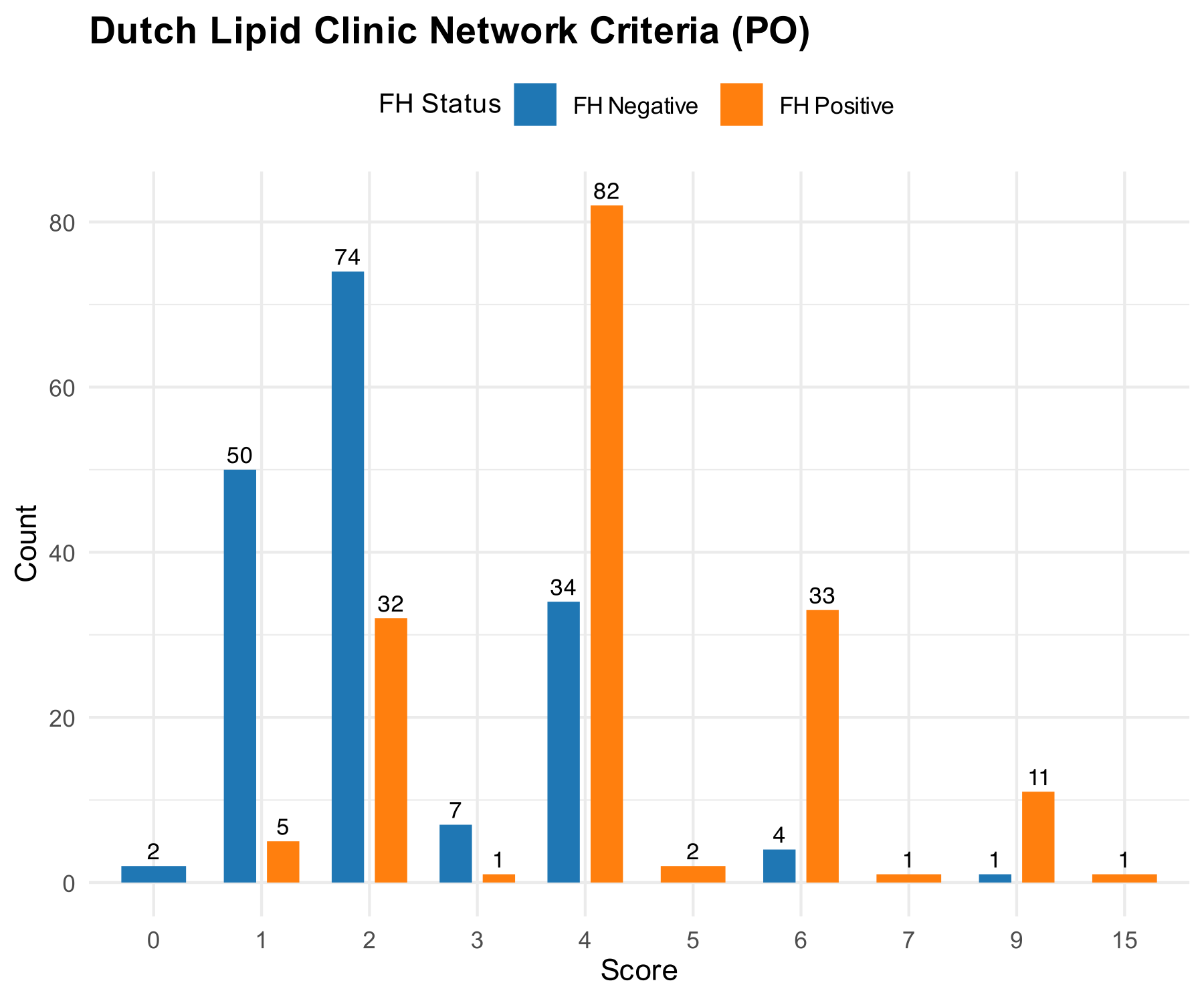

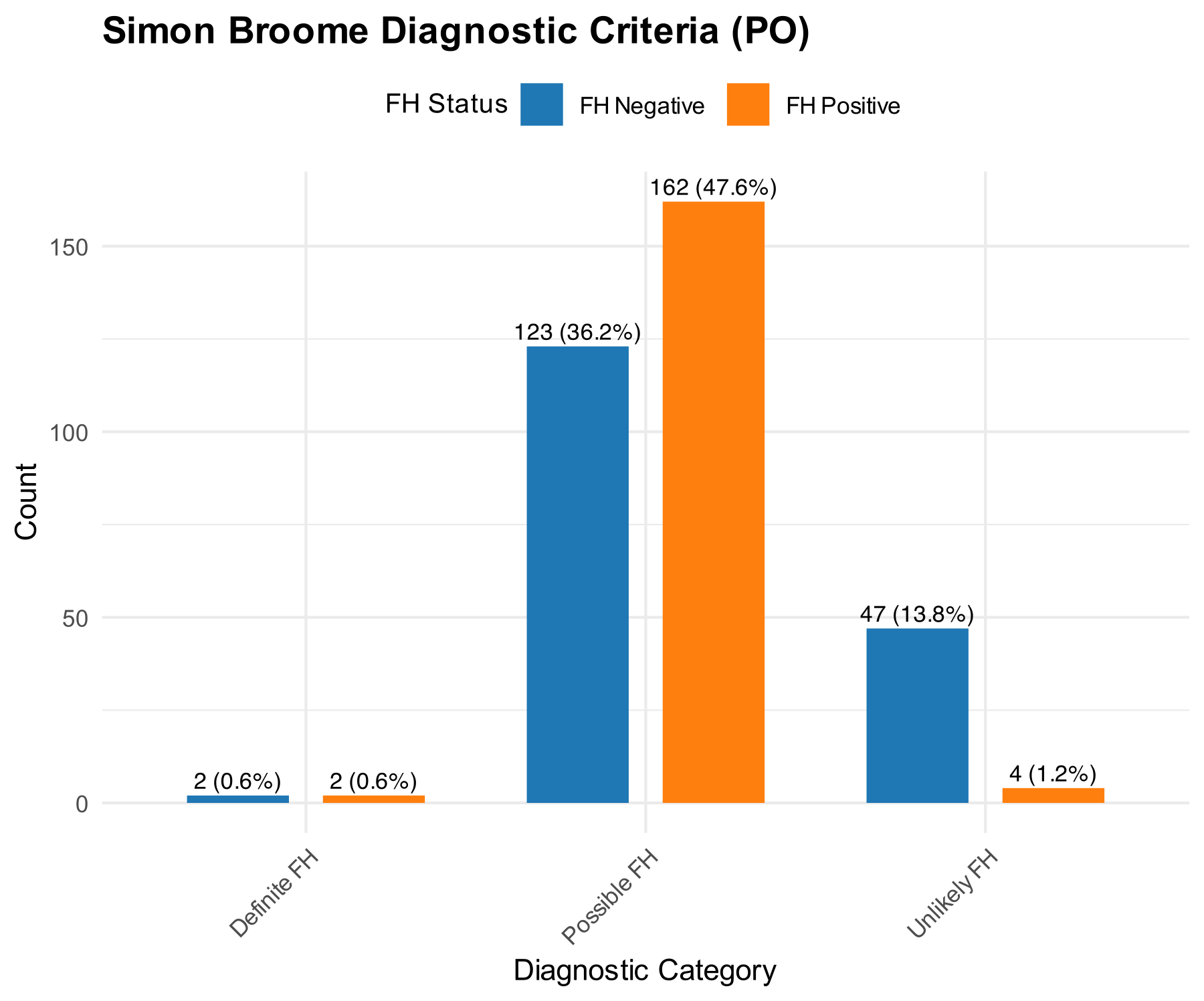

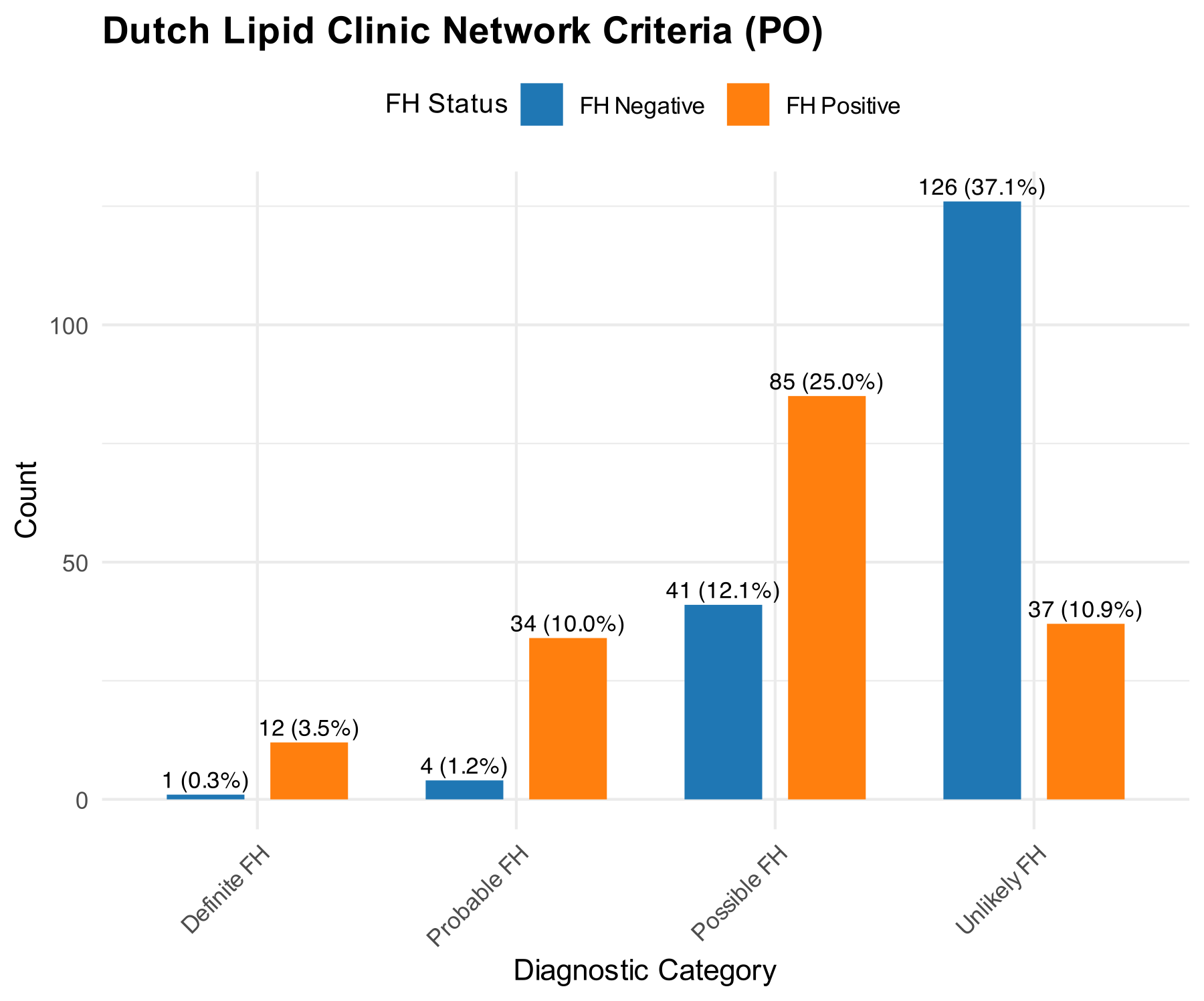

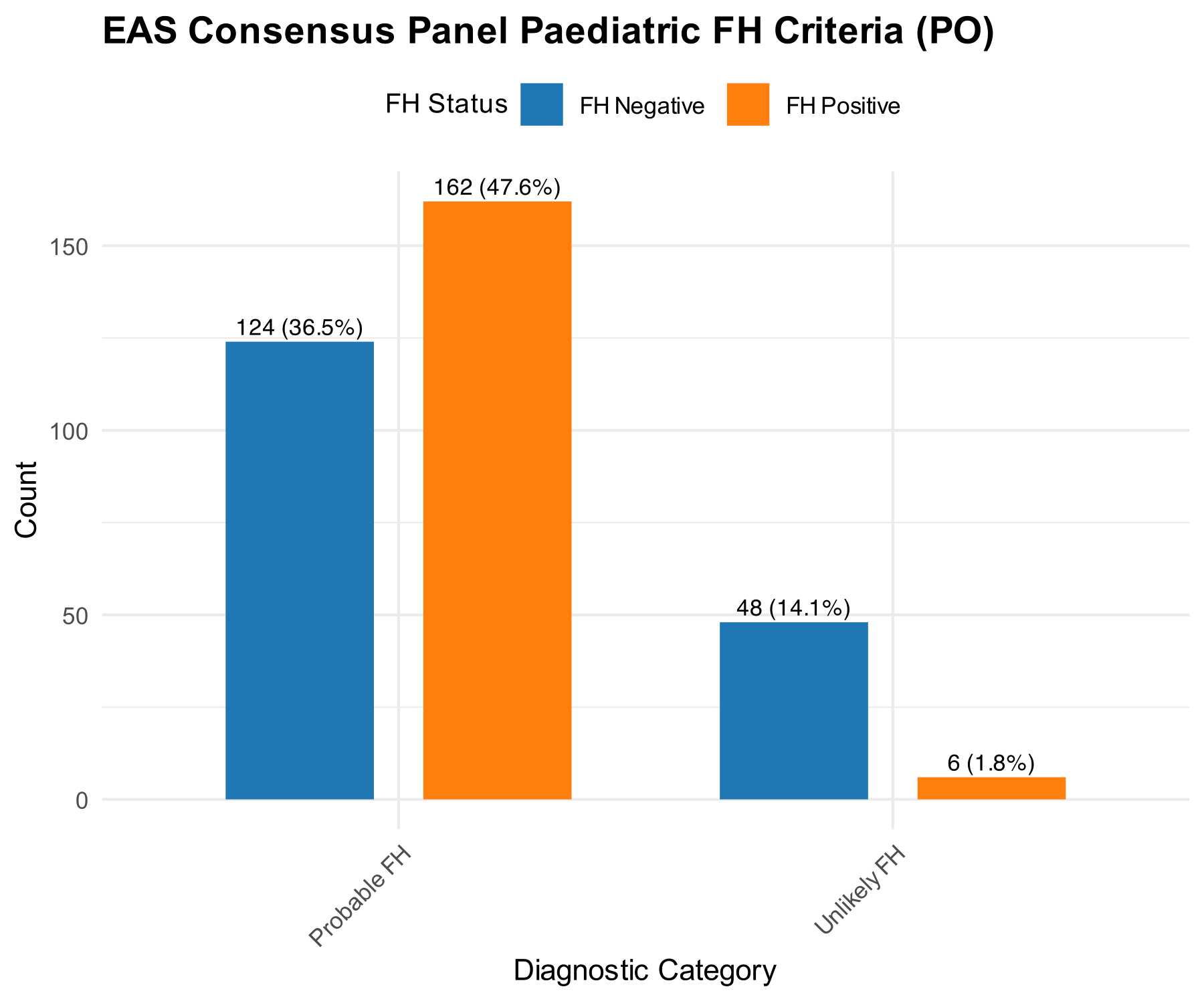

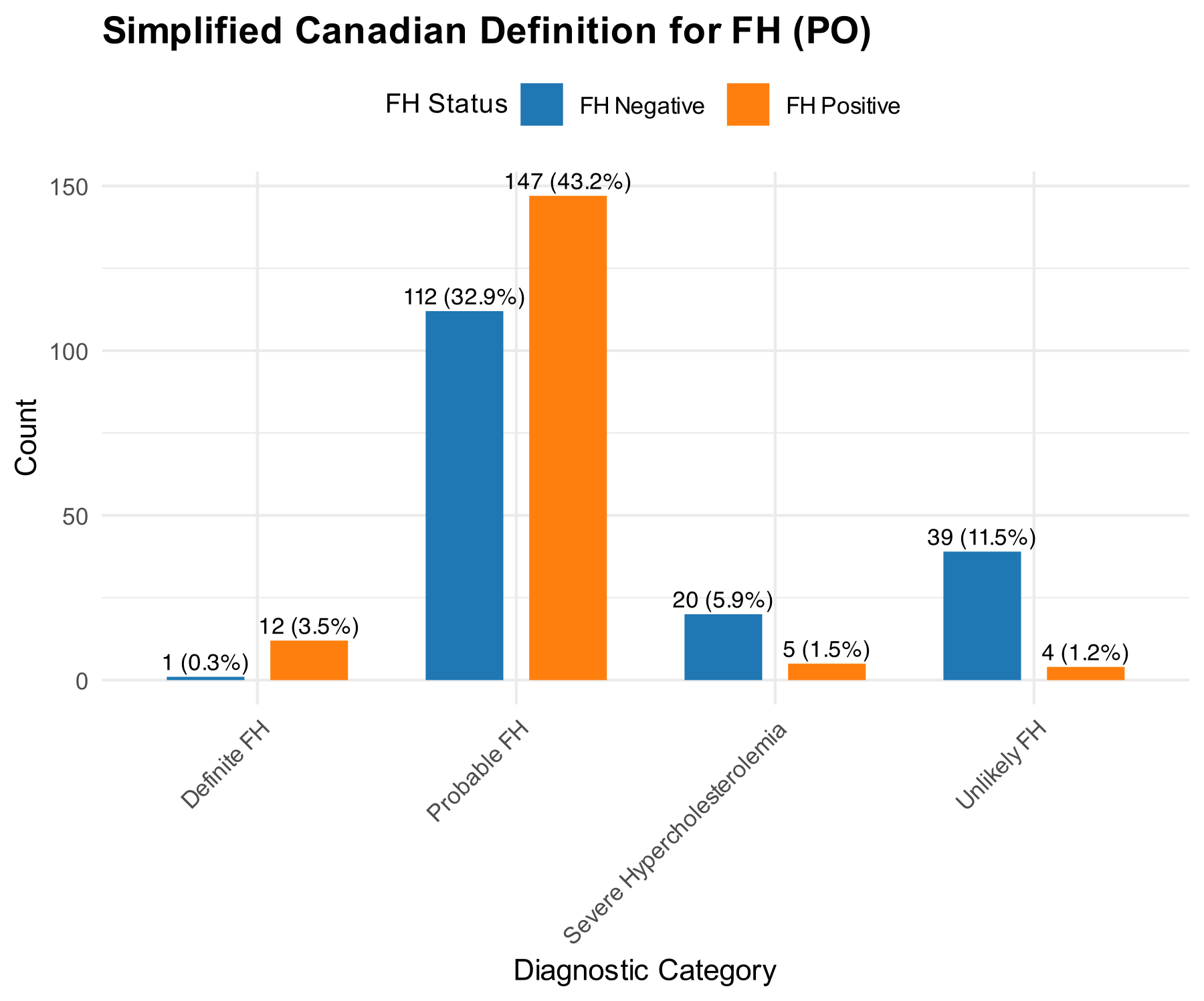

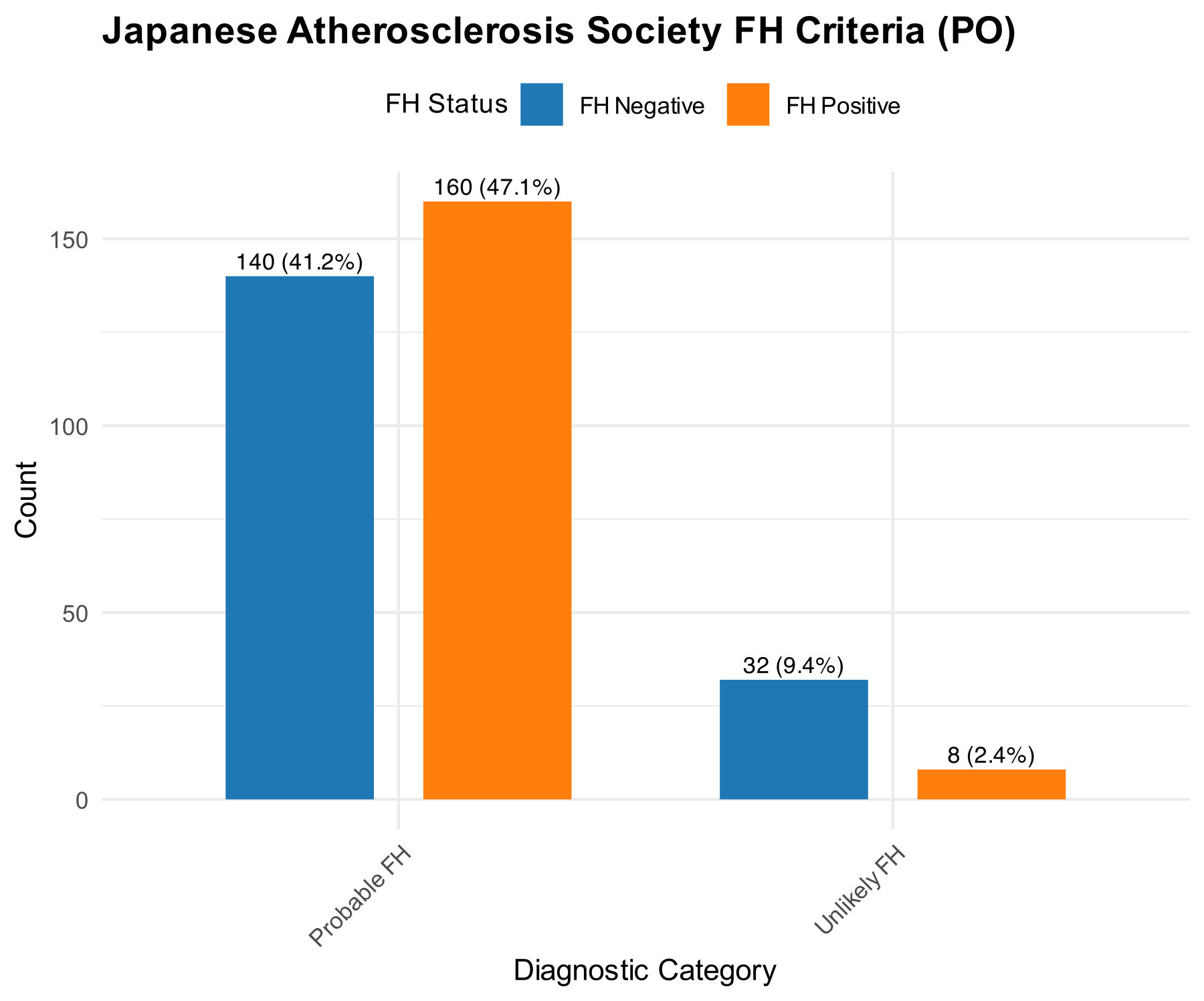


**
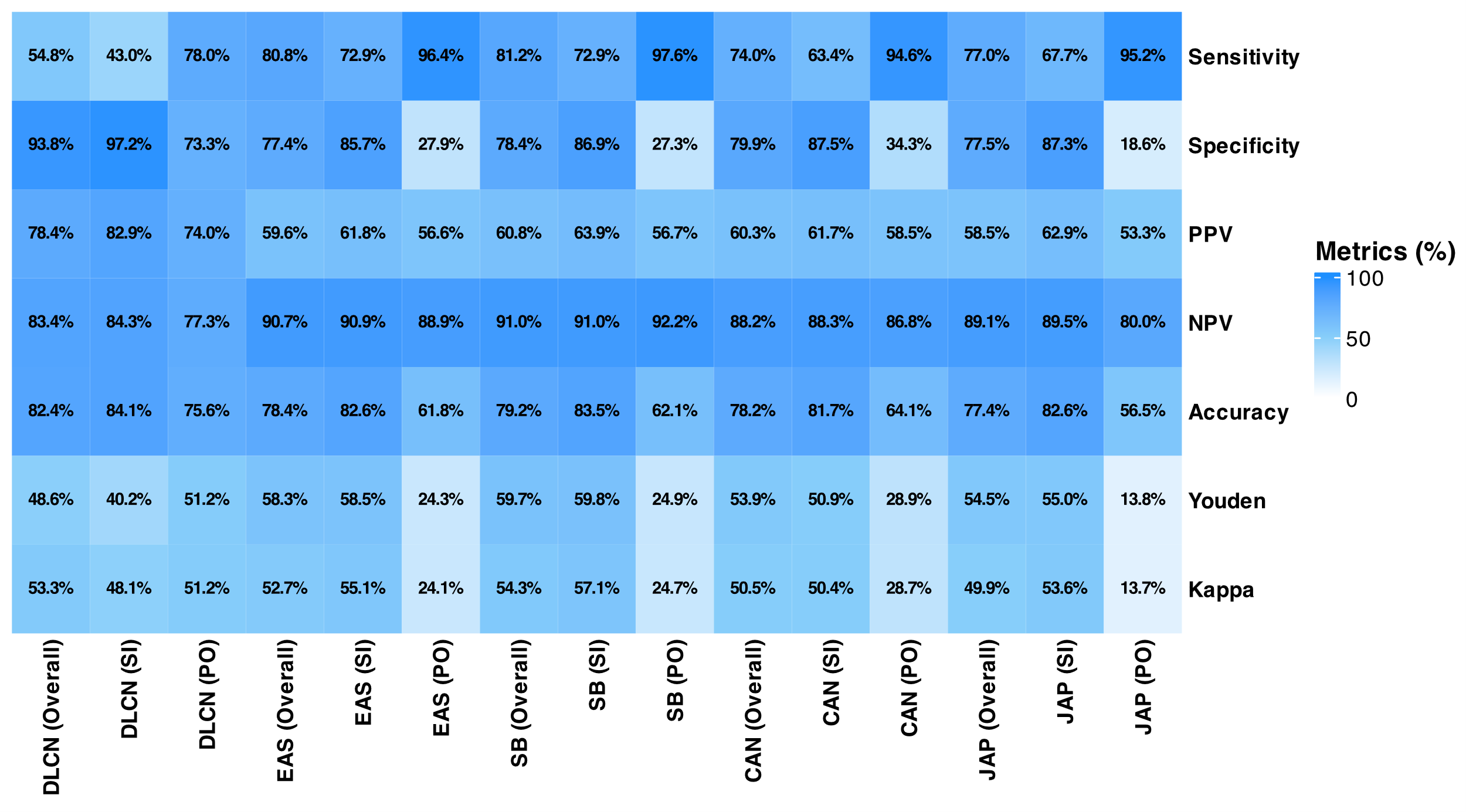
**

Figure 3: Performance Metrics of Diagnostic Criteria for Familial Hypercholesterolemia (FH). Note: The binary scores were created by grouping diagnostic categories: the Dutch Lipid Clinic Network Criteria (DLCN) classified "Definite FH," "Probable FH," and "Possible FH" as positive; the Simon Broome (SB) and Simplified Canadian (CAN) definitions grouped "Definite FH" and "Probable FH" as positive; the Japanese Atherosclerosis Society FH Criteria (JAP) considered "Probable FH" as positive; and the EAS Consensus Panel Pediatric FH Criteria (EAS) labeled "Probable FH" as positive. All other categories were treated as negative, enabling the computation of confusion matrices and performance metrics across these cohorts

**
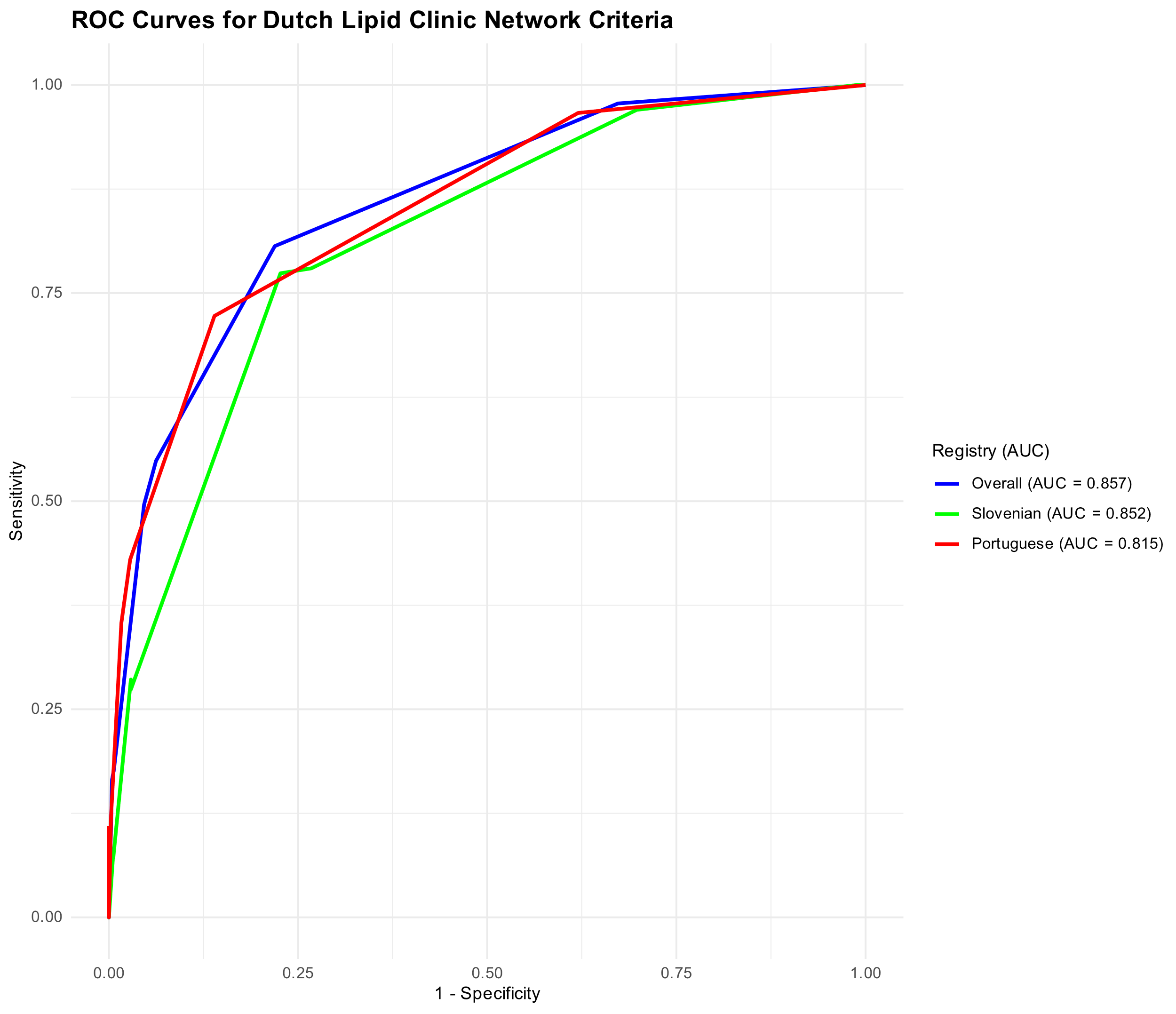
**

Figure 4: Receiver Operating Characteristic Curves of the Dutch Lipid Clinic Network Criteria for Familial Hypercholesterolemia Across Combined (Overall), Slovenian, and Portuguese Registries. Note: Pairwise Area Under the Curve (AUC) comparisons using DeLong's test, adjusted for false discovery rate, showed no significant differences: Combined vs. Slovenian (p = 0.729), Combined vs. Portuguese (p = 0.207), and Slovenian vs. Portuguese (p = 0.207).

Figure 5: Overlap between Diagnostic Criteria for Familial Hypercholesterolemia and Genetically Confirmed Familial Hypercholesterolemia (Combined Registry). Note: This figure compares the overlap between individuals diagnosed as positive for familial hypercholesterolemia using five diagnostic criteria—Dutch Lipid Clinic Network Criteria (DLCN), Simon Broome (SB), Simplified Canadian Definition (CAN), Japanese Atherosclerosis Society Criteria (JAP), and EAS Consensus Panel Criteria (EAS)—and those with genetically confirmed familial hypercholesterolemia (FH). Individuals were considered positive based on the following groupings: DLCN classified "Definite FH," "Probable FH," and "Possible FH" as positive; SB and CAN grouped "Definite FH" and "Probable FH" as positive; JAP considered "Probable FH" as positive; and EAS labeled "Probable FH" as positive. All other categories were treated as negative.


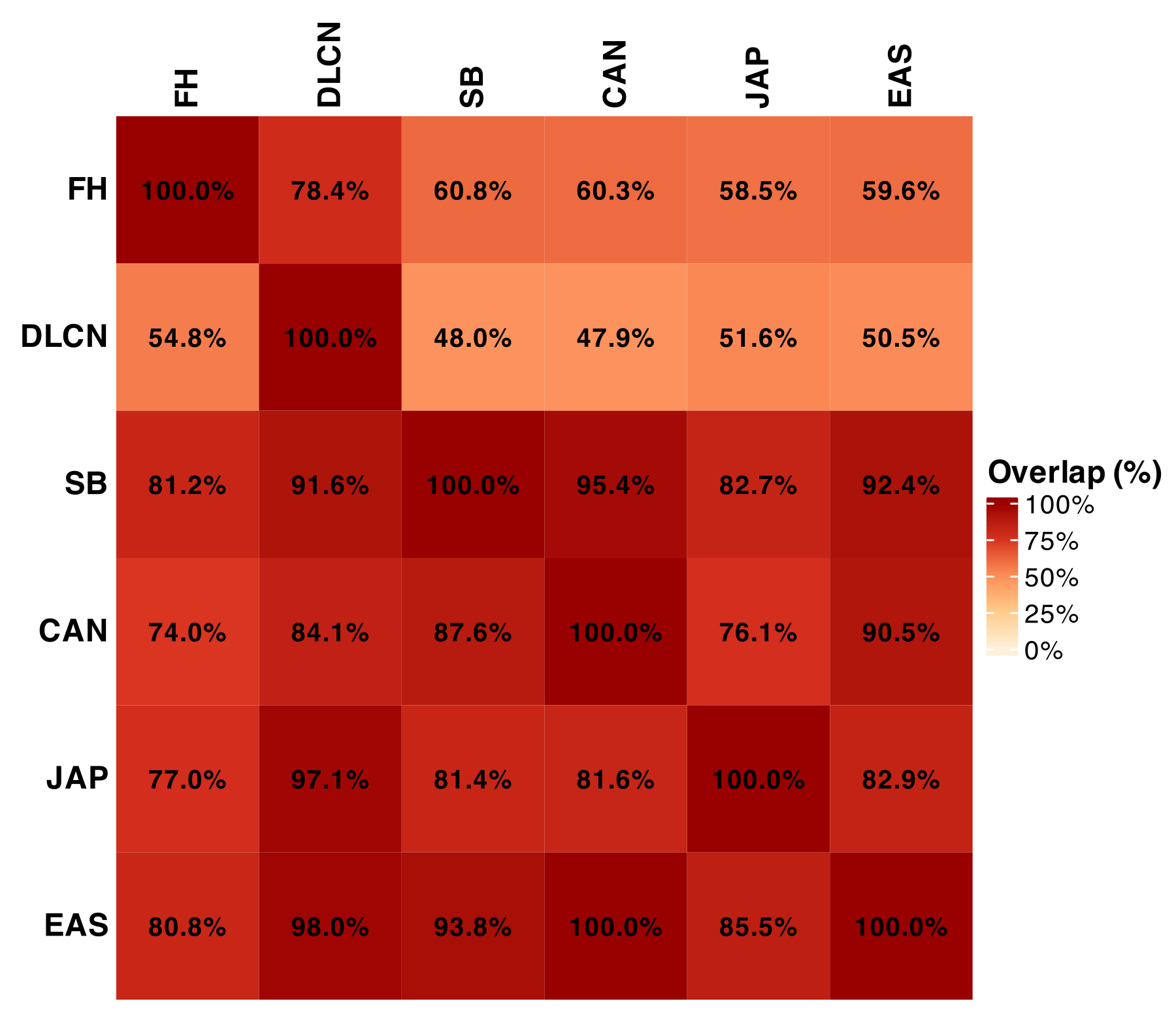

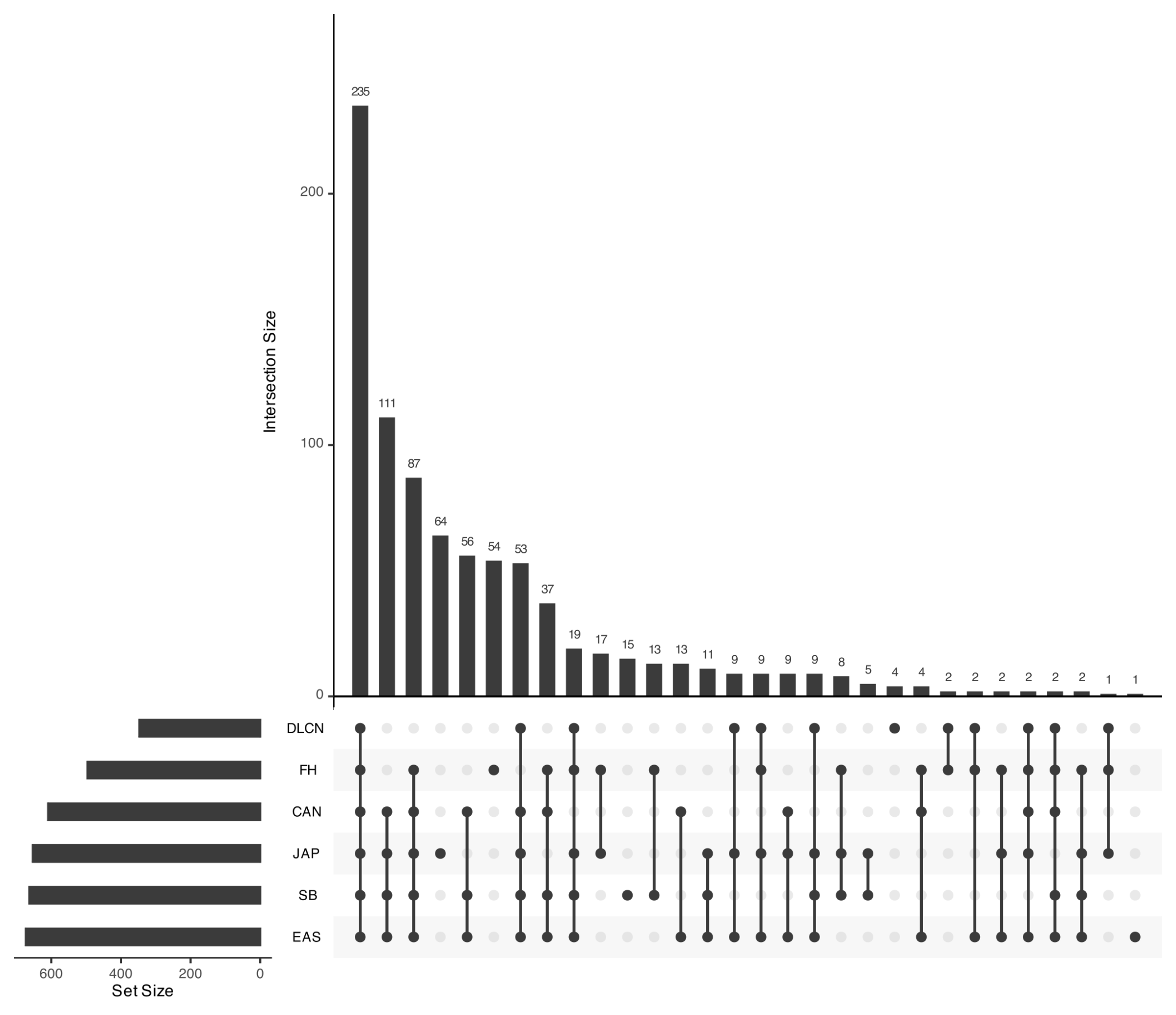

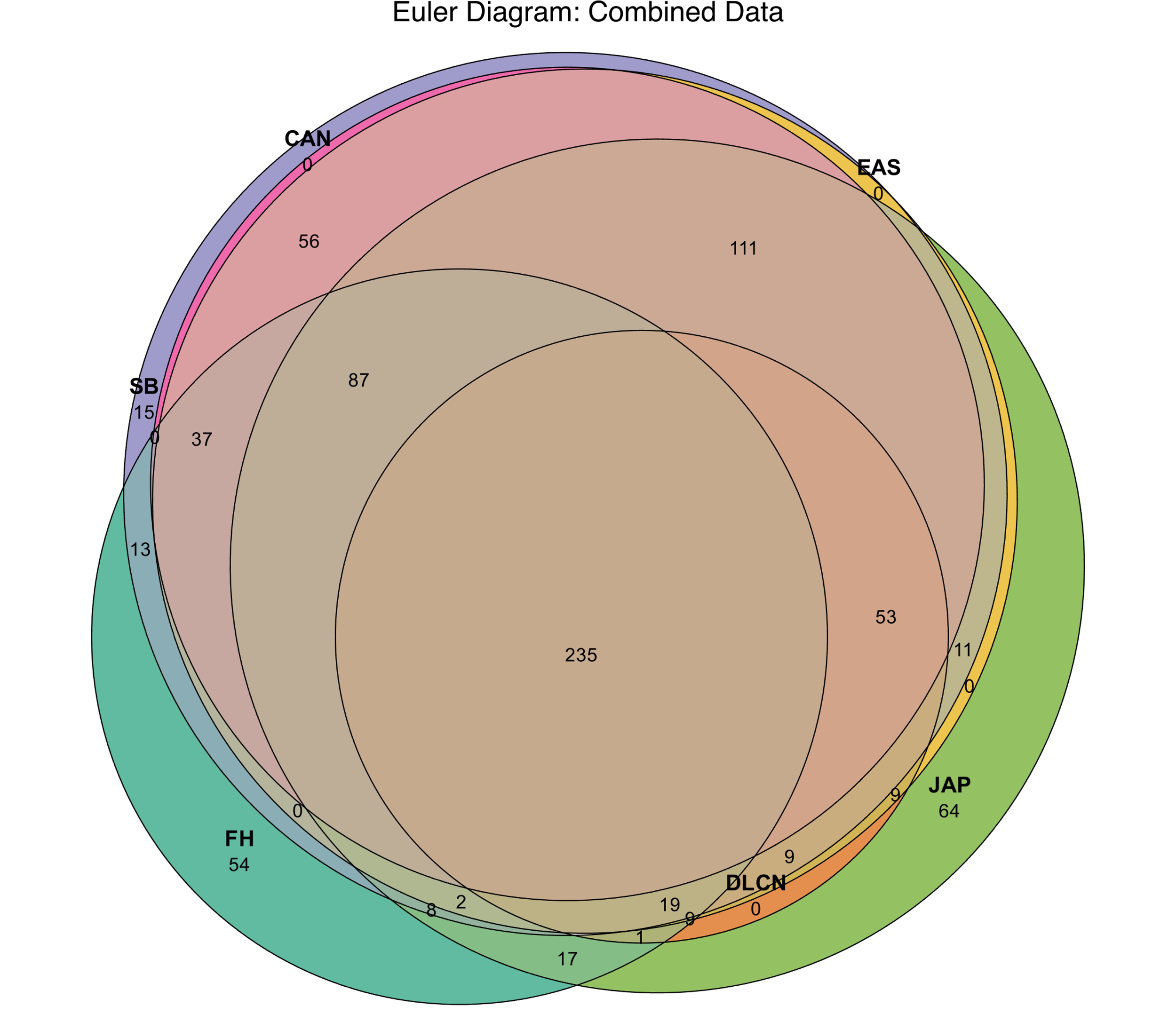

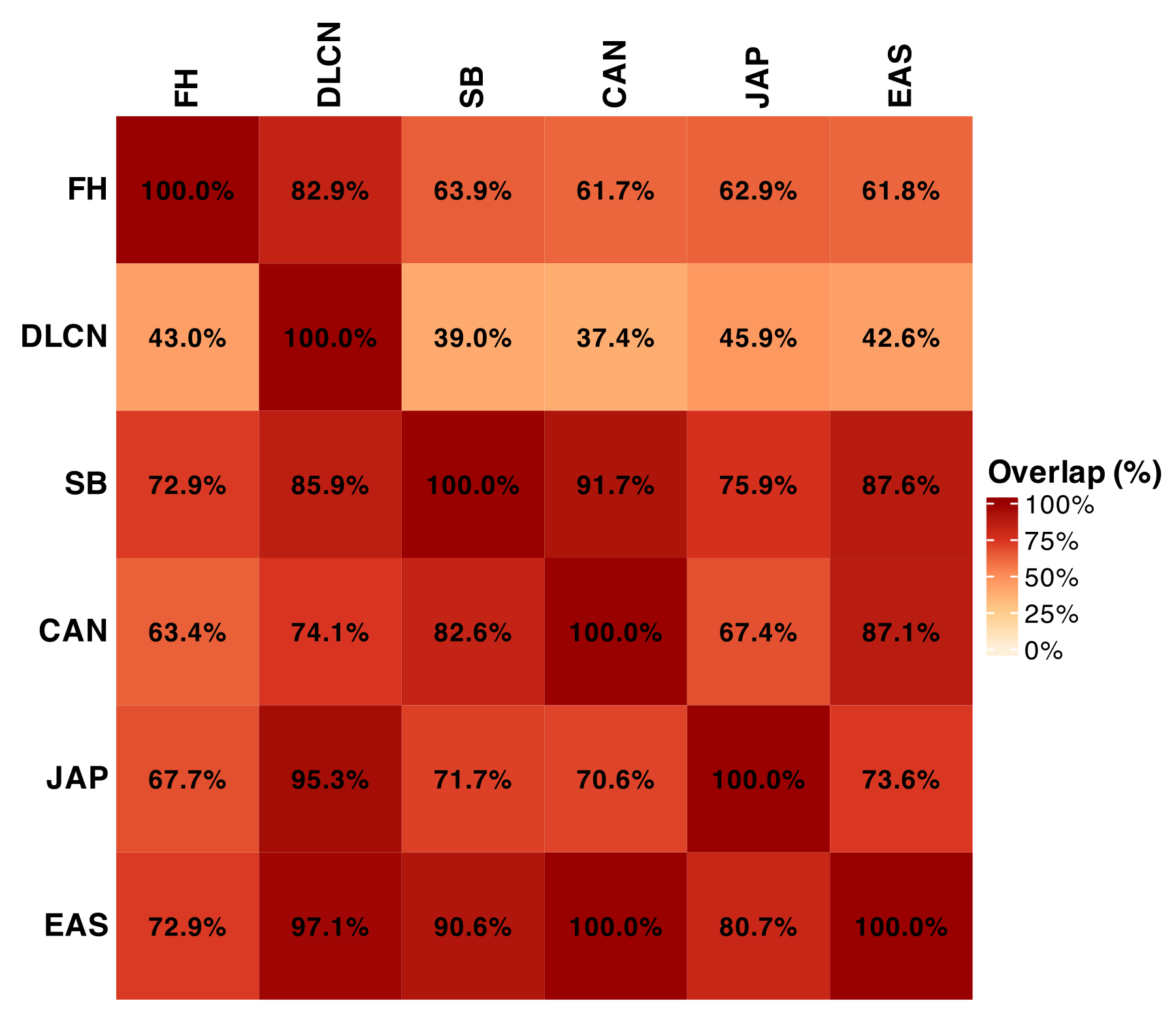

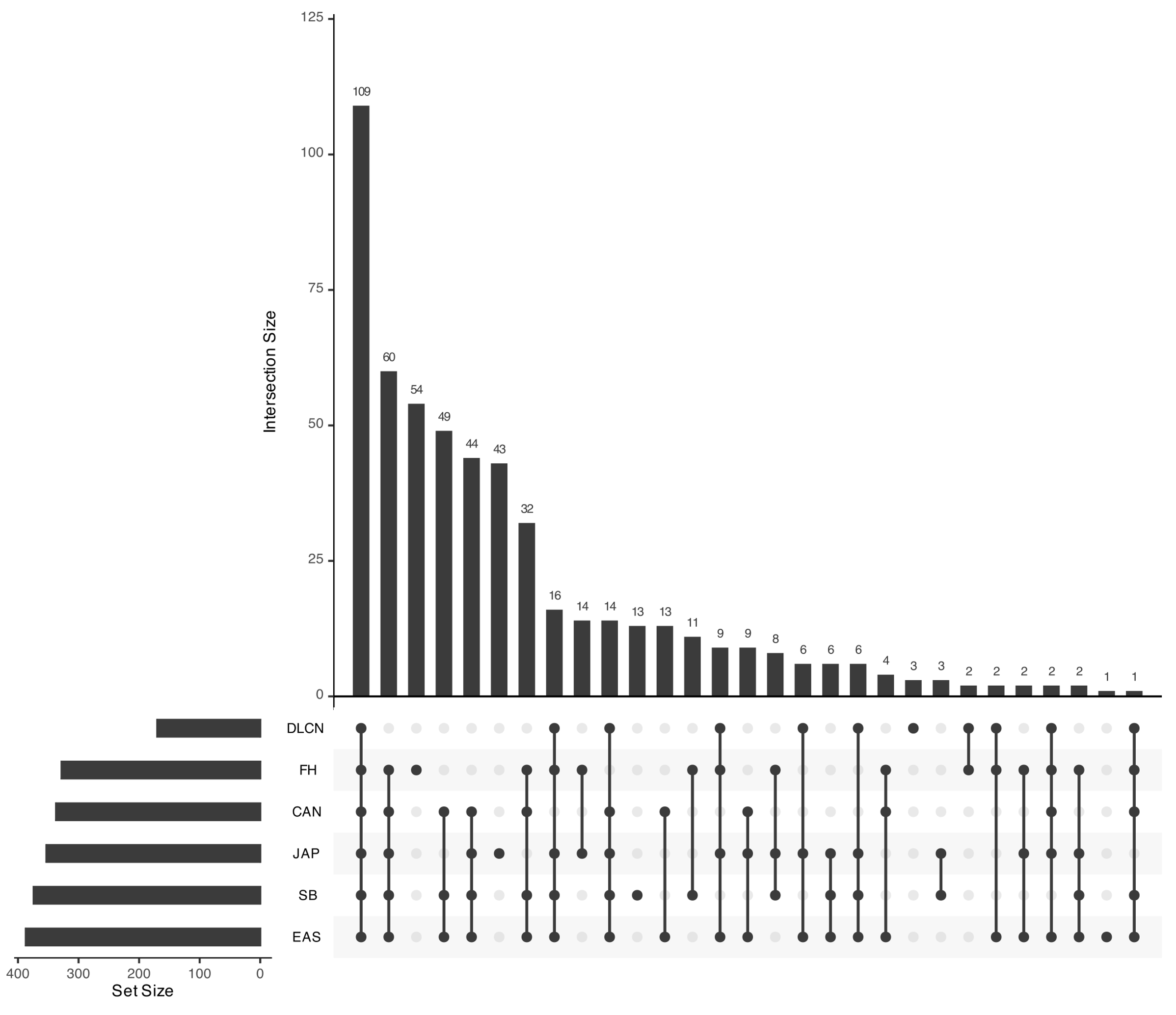

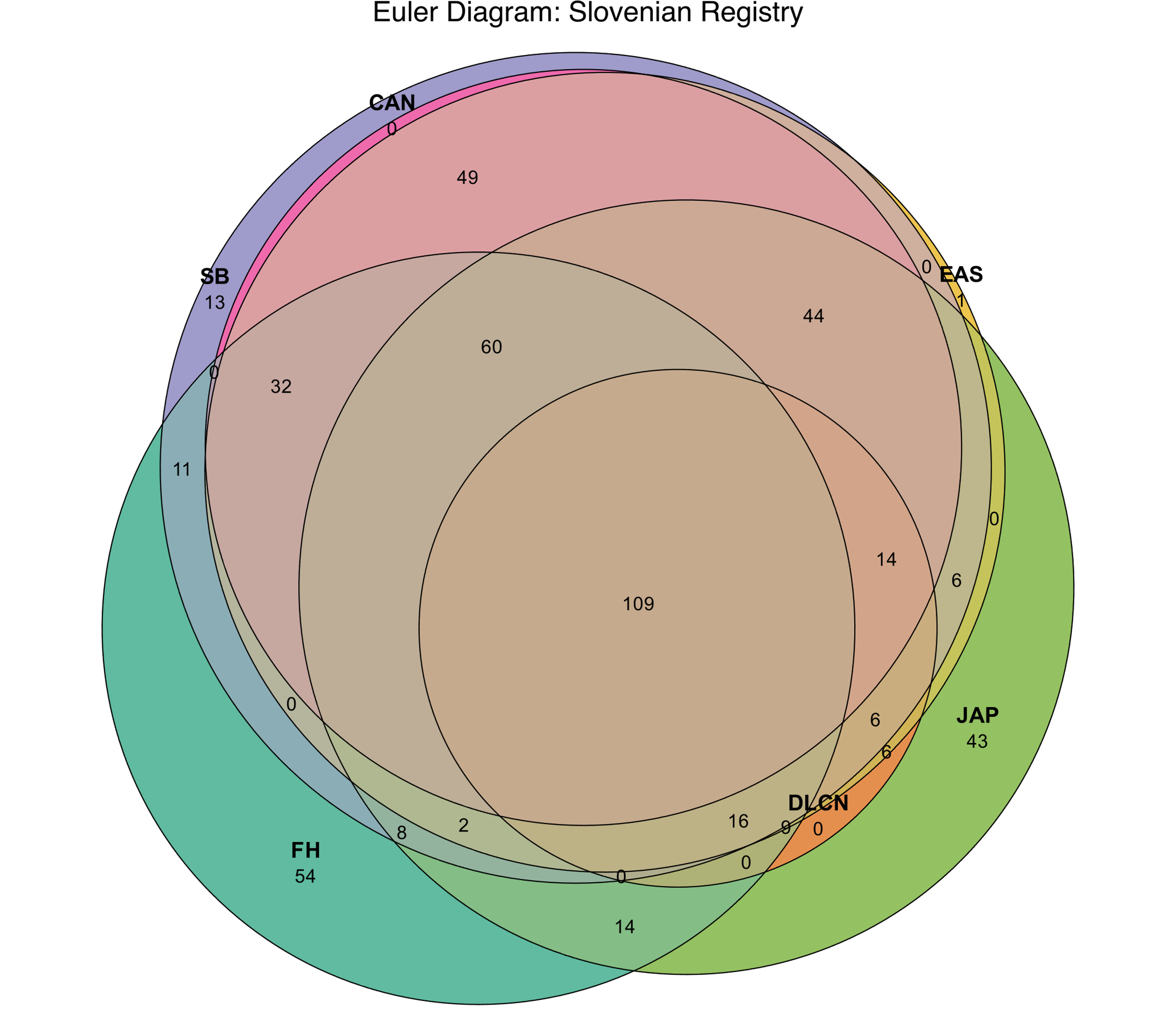


Figure 6: Overlap between Diagnostic Criteria for Familial Hypercholesterolemia and Genetically Confirmed Familial Hypercholesterolemia (Slovenian Registry). Note: This figure compares the overlap between individuals diagnosed as positive for familial hypercholesterolemia using five diagnostic criteria—Dutch Lipid Clinic Network Criteria (DLCN), Simon Broome (SB), Simplified Canadian Definition (CAN), Japanese Atherosclerosis Society Criteria (JAP), and EAS Consensus Panel Criteria (EAS)—and those with genetically confirmed familial hypercholesterolemia (FH). Individuals were considered positive based on the following groupings: DLCN classified "Definite FH," "Probable FH," and "Possible FH" as positive; SB and CAN grouped "Definite FH" and "Probable FH" as positive; JAP considered "Probable FH" as positive; and EAS labeled "Probable FH" as positive. All other categories were treated as negative.


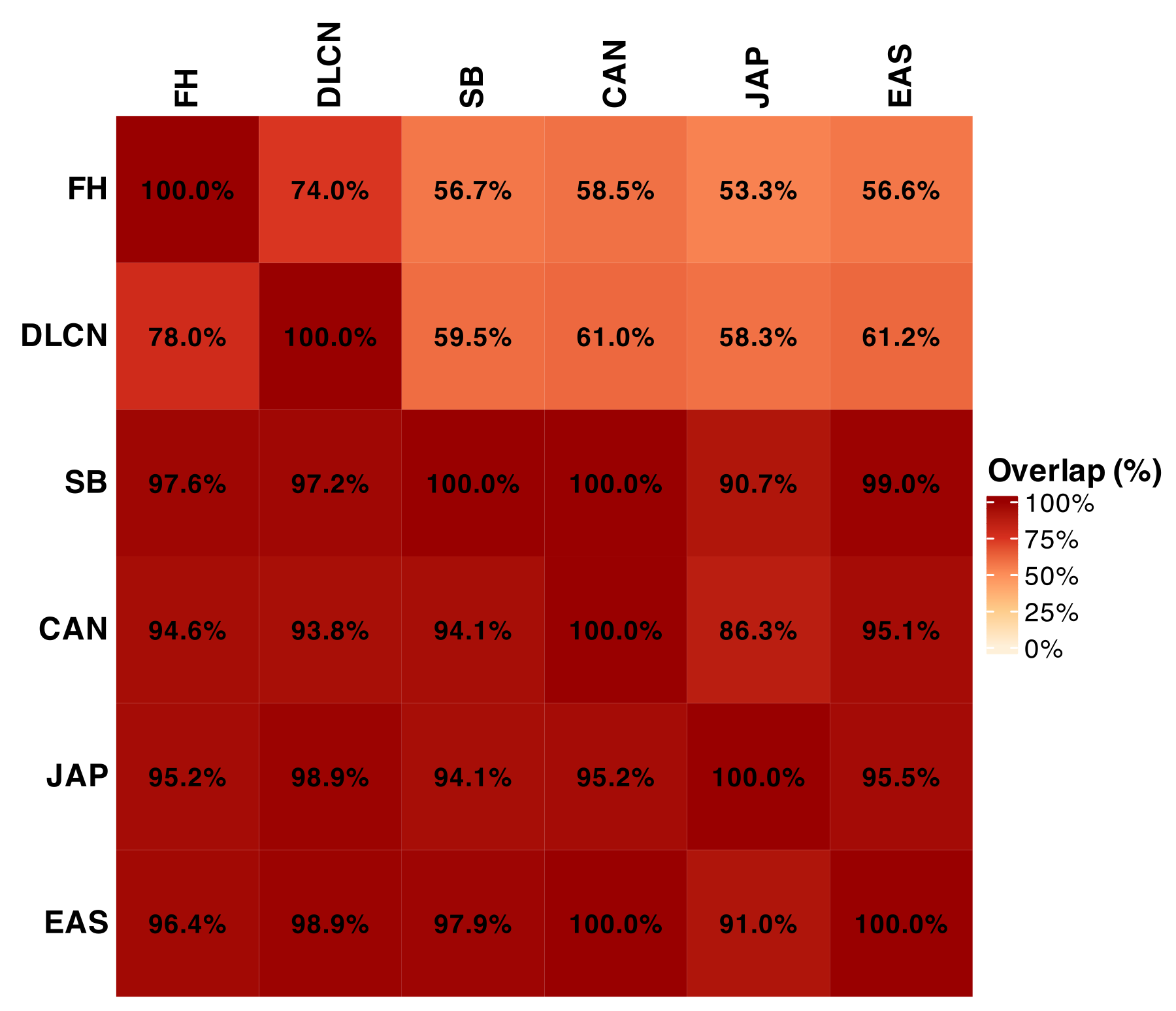

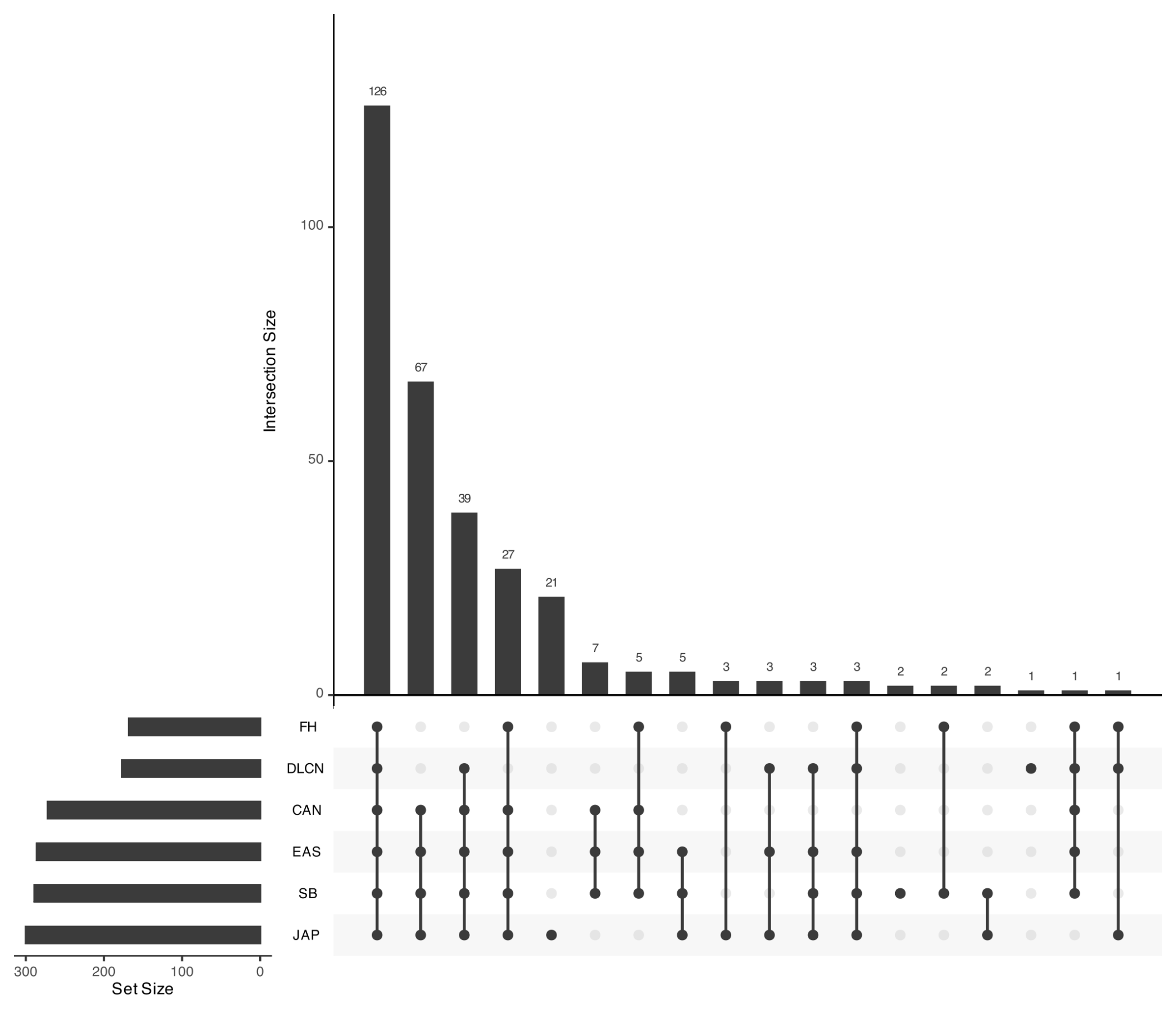

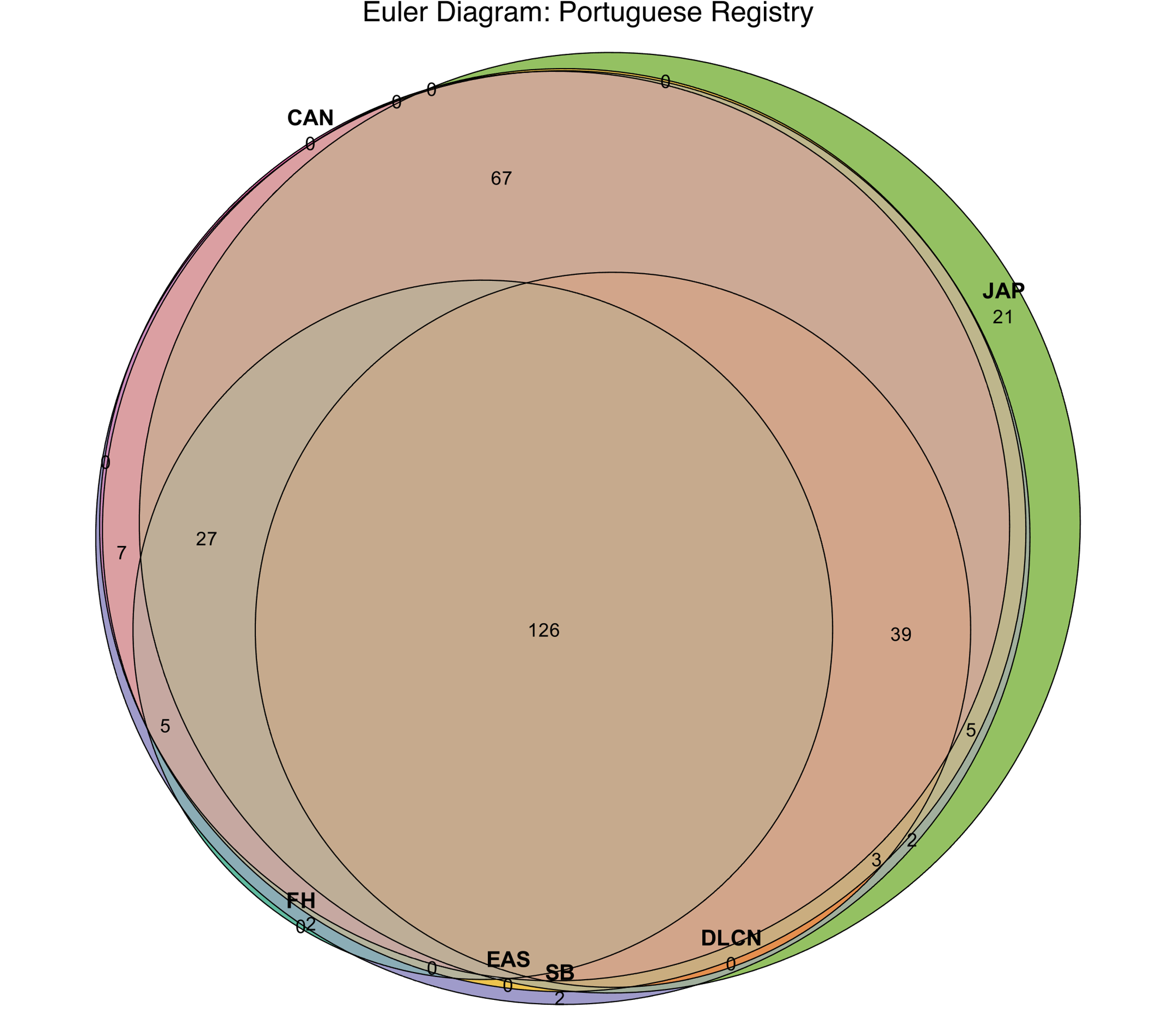


Figure 7: Overlap between Diagnostic Criteria for Familial Hypercholesterolemia and Genetically Confirmed Familial Hypercholesterolemia (Portuguese Registry). Note: This figure compares the overlap between individuals diagnosed as positive for familial hypercholesterolemia using five diagnostic criteria—Dutch Lipid Clinic Network Criteria (DLCN), Simon Broome (SB), Simplified Canadian Definition (CAN), Japanese Atherosclerosis Society Criteria (JAP), and EAS Consensus Panel Criteria (EAS)—and those with genetically confirmed familial hypercholesterolemia (FH). Individuals were considered positive based on the following groupings: DLCN classified "Definite FH," "Probable FH," and "Possible FH" as positive; SB and CAN grouped "Definite FH" and "Probable FH" as positive; JAP considered "Probable FH" as positive; and EAS labeled "Probable FH" as positive. All other categories were treated as negative.

**
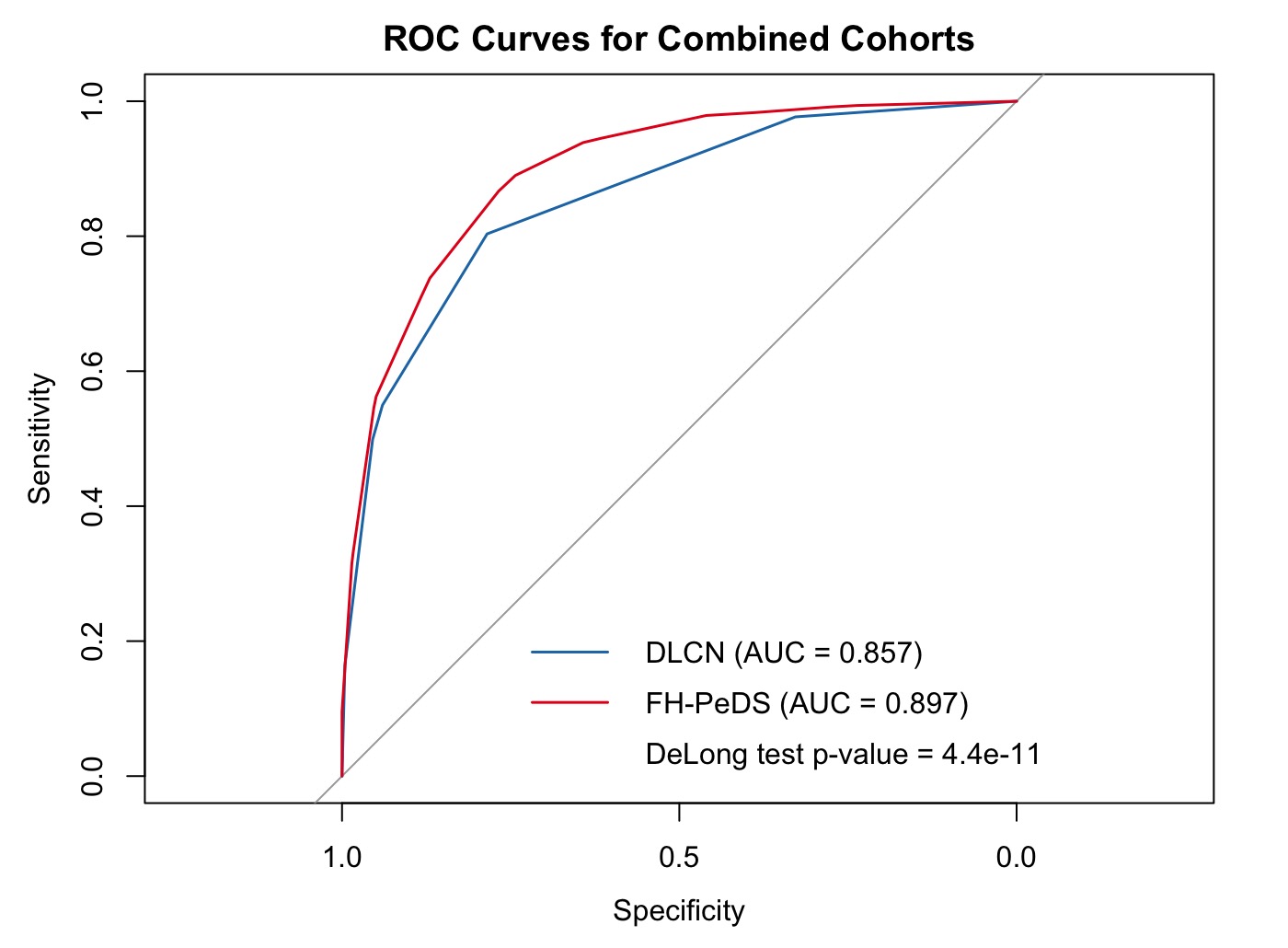
**

# Figure 8: Receiver Operating Characteristic (ROC) Curves Comparing the Dutch Lipid Clinic Network Score (DLCN) and the New FH Score for Familial Hypercholesterolemia Diagnosis (FH-PeDS) in the Combined Cohort.

**
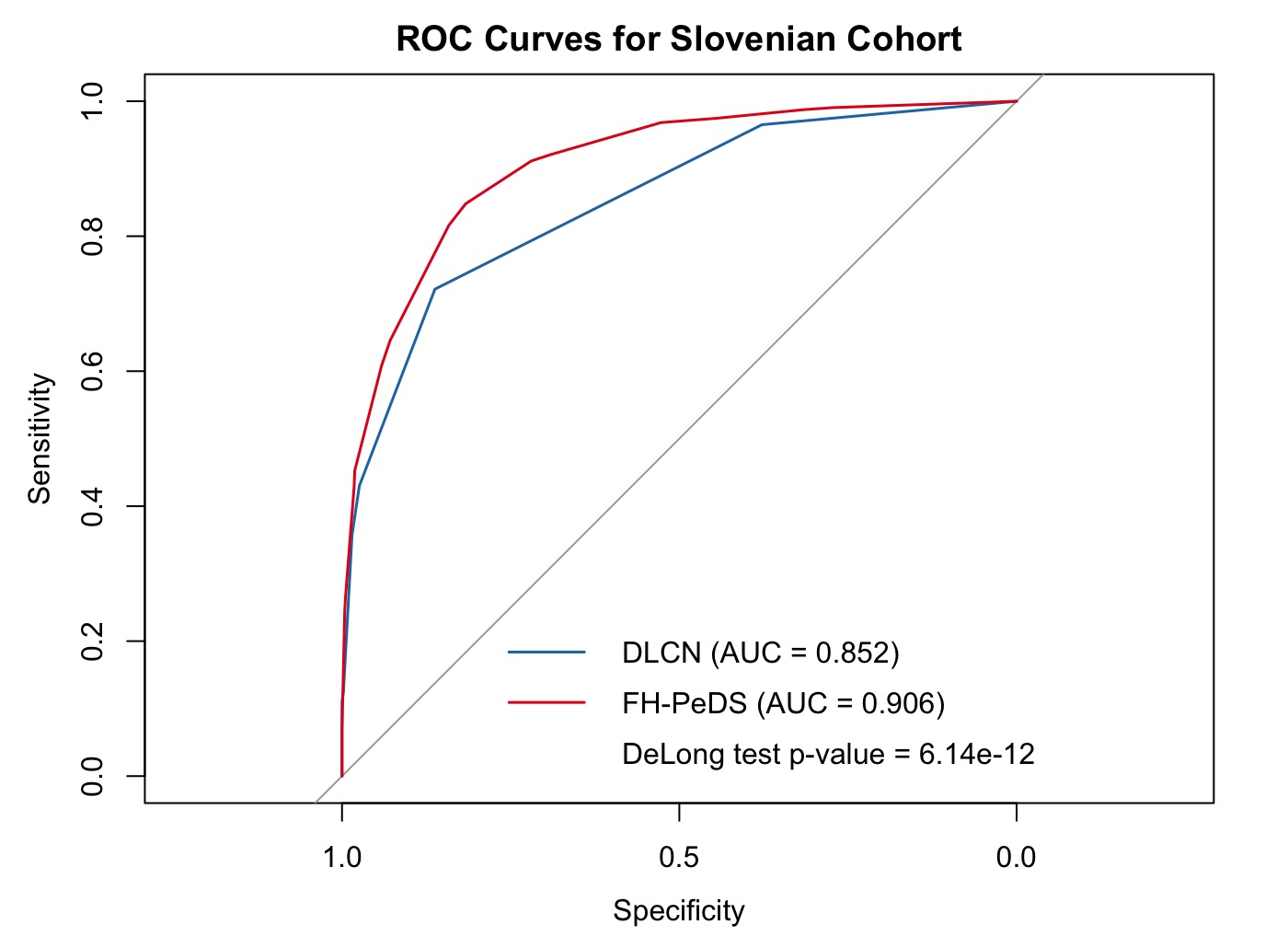
**

# Figure 9: Receiver Operating Characteristic (ROC) Curves Comparing the Dutch Lipid Clinic Network Score (DLCN) and the New FH Score for Familial Hypercholesterolemia Diagnosis (FH-PeDS) in the Slovenian Cohort.

***
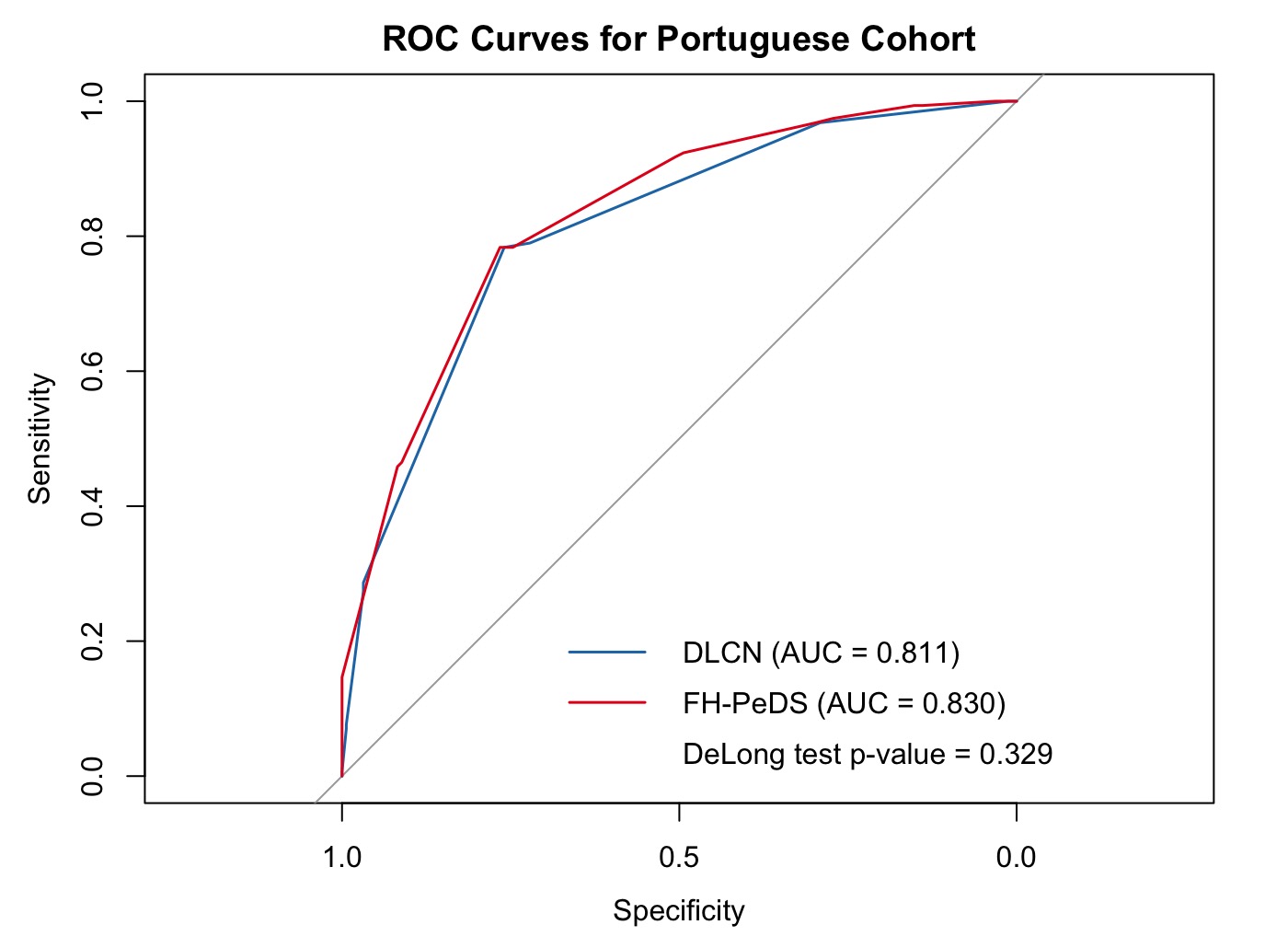
***

# Figure 10: Receiver Operating Characteristic (ROC) Curves Comparing the Dutch Lipid Clinic Network Score (DLCNS) and the New FH Score for Familial Hypercholesterolemia Diagnosis (FH-PeDS) in the Portuguese Cohort.

***
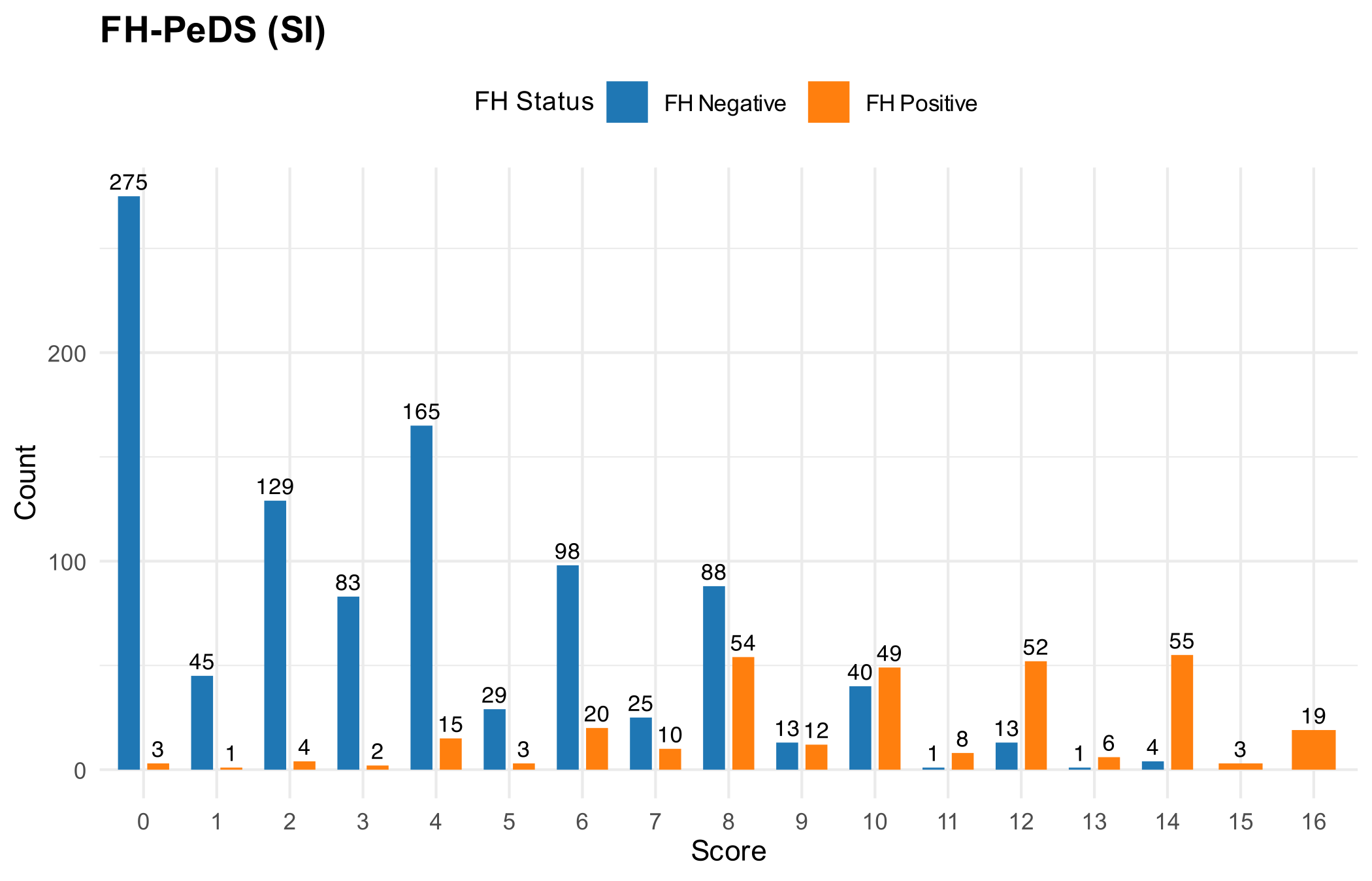
***

# Figure 11: Distribution of FH Pediatric Score (FH-PeDS) in Subjects with and Without Familial Hypercholesterolemia in the Slovenian Cohort.


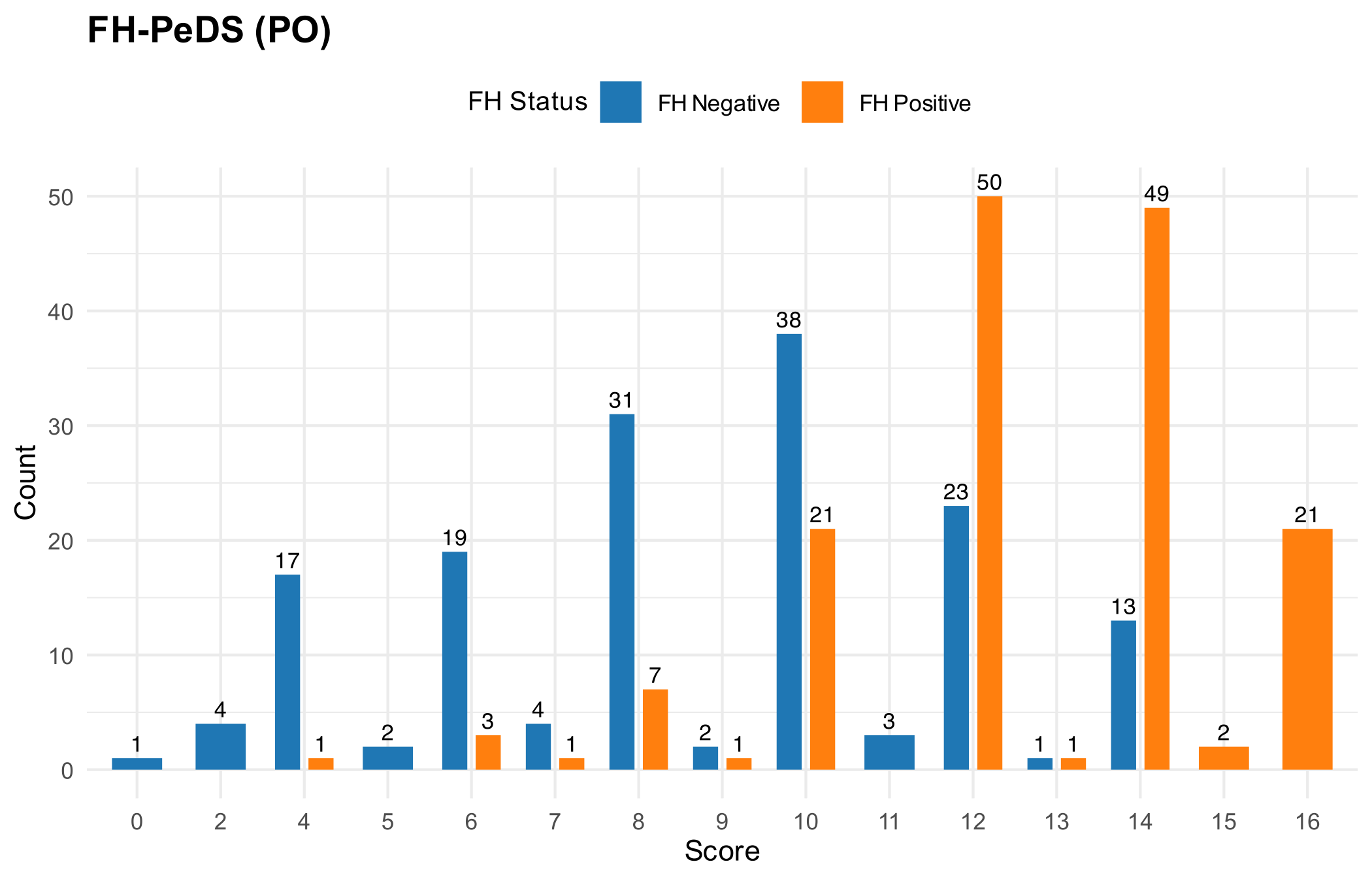


# Figure 12: Distribution of FH Pediatric Score (FH-PeDS) in Subjects with and Without Familial Hypercholesterolemia in the Portuguese Cohort.

# References

1. Desvignes JP, Bartoli M, Delague V, Krahn M, Miltgen M, Beroud C, et al. VarAFT: a variant annotation and filtration system for human next generation sequencing data. Nucleic Acids Res. 2018;46(W1):W545-W53.

2. Schwarz JM, Cooper DN, Schuelke M, Seelow D. MutationTaster2: mutation prediction for the deep-sequencing age. Nat Methods. 2014;11(4):361-2.

3. Adzhubei IA, Schmidt S, Peshkin L, Ramensky VE, Gerasimova A, Bork P, et al. A method and server for predicting damaging missense mutations. Nat Methods. 2010;7(4):248-9.

4. Ng PC, Henikoff S. Predicting deleterious amino acid substitutions. Genome Res. 2001;11(5):863-74.

5. Kircher M, Witten DM, Jain P, O'Roak BJ, Cooper GM, Shendure J. A general framework for estimating the relative pathogenicity of human genetic variants. Nat Genet. 2014;46(3):310-5.

6. Ioannidis NM, Rothstein JH, Pejaver V, Middha S, McDonnell SK, Baheti S, et al. REVEL: An Ensemble Method for Predicting the Pathogenicity of Rare Missense Variants. Am J Hum Genet. 2016;99(4):877-85.

7. Desmet FO, Hamroun D, Lalande M, Collod-Beroud G, Claustres M, Beroud C. Human Splicing Finder: an online bioinformatics tool to predict splicing signals. Nucleic Acids Res. 2009;37(9):e67.

8. Richards S, Aziz N, Bale S, Bick D, Das S, Gastier-Foster J, et al. Standards and guidelines for the interpretation of sequence variants: a joint consensus recommendation of the American College of Medical Genetics and Genomics and the Association for Molecular Pathology. Genet Med. 2015;17(5):405-24.

9. Chora JR, Iacocca MA, Tichy L, Wand H, Kurtz CL, Zimmermann H, et al. The Clinical Genome Resource (ClinGen) Familial Hypercholesterolemia Variant Curation Expert Panel consensus guidelines for LDLR variant classification. Genet Med. 2022;24(2):293-306.

10. Freeman PJ, Hart RK, Gretton LJ, Brookes AJ, Dalgleish R. VariantValidator: Accurate validation, mapping, and formatting of sequence variation descriptions. Hum Mutat. 2018;39(1):61-8.

11. Landrum MJ, Lee JM, Riley GR, Jang W, Rubinstein WS, Church DM, et al. ClinVar: public archive of relationships among sequence variation and human phenotype. Nucleic Acids Res. 2014;42(Database issue):D980-5.

12. Chora JR, Medeiros AM, Alves AC, Bourbon M. Analysis of publicly available LDLR, APOB, and PCSK9 variants associated with familial hypercholesterolemia: application of ACMG guidelines and implications for familial hypercholesterolemia diagnosis. Genet Med. 2018;20(6):591-8.

13. Risk of fatal coronary heart disease in familial hypercholesterolaemia. Scientific Steering Committee on behalf of the Simon Broome Register Group. BMJ. 1991;303(6807):893-6.

14. Wiegman A, Gidding SS, Watts GF, Chapman MJ, Ginsberg HN, Cuchel M, et al. Familial hypercholesterolaemia in children and adolescents: gaining decades of life by optimizing detection and treatment. Eur Heart J. 2015;36(36):2425-37.

15. Programme WHOHG. Familial hypercholesterolaemia (FH) : report of a second WHO consultation, Geneva, 4 September 1998. Geneva: World Health Organization; 1999.

16. Ruel I, Brisson D, Aljenedil S, Awan Z, Baass A, Belanger A, et al. Simplified Canadian Definition for Familial Hypercholesterolemia. Can J Cardiol. 2018;34(9):1210-4.

17. Dobashi K. Japanese Diagnostic Criteria for Pediatric Familial Hypercholesterolemia 2022. J Atheroscler Thromb. 2024;31(7):1026-8.

18. Harada-Shiba M, Arai H, Ishigaki Y, Ishibashi S, Okamura T, Ogura M, et al. Guidelines for Diagnosis and Treatment of Familial Hypercholesterolemia 2017. J Atheroscler Thromb. 2018;25(8):751-70.

19. H. W. Ggplot2: Elegant graphics for data analysis. 2 ed. Cham, Switzerland: Springer International Publishing; 2016 jun.
